# Human Resources for Health-Related Challenges to Ensuring Quality Newborn Care in Low- and Middle-Income Countries: A Scoping Review

**DOI:** 10.9745/GHSP-D-20-00362

**Published:** 2021-03-31

**Authors:** Nancy Bolan, Karen D. Cowgill, Karen Walker, Lily Kak, Theresa Shaver, Sarah Moxon, Ornella Lincetto

**Affiliations:** a Office of Global Health, University of Maryland School of Nursing, Baltimore, MD, USA.; b University of Washington Department of Global Health, Seattle, WA, USA.; c The George Institute for Global Health, Newtown, Australia.; d U.S. Agency for International Development, Washington, DC, USA.; e Social Solutions International, Inc., Washington, DC, USA.; f London School of Hygiene and Tropical Medicine, London, United Kingdom.; g World Health Organization, Geneva, Switzerland.

## Abstract

**Background:**

A critical shortage of health workers with needed maternal and newborn competencies remains a major challenge for the provision of quality care for mothers and newborns, particularly in low- and middle-income countries. Supply-side challenges related to human resources for health (HRH) worsen shortages and can negatively affect health worker performance and quality of care. This review scoped country-focused sources to identify and map evidence on HRH-related challenges to quality facility-based newborn care provision by nurses and midwives.

**Methods:**

Evidence for this review was collected iteratively, beginning with pertinent World Health Organization documents and extending to articles identified via database and manual reference searches and country reports. Evidence from country-focused sources from 2000 onward was extracted using a data extraction tool that was designed iteratively; thematic analysis was used to map the 10 categories of HRH challenges.

**Findings:**

A total of 332 peer-reviewed articles were screened, of which 22 met inclusion criteria. Fourteen additional sources were added from manual reference search and gray literature sources. Evidence has been mapped into 10 categories of HRH-related challenges: (1) lack of health worker data and monitoring; (2) poor health worker preservice education; (3) lack of HW access to evidence-based practice guidelines, continuing education, and continuing professional development; (4) insufficient and inequitable distribution of health workers and heavy workload; (5) poor retention, absenteeism, and rotation of experienced staff; (6) poor work environment, including low salary; (7) limited and poor supervision; (8) low morale, motivation, and attitude, and job dissatisfaction; (9) weaknesses of policy, regulations, management, leadership, governance, and funding; and (10) structural and contextual barriers.

**Conclusion:**

The mapping provides needed insight that informed new World Health Organization strategies and supporting efforts to address the challenges identified and strengthen human resources for neonatal care, with the ultimate goal of improving newborn care and outcomes.

## Abstract (precis)

We mapped evidence from low- and middle-income countries of the human resources for health-related challenges to providing quality facility-based newborn care into tangible thematic areas. The mapping provides valuable insight that informed new World Health Organization strategies to systematically address the challenges identified and to strengthen human resources for health for newborn care globally and nationally.

## Key Messages

- We mapped the evidence of human resources for health (HRH)-related challenges to providing quality facility-based newborn care into 10 categories:
  - Lack of HRH data and monitoring
  - Poor health worker (HW) preservice education
  - Lack of HW access to evidence-based guidelines, continuing education, and continuing professional development
  - Insufficient and inequitable distribution of HWs and heavy workload
  - Poor retention, absenteeism, and rotation of experienced staff
  - Poor work environment, including low salary
  - Limited and poor supervision
  - Low morale, motivation, and attitude, and job dissatisfaction
  - Weaknesses of policy, regulations, management, leadership, governance, and funding
  - Structural and contextual barriers
- Mapping the evidence provided useful insight to inform recently published World Health Organization strategies to systematically address the challenges and strengthen HRH for newborn care globally and nationally.
- The thematic analysis process also underscored the complicated interactions between different types of HRH challenges.
- Findings support new strategies for action to address these challenges.

Resumen en español al final del artículo. El texto completo del artículo también está disponible en español.

Le texte complet de l’article est aussi disponible en français.

## BACKGROUND

Newborns are extremely vulnerable; globally, about 2.5 million babies die during their first 28 days of life (the neonatal period), with about 77% of those deaths occurring during the first week of life.^1^^,^^2^ Additionally, almost 2 million stillbirths occur in the last 3 months of pregnancy or during childbirth each year,^3^^,^^4^ and millions of infants develop short- and long-term morbidities and neurocognitive problems.^5^^,^^6^ Most newborns can survive and thrive with access to quality health care, yet reductions in neonatal mortality remain slow and unequal due to variable coverage of essential interventions and quality care delivery by health workers (HWs).^7^^,^^8^ Universal access to quality care could prevent 1.7 million neonatal deaths each year or 68% of the deaths that will otherwise occur by 2030.^5^

Skilled birth attendants and midwives capable of providing high-quality childbirth, essential newborn, and referral-level care are critical because early neonatal deaths are inextricably linked to maternal health and to the quality of care a mother and her baby receive during labor, childbirth, and the immediate postpartum period.^7^ In addition, competent newborn workers—primarily composed of nurses and midwives with support from medical doctors and other health specialists—are needed to provide facility-based care to an estimated 30 million newborns every year who require care in a hospital setting.^5^^,^^9^ As the majority of women choose institutional delivery and neonatal mortality declines below 30/1,000 live births globally, interventions delivered in facilities across primary (basic), secondary (“special care”), and tertiary (neonatal intensive care) levels become increasingly important to achieve further declines.^10^ Facility-based maternal and newborn care refers to round-the-clock clinical services provided by skilled personnel at health care facilities, focused on routine care and management of complications.^11^^,^^12^ Together, nurses and midwives compose the largest percentage of HWs worldwide and are critical to achieving not only improved maternal and newborn health outcomes, but also stronger health systems that ensure all newborns not only survive but also thrive and realize their rights to the highest attainable standards of health and well-being.^5^^,^^13^^–^^15^

However, a critical shortage of HWs with needed maternal and neonatal competencies remains an impediment to scaling up the provision of skilled care for mothers and newborns, particularly in low- and middle-income countries (LMICs). Currently, high-income countries have on average 10.9 nurses and midwives per 1,000 population, compared with 2.5 and 0.9 in LMICs and low-income countries, respectively.^16^ Supply-side human resources for health (HRH)--related challenges worsen shortages and can negatively affect HW performance and quality of care.^17^^,^^18^ As the coronavirus disease (COVID-19) pandemic overburdens health systems in many countries, newborns—although less likely to die from COVID-19—are at increased risk for mortality from other preventable and treatable conditions as access to and availability of health services are disrupted.^19^

HWs with maternal and neonatal competencies are critically needed to scale up the provision of skilled care for mothers and newborns.

Despite increased attention to the issue of HRH since the 2006 World Health Report and World Health Assembly, and the creation of the Global Health Workforce Alliance, which spearheaded World Health Organization (WHO) HRH efforts from 2006 to 2016, specific attention to HRH for newborn care is more recent, as is a focus on the quality of care provided.^20^^,^^21^ It is now essential to address the critical shortage of competent HWs to attain the ambitious newly released Every Newborn Action Plan 2025 health targets and new WHO standards for improving the quality of care for small and sick newborns in health facilities, as part of progress toward attaining the Sustainable Development Goals (SDGs).^22^^,^^23^

This scoping review responds to the call for timely information for WHO's Year of the Nurse and Midwife to ensure that this relatively neglected topic has a place in the discussion. It scopes the literature to identify and map country-focused evidence on HRH-related challenges to quality facility-based newborn care provision by nurses and midwives in LMICs and provides the evidence base for recently published WHO strategies to address these challenges.^9^^,^^24^ While community-based care is also critical to reducing maternal and neonatal mortality and morbidity, this article focuses on newborn care provided in health care facilities.

## METHODS

### Approach

A scoping methodology was selected for this review because the approach allows for expeditious large-scale accumulation of literature and mapping of the evidence therein and determining the extent of the evidence and gaps requiring additional research.^25^ The approach applied in this scoping review uses a 5-stage process: (1) identifying the research question, (2) identifying relevant studies, (3) selecting studies, (4) charting the data, and (5) collating, summarizing, and reporting the results.^26^ Evidence for this review was collected iteratively, beginning with pertinent WHO documents and topical published series and extending to articles identified via database searches and manual reference searches, as well as country and organizational reports.

### Database Searches

This review investigated the following research question: what evidence is available on HRH-related challenges to provision of quality newborn care by nurses and midwives in LMICs. The database searches aimed to find peer-reviewed articles, commentaries, and reports from LMICs that addressed the topic of inquiry and were published starting in 2000, with the inception of the Millennium Development Goals.

This review investigated what evidence is available on HRH-related challenges to provision of quality newborn care by nurses and midwives in LMICs.

#### Search Strategy and Selection Criteria

Data for this review were identified by searches of PubMed, EMBASE, CENTRAL, Cumulative Index of Nursing and Allied Health Literature (CINAHL), African Journals Online (AJOL), Latin American & Caribbean Health Sciences Literature (LILACS), and references from relevant articles using the following search terms: nurses, nursing, midwives, midwifery, nurse-midwives, neonates, newborns, infants, premature, preterm, low birth weight, developing countries, low-income countries, middle-income countries, inpatient, hospitals, health care facilities, health centers, clinics, neonatal care units, newborn care units, neonatal intensive care units, health human resources, human resources for health, workforce, health personnel, policies, education, employment, deployment, distribution, retention, shortages, salaries, motivation, performance, supervision combined using Boolean operators AND and OR and limited to humans. Grey literature was sought through Open Grey (www.opengrey.eu), Grey Literature Report (www.greylit.org), and Healthy Newborn Network. Searches were conducted in English, without language restrictions. Only articles published from 2000 were included.

The complete search strategy is provided in a Supplement.

#### Inclusion and Exclusion Criteria

Search results were entered into Covidence software (www.covidence.org), and 2 reviewers (NEB and KDC) independently screened study titles and abstracts against inclusion and exclusion criteria. The articles selected for full-text review met inclusion criteria consistent with manuscripts examining HRH challenges for newborn care by nurses and midwives at the facility level in LMICs.

Specific inclusion criteria were:

1. Topic: addressed HRH challenge
2. Providers: nurses and/or midwives
3. Patient Population: from birth to 28 days of life (neonatal period) and caregiver
4. Setting: health care facilities at primary, secondary, or tertiary level in LMICs: country included on World Bank list for LMICs^27^
5. Country-focused; evidence from the national, regional, district, or facility-level in country
6. Years: 2000 to present

Reasons for exclusion were sources that were community-based only (not facility-based) or that described initiatives with traditional birth attendants, community HWs, or medical doctors only; policy initiatives that were theoretical but not implemented; or research carried out in refugee settings (since these settings often face unique challenges). Each reviewer independently screened each full-text document to determine whether the source should be included or excluded, and disagreements were resolved through discussion. The search and review results are shown (Figure).

**FIGURE:**
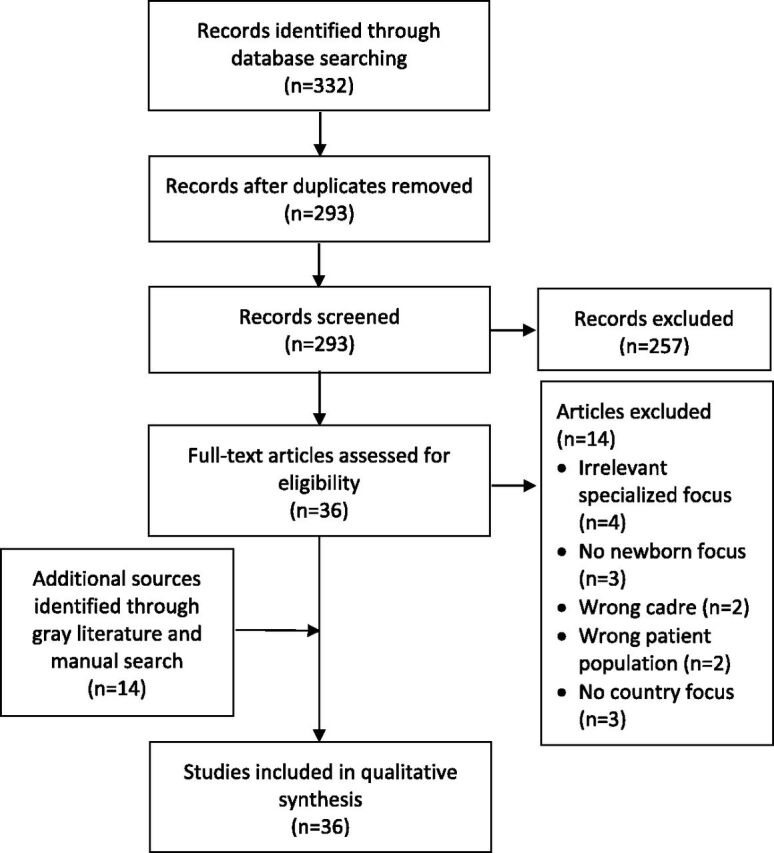
Flow Diagram of Search and Review Results of Evidence on Human Resources for Health-Related Challenges to Quality Newborn Care in Low- and Middle-Income Countries

### Data Extraction and Management

The primary author (NEB) read and coded each full-text document using a data extraction form that was created iteratively as we reviewed the full texts. Data fields were the following: HRH challenge, example (detail) of challenge, country, setting, research methods, type of document (peer-reviewed or gray), type of provider, year of publication, source, and any additional notes. The extracted data were used to create a concept map or chart of HRH challenges and then were grouped into categories by similar themes via inductive thematic analysis described by Braun and Clarke.^28^ Categories of challenges were reviewed by 2 authors (NEB and OL), collapsing the themes based on the volume of evidence in each category and using an iterative and inductive process to reach consensus on the final 10 categories of challenges.

## RESULTS

A total of 332 peer-reviewed articles, including 39 duplicates, were retrieved. The 293 abstracts were then reviewed using pre-identified inclusion and exclusion criteria. Of these sources, 22 met the inclusion criteria (Figure). Fourteen additional records were added from manual reference search and gray literature sources, bringing the total number of included articles to 36. Sources meeting the inclusion criteria covered 20 of 138 LMICs. With 8 peer-reviewed articles, India had the highest representation, and the remaining 19 countries were represented by 1 or 2 publications each. One publication covered 4 countries (Cambodia, Lao PDR, Vietnam, and Malaysia).^29^

Most sources included were peer-reviewed publications (n=33). Year of publication ranged from 2005 to 2019, and sources relied primarily on evidence about nurses (including obstetric and neonatal nurses, neonatal intensive care unit [NICU] nurses, and student nurses), midwives (including auxiliary nurse midwives), and to a lesser extent medical doctors, medical officers, district health officers, facility managers, and policy makers. Research methods utilized in the reviewed articles were both qualitative (interviews and focus groups) and quantitative (surveys, audits, multiple-choice and skills tests, questionnaires); articles also employed document review, record review, and observation to collect data. One article relied on data from a national multistakeholder group^30^ and another on secondary data analysis.^31^ Most studies reported on data from NICUs, health centers, hospitals, or district hospitals from specified regions. Four articles used national-level data for their analysis (Ethiopia, Indonesia, Ghana, and Uganda).^30^^,^^32^^–^^34^

The thematic analysis of the data resulted in 10 categories of HRH-related challenges faced by nurses and midwives in providing quality facility-based newborn care. A summary of data, classified by HRH challenge, is provided in the Table. Data from each source were often mapped to more than 1 challenge (Table).

**TABLE.** Mapped Human Resources for Health-Related Challenges With Review Sources

HRH Challenge	Examples	Country	Source Type	Source
1. Lack of data on HRH	Scarce HRH data as a barrier to HRH planning	Nigeria	PR	Adegoke et al.^35^
	Few workforce indicators for midwifery	Mongolia	PR	Kildea et al.^36^
2. Poor HW preservice education/insufficient newborn content	Lack of qualified instructors and clinical preceptors for midwives	Democratic Republic of the Congo	PR	Bogren et al.^37^
	Basic neonatal component in nursing preservice insufficient	Ghana	PR	Elikplim Pomevor and Adomah-Afari^38^
	Bachelor's-level nurse curriculum and performance poor compared to other providers (including diploma nurses)	Ethiopia	Report	Getachew et al.^32^
	HWs receive limited preservice instruction on neonatal care in their basic training	Kenya	PR	Aluvaala et al.^10^
	NB care not a core competency in general nursing education	India	PR	Campbell-Yeo et al.^39^
3. Lack of access for HWs to evidence-based practice guidelines and protocols, CE, and CPD	Nonavailability of ENC and NR guidelines/protocols	Ethiopia	PR	Haile-Mariam et al.^41^
	Lack of guidelines/posted protocols NR	Malawi	PR	Bream et al.^42^
	Few protocols for care of sick neonates available	Ghana	PR	Elikplim Pomevor and Adomah-Afari^38^
	Helping Babies Breathe guidelines in 25% of health facilities	Malawi	PR	Kozuki et al.^43^
	Nonavailability of NB protocols	Tanzania	PR	Nyamtema et al.^47^
	Poor dissemination of practice protocols to NICU nurses	Thailand	PR	Jirapaet et al.^44^
	Little in-service training on NB, NR, and small and sick newborn care	Ghana	PR	Elikplim Pomevor and Adomah-Afari^38^
		India	PR	Malhotra et al.^40^
	75% of health centers studied had no trained clinician in basic emergency obstetric and newborn care	Malawi	PR	Kozuki et al.^43^
	Variable CE opportunities for neonatal nurses	India	PR	Campbell-Yeo et al.^39^
	Limited access to CE for nurses and midwives	DR Congo	PR	Bolan et al.^66^
	Lack of policy on CE/renewal of skills	Malawi	PR	Bream et al.^42^
	Little CE for HWs on managing NR or newborn emergencies	The Gambia	PR	Cole-Ceesay et al.^45^
	No CE for midwives	Mongolia	PR	Kildea et al.^36^
	Lack of coordination of CPD of HWs, in past CPD not mandatory for MWs and absence of quality control over CPD	Liberia	PR	Michel-Schuldt et al.^46^
4. Insufficient and inequitable distribution of HWs, heavy workload	Insufficient HWs in general	Nigeria	PR	Adegoke et al.^35^
		Malawi	PR	Bream et al.^42^
		Tanzania	PR	Nyamtema et al.^47^
	Lack of HWs in primary health care/district hospitals	Nepal	PR	Allen and Jeffrey^54^
	Insufficient NICU staff	Thailand	PR	Jirapaet et al.^44^
	Lack of specialized nursing staff for neonatal care and sick newborns	SE Asia (4 countries)	PR	Martinez et al.^29^
		Solomon Islands	PR	Tosif et al.^55^
		India	PR	Neogi et al.^31^
	Acute nursing shortage in newborn units	Kenya	PR	Nzinga et al.^48^
		Kenya	PR	Aluvaala et al.^10^
	Shortage of HWs with neonatal training	Rwanda	PR	Ntigurirwa et al.^49^
	Low number of HWs compounded by uneven distribution.	Indonesia	Report	National Research Council^33^
	Poor distribution in rural/remote areas	Nepal	PR	Allen and Jeffrey^54^
		Nigeria	PR	Adegoke et al.^35^
		India	PR	Fischer et al.^50^
	Heavy workload for existing staff	India	PR	Amin et al.^51^
		Cambodia	PR	Ith et al.^52^
		India	PR	Morgan et al.^58^
	Gap filling with lower-level staff	Tanzania	PR	Prytherch et al.^53^
5. Poor retention, rotation out, absenteeism	Poor retention of HWs due to (examples) - Deaths of HWs from HIV - Rural-urban migration - External brain drain - Attrition to private sector and non-governmental organizations	Tanzania	PR	Nyamtema et al.^47^
		Rwanda	PR	Ntigurirwa et al.^49^
		India	PR	Neogi et al.^11^
		Gambia	PR	Cole-Ceesay et al.^45^
	Absenteeism	India	PR	Neogi et al.^11^
		Tanzania	PR	Nyamtema et al.^47^
		Tanzania	PR	Prytherch et al.^53^
	Rotation /transfer of neonatal nurses - To other units - To other facilities	India	PR	Dewez et al.^57^
		Ghana	PR	Elikplim Pomevor and Adomah-Afari^38^
		Rwanda	PR	Ntigurirwa et al.^49^
6. Poor work environment including: low salary; bad housing; lack of supplies, medications, equipment, electricity, and water; and poor safety and infrastructure	Low salary and irregular payment	Nigeria	PR	Adegoke et al.^35^
		Cambodia	PR	Ith et al.^52^
		Tanzania	PR	Prytherch et al.^53^
	Poor accommodations	Gambia	PR	Cole-Ceesay et al.^45^
	Lack of safety on the job - Lack of protective equipment - Fear theft, assaults, gender-based violence, assault by families	Nigeria	PR	Adegoke et al.^35^
		Gambia	PR	Cole-Ceesay et al.^45^
		India	PR	Morgan et al.^58^
		Tanzania	PR	Prytherch et al.^53^
	Lack of equipment, essential drugs, supplies	Nigeria	PR	Adegoke et al.^35^
		Southeast Asia (4 countries)	PR	Martinez et al.^29^
		Ethiopia	PR	Haile-Mariam et al.^41^
		India	PR	Morgan et al.^58^
	Lack of tech support, delays in repair	Solomon Islands	PR	Tosif et al.^55^
	Ward setup as barrier to care	India	PR	Neogi et al.^56^
		Malawi	PR	Bream et al.^42^
7. Limited and poor supervision	Limited capacity for routine supervision	Lao PDR	PR	Horiuchi et al.^59^
	No on-site mentors or technical support for MWs with limited skills	Nigeria	PR	Adegoke et al.^35^
	Lack of clinical supervision in NICU	Thailand	PR	Jirapaet et al.^12^
	Supervision punitive, no feedback given, lack of confidentiality	Tanzania	PR	Prytherch et al.^53^
	Problems remain unsolved after supervisory visits	Lao PDR	PR	Horiuchi et al.^59^
8. Low morale, poor motivation and attitude, dissatisfaction	Poor conditions, salaries, lack of incentives	Gambia	PR	Cole-Ceesay et al.^45^
	Lack of career ladder and promotion opportunities for HWs	Nigeria	PR	Adegoke et al.^35^
		Mongolia	PR	Kildea et al.^36^
		Tanzania	PR	Prytherch et al.^53^
	Burnout and stress, overwhelmed by workload and patient numbers	India	PR	Amin et al.^51^
		India	PR	Dewez et al.^57^
	Powerless over work environment, lack of control over transfers	Tanzania	PR	Prytherch et al.^53^
		India	PR	Dewez et al.^57^
	Low morale from high mortality of patients and inability to provide good care due to poor conditions	Gambia	PR	Cole-Ceesay et al.^45^
		India	PR	Dewez et al.^57^
		Tanzania	PR	Prytherch et al.^53^
9. Weaknesses of policy, regulations, management, leadership, governance, and funding	Lack of policy and regulatory frameworks for neonatal care and clinical protocols	Ghana	PR	Elikplim Pomevor and Adomah-Afari^38^
		Cambodia	PR	Ith et al.^52^
	Limited scope of practice for MWs, not in line with international scope of practice	Mongolia	PR	Kildea et al.^36^
	No nationally accepted competency standards for MWs and nurses	Mongolia	PR	Kildea et al.^36^
		Indonesia	Report	National Research Council^33^
	Lack of parity between public and faith-based workers' salaries	Tanzania	PR	Prytherch et al.^53^
	Absence of staffing norms by facility level	Ghana	Report	Ministry of Health Ghana^30^
	Nonrecognition by management of need for ICU-like ratios in neonatal care units	India	PR	Dewez et al.^57^
	Lack of support by hospital management team, facility leadership, or authorities	India	PR	Dewez et al.^57^
		India	PR	Fischer et al.^50^
	Lack of job descriptions, neonatal guidelines, or orientation in newborn units	Kenya	PR	Nzinga et al.^48^
		Tanzania	PR	Prytherch et al.^53^
		India	PR	Campbell-Yeo et al.^39^
	Low productivity by HWs	Tanzania	PR	Nyamtema et al.^47^
	Unsupportive management, inflexible work schedules	Tanzania	PR	Prytherch et al.^53^
	HR deficiencies at management level resulting in inefficient management, particularly related to budgeting	Lao PDR	PR	Sychareun et al.^60^
	Nonprioritization of newborn health by policy makers in past	Ghana	Report	Ministry of Health Ghana^30^
	Weak professional associations that do not improve conditions of HWs	Tanzania	PR	Prytherch et al.^53^
	Inadequate funding in national budgets for neonatal care (public sector)	Uganda	PR	Mbonye et al.^34^
		Tanzania	PR	Nyamtema et al.^47^
		Gambia	PR	Cole-Ceesay et al.^45^
10. Structural and contextual barriers	Lack of recognition of newborn specialty care requirements	India	PR	Campbell-Yeo et al.^39^
	Lack of community perception of neonatal disease burden	Nepal	PR	Allen and Jeffrey^54^
	Neglect of female newborns	India	PR	Morgan et al.^58^
	Child-bearing women lack power to determine care-seeking behavior in emergencies	Nepal	PR	Brunson^61^

Abbreviations: CE, continuing education; CPD, continuing professional development; ENC, essential newborn care; HRH, human resources for health; HW, health worker; ICU, intensive care unit; MW, midwife; NB, newborn; NICU, neonatal intensive care unit; NR, neonatal resuscitation; PR, peer-reviewed.

A thematic data analysis yielded 10 categories of HRH-related challenges faced by nurses and midwives in providing quality facility-based newborn care.

### Categories of HRH Challenges

#### 1. Lack of Data and Monitoring on HRH Required for Maternal and Newborn Health

Reviewed sources reported a lack of HRH data on personnel availability, distribution, trends, and requirements.^35^^,^^36^ For example, Nigeria has a critical shortage of HWs, particularly for health facilities in rural areas, and the problematic task of planning workforce hiring and distribution is rendered more difficult by lack of critical HRH data.^35^ Similarly, in Mongolia, Kildea et al.^36^ noted that in addition to an overall shortage of nurses, midwives, and allied health professionals for maternal and newborn care, it is difficult to determine the needed numbers of different HW cadres, linked to lack of metrics for measuring those cadres.

#### 2. Poor HW Preservice Education and Insufficient Newborn Content

Sources support that HW preservice training is often weak, particularly as it pertains to newborn knowledge and skills training across all cadres, and few programs exist for training specialized neonatal nurses in LMICs.^10^^,^^32^^,^^37^^–^^39^ For example, HWs in Kenya receive limited preservice instruction on neonatal care in their basic training, gaining most practical experience during clinical placements or internships in hospitals.^10^ Similarly, newborn care was observed not to be a core competency in general nursing education in India^39^; in Ghana, newborn content was noted to be insufficient in nursing preservice education.^38^

#### 3. Lack of Access for HWs to Evidence-Based Practice Guidelines and Protocols, Continuing Education, and Continuing Professional Development

Unavailability or lack of access to current evidence-based practice guidelines or protocols, continuing education, and continuing professional development for newborn care is a common complaint in the sources from LMICs.^36^^,^^38^^–^^47^ Jirapaet et al.^44^ reported on a study conducted in 4 NICUs in Thailand where researchers noted a lack of dissemination of practice protocols to nurses. Similarly, a study in Ethiopia in all hospitals and health centers with deliveries reported a lack of availability of guidelines and protocols on essential newborn care and neonatal resuscitation.^41^ Continuing education, defined as an ongoing process of learning, is a cornerstone of continued competence and is closely connected to the quality of care and patient safety.^21^ Continuing professional development refers to the process of tracking and documenting the skills, knowledge, and experience that the HW gains both formally and informally beyond any initial training. Michel-Schuldt et al.^46^ noted that continuing professional development is not mandatory for midwives in Liberia and, more generally, there is a lack of coordination of continuing professional development for health professionals as well as an absence of quality control over the training for midwives. In the Gambia, Cole-Ceesay et al.^45^ found little continuing education for HWs on managing neonatal resuscitation or newborn emergencies; Ghana reported limited in-service training on newborn care, neonatal resuscitation, or care of small and sick newborns.^38^

#### 4. Insufficient and Inequitable Distribution of HWs and Heavy Workload

Sources gave numerous examples of lack of sufficient and equitable distribution of HWs—specifically lack of skilled birth attendants and skilled neonatal nurses—with resulting heavy workloads for existing staff and unacceptable staffing ratios.^10^^,^^29^^,^^33^^,^^35^^,^^42^^,^^44^^,^^47^^–^^56^ A study in 2 newborn units in Nairobi public hospitals reported staffing ratios of 1 nurse to 15 babies.^48^ Aluvaala et al.^10^ found that of 22 hospital-based newborn units surveyed in Kenya, 6 had such severe personnel shortages that they were not able to allocate even 1 nurse specifically for each newborn unit. Similarly, a study across 9 NICUs in India reported inequitable staffing ratios that ranged from 1:4 to 1:8 in private facilities, but 1:25 to 1:35 in government facilities.^51^ A multi-country study in Southeast Asia reported that Hanoi neonatal units routinely cared for 50% more patients than allocated beds and staffing and that they were thus obliged to put 2 patients to a bed.^29^ Neogi et al.^56^ evaluated 8 special newborn care units in rural district hospitals throughout India and found that the nurse-to-newborn ratio appeared to play a critical role in improving newborn survival in these units: almost 15% of the variation in the neonatal mortality rate across the units could be explained by the nurse-to-newborn ratio. In addition to heavy workload for existing staff in rural facilities, Tanzania reported an imbalance between the proportion of skilled health staff and lower-level cadres in rural maternal-newborn workers, with 43% of the workforce made up of lower-level cadres (e.g., maternal and child health aides, assistant clinical officers, and attendants).^53^

#### 5. Poor Retention, Absenteeism, and Rotation of Experienced Staff

Poor retention of maternal and newborn workers was described in many sources.^11^^,^^38^^,^^45^^,^^47^^,^^49^^,^^53^^,^^57^ Absenteeism was reported^11^ due to HWs from the public sector also working in private practice in India. A study carried out in 2 rural health centers in Tanzania reported that HWs often have parallel income-generating activities, such as agriculture, commerce, or other health work, to complement their salaries, which can lead to distraction at work and absenteeism.^53^ Many articles included in this review also cited rotation within the facility or transfer to another facility as a major concern. In a study of 6 health facilities in Ghana, many HWs interviewed were concerned about yearly rotation of neonatal staff, as this hampered quality of care given the loss of experienced staff and the time required to get new staff up to speed.^38^ Dewez et al.^57^ described that when nurses trained in India in neonatal care and were rotated to other wards they lost confidence and neonatal skills.

#### 6. Poor Work Environment

Poor salaries, inconsistent payment, and substandard accommodations are often reported.^29^^,^^31^^,^^35^^,^^41^^,^^42^^,^^45^^,^^52^^,^^53^^,^^55^^,^^58^ A qualitative study carried out with HWs in Cambodia^52^ reported low salaries for skilled birth attendants, such that they only provide postnatal care to the mother and newborn if additional payments are made by family members. As noted in HRH challenge 5, HWs often create additional income-generating activities to supplement their incomes.^53^ Lack of equipment, supplies, medications, electricity, and water are often reported in the literature.^29^^,^^35^^,^^41^^,^^58^ A lack of supplies and protective equipment, such as gloves and masks, can affect HW safety. It can be exacerbated during outbreaks such as the current COVID-19 pandemic and contributes to poor quality of care.^19^ Additionally, lack of HW security due to blame and assault appears in the literature; an article from India noted that providers refer complicated cases out so as not to be assaulted by family members in the case of poor maternal and neonatal outcomes.^58^

#### 7. Limited and Poor Supervision

Poor supervision is cited as a problem leading to poor HW retention, low morale and motivation, and poor-quality care provision.^35^^,^^44^^,^^53^^,^^59^ Horiuchi et al.^59^ noted that although external supervisory visits are a common approach to promote behavioral change among newborn HWs, reinforce skills, and maintain quality of care, these visits are often not feasible in resource-limited settings, such as Lao PDR, due to both high cost and human resource demands. The authors note that there is minimal capacity to implement routine supervision; however, when supervision visits occur, problems identified often remain unsolved.^59^ Similarly, maternal and newborn HWs interviewed in Tanzania reported supervision and performance appraisal to be punitive and to lack confidentiality.^53^

#### 8. Low Morale, Motivation, and Attitude, and Job Dissatisfaction

Morale and motivation were frequently referenced in the sources.^35^^,^^36^^,^^45^^,^^51^^,^^53^^,^^57^ Low motivation and job dissatisfaction were linked to a variety of factors, including low salaries, poor working conditions, lack of career and promotion opportunities, lack of control over being transferred, insufficient training and technical guidance, burnout and stress linked to heavy workload, and demoralizing supervision.^36^^,^^45^^,^^51^^,^^53^ Several articles reported high stress and low morale due to high maternal and newborn mortality^45^^,^^51^ and HW guilt and feelings of powerlessness linked to not being able to provide better care to patients.^57^ Stress and burnout were reported among NICU workers in India and Thailand.^44^^,^^51^ Low morale and poor attitudes were also noted to affect care provision; for example, provider passivity in attending life-threatening emergencies in Tanzania was noted to contribute to poor outcomes.^47^

#### 9. Weaknesses of Policy, Regulations, Management, Leadership, Governance, and Funding

Sources documenting weaknesses in the policy and management arena were varied in nature.^30^^,^^33^^,^^34^^,^^36^^,^^38^^,^^39^^,^^47^^,^^48^^,^^50^^,^^52^^,^^53^^,^^57^^,^^60^ A study conducted in Tanzania concluded that factors discouraging maternal newborn providers could be divided into those that pertain to conditions of employment (e.g., related to policy), and those that pertain to the organization of work processes (e.g., related to facility management).^53^ Leadership, governance, and funding references pointed to a lack of prioritization of newborn care and lack of funding for it in national budgets.^34^

#### 10. Structural and Contextual Barriers

A variety of potential structural and contextual barriers to the provision of high-quality newborn care augment the aforementioned HRH challenges.^39^^,^^54^^,^^58^^,^^61^ A study in rural Nepal reported that a significant barrier to improving neonatal care was a lack of perception of the neonatal disease burden in the community from which health providers originate and that without the perception of a problem, providers have little incentive to improve job performance.^54^ A report from Bihar, India observed a preference for male children and neglect of female newborns, to the extent that families sometimes threaten HWs who attempt to resuscitate female newborns.^58^ Gender biases affect newborn care in a variety of additional ways—from power dynamics for majority female midwifery and nursing professions to gender-based power dynamics related to birthing decisions such as emergency care-seeking behavior—as reported in Nepal.^61^

## DISCUSSION

To our knowledge, this review is the first to comprehensively look at HRH challenges to providing quality newborn care in LMICs. In conducting this scoping review, we sought to better understand HRH-related challenges to quality facility-based neonatal care provision by nurses and midwives in LMICs by identifying tangible thematic areas to address at national, regional, and international levels. We aimed to synthesize evidence identified in country-focused sources and to provide needed insight to inform strategies for strengthening HRH for newborn care globally and nationally, reinforcing country capacity and capability to meet new WHO standards for the care of small and sick newborns, ENAP 2025 targets, and SDGs. The goals of addressing this pressing set of challenges are to improve quality care and to reduce mortality, stillbirths, and short-and-long term morbidity of newborns.

The goals of addressing HRH challenges are to improve quality care and to reduce mortality, stillbirths, and short-and-long term morbidity of newborns.

Weak HRH data and monitoring make workforce decision making and planning particularly difficult.^5^^,^^18^ Essential data on availability of HWs, especially nurses and midwives, are often missing.^36^ Data showing the necessary facility mix of staff skills are lacking, which adds to planning challenges.^7^ To improve quality care for newborns and to facilitate advocacy, data are needed on who, where, and how HWs care for newborns.^62^ Research on HRH metrics and monitoring frameworks to guide national planning is a global priority.^5^

Evidence from multiple countries shows that the preservice education of physicians, nurses, and midwives is often low; additionally, the rapid mushrooming of nonaccredited private sector schools raises questions about their quality.^63^ Neonatal care content is often limited in preservice curricula of relevant cadres.^64^ Bottleneck analysis for inpatient care of small and sick newborns revealed a dearth of skills-based training in top high-burden neonatal mortality countries, and indicated that related education is inconsistent and poorly structured.^12^^,^^17^ Part of the difficulty stems from lack of trained faculty and qualified clinical preceptors for teaching neonatal care, resulting in limited opportunities to acquire newborn care skills during the preservice period.^37^^,^^64^ The means of fast-tracking faculty training in neonatal content and assuring faculty retention is not addressed in the literature and is an area where research is needed.

Access to relevant, up-to-date guidelines, protocols, and continuing education and continuing professional development opportunities for nurses and midwives is often difficult or impossible, especially in rural areas.^21^^,^^65^^,^^66^ For care specific to small and sick babies, research in 12 countries with high neonatal mortality rates reported that there was inadequate or no competency-based training or continuing education, including in-service and refresher training, particularly at lower-level facilities.^12^ Guidelines change regularly and thus dissemination and methods of updating HWs on new knowledge are essential; however, simple dissemination of written guidelines is ineffective.^67^ Most obstetric and neonatal emergencies take place in peripheral health facilities, which are difficult to reach with conventional training programs and require innovative learning strategies.^68^ Opportunities to address these challenges are now available through evidence-based and competency-based learning packages that incorporate simulation training and mentoring, digital e- and m-learning initiatives, and other innovative learning tools that utilize technology such as virtual reality training tools, clinical decision, and point-of-care learning and support tools.^66^^,^^69^^,^^70^ However, additional research is needed on e- and m-learning, particularly in LMICs, that measures newborn outcomes as a primary outcome.^70^

Shortages of HWs (theme 4) is one of the main factors behind persistent high mortality rates for women and newborns in many countries.^5^^,^^71^ Fewer than 1 in 6 countries with the highest burden of maternal and neonatal mortality reaches the minimum benchmark necessary to provide a basic package of care, identified as 23 doctors, midwives, and nurses per 10,000 population.^9^ An insufficient number of HWs, combined with poor working conditions and few incentives for staff to live and work in remote areas or among disadvantaged populations, leads to unequal distribution. Imbalances exist not only in the number and geographical distribution of available HWs, but also in the employment sector (public/private)^72^ and in the range of HW skills. Most countries still have too few specialists relative to the health needs of their population; shortages are particularly evident for specialist doctors (e.g., neonatologists, surgeons, obstetricians, and anesthetists) and neonatal nurses, with few available programs for training these cadres in low-income settings.^64^^,^^71^ In contrast, in high-income countries neonatal nurses are the backbone of newborn facility-based care to the newborns and their families, including through extended roles such as advanced neonatal nurse practitioners.^12^^,^^73^

HW shortages and unequal distribution increase the workload of existing staff, leading to high staff-to-patient ratios and increased stress, impeding the ability to provide high quality care, and directly influencing patient outcomes.^31^^,^^40^ Research on NICU nurse staffing and workload in high-income countries showed that understaffing relative to national guidelines was associated with an increased risk for nosocomial infection in very low-birth-weight babies.^74^^,^^75^ In contrast, 1:1 NICU nursing staffing reduces in-hospital mortality.^76^^,^^77^ There are no globally accepted recommendations for staffing ratios at the different levels of newborn care provision, but we can contrast ratios in LMICs with recommended ratios in the United Kingdom of 1 specialized neonatal nurse to 1 patient for neonatal intensive care and 1 registered nurse or midwife to 4 patients for special care.^78^

Poor retention, absenteeism, and rotation of experienced nurses out of neonatal units can both create the dynamics above and contribute to their worsening (theme 5). The majority of countries (81%) show a workforce strongly favoring urban areas, which can be related to many factors, such as greater possibilities of private practice and unattractiveness of rural and remote areas due to poor working conditions, inadequate housing, limited opportunities for professional development, and limited educational opportunities for children.^72^^,^^79^

Additionally, even the best trained and motivated HW needs a supportive, enabling environment to work effectively, including well-maintained infrastructure and a reliable supply of medicines, supplies, and technologies (theme 6). Poor salaries and work environments contribute to other challenges, such as poor retention, especially in remote areas, and also to low morale and attitude.^17^ A systematic mapping of barriers to the provision of quality midwifery care reported routine absence of safe working conditions, such as availability of sharps disposal, water for handwashing, and basic protective supplies such as gloves.^80^ Similarly, a survey conducted in 364 health facilities throughout Africa, Asia, and Latin America found that essential supplies and equipment were widely unavailable and concluded that staff are often unable to perform key procedures, and that women, stillborn, and newborns die unnecessarily as a result.^81^

Supportive supervision (theme 7) is another tool that is used in most settings to support HWs and improve job performance. If done correctly, supervision can be a mechanism for providing professional development, improving job satisfaction, and increasing motivation.^67^ However, the reality is that supervisory visits often fall short of their goal. Supervisors may lack skills, tools, and transport to provide quality supervision.^17^^,^^67^ Planned studies to evaluate self-managed continuous monitoring by peer reviews and feedback sessions as a more sustainable way to improve quality of care may prove an alternative to current supervision approaches.^59^

Quality care provision is dependent on HW motivation—a critical driver for HWs' willingness to maintain their professional competence, apply themselves to their jobs, and continue in the workforce.^67^^,^^82^^–^^85^ Low levels of HW morale and high levels of stress and burnout have been identified in the literature as an often-neglected problem (theme 8) and have been widely documented among NICU nurses, nurses, and midwives.^51^^,^^80^^,^^86^ Researchers suggest that motivation can both influence performance directly and mediate the effect of other factors; thus, motivation—and interventions that improve job satisfaction (e.g., salaries, work conditions)—are likely to be important determinants of job performance and retention.^67^

Challenges related to weak or absent policies, regulations, management, leadership, governance, and funding (theme 9) are critical to quality newborn care provision.^30^^,^^33^^,^^34^^,^^36^^,^^38^^,^^39^^,^^47^^,^^48^^,^^50^^,^^52^^,^^53^^,^^57^^,^^60^ Examples include lack of alignment between national policy defining the legal scope of practice for various cadres and regulation of the cadres, job descriptions, preservice education, or actual practice.^72^ There is also a lack of regulation of private-sector educational institutions and health providers,^87^ and lack of needed policies, such as well-defined staff-to-patient ratios, referral systems, discharge criteria, and standardized levels of care.^12^ All health programs—whether funded by governments, development partners, civil society, or the private sector—must contribute to government-driven national priorities; achieving this goal requires improved governance and better coordination between national and subnational systems.^87^ National-level advocates are needed for advancement of high-quality care for newborns, including policymakers, key individuals within professional bodies, academics, and national institutions. In terms of health financing, lack of sustained, coordinated newborn funding remains a challenge,^34^ and mobilization of sufficient financing with better cash flow is needed.^87^

Finally, the literature describes structural and contextual barriers that exacerbate other HRH-related challenges, particularly the low social status of caring professions and gender inequality in a predominantly female workforce. For example, a mapping of barriers to the provision of quality midwifery care identified gender inequality and lack of female empowerment as the most significant barriers leading to stress and burnout, which in turn lead to disempowerment, diminishing self-esteem, and ultimately adoption of negative behaviors.^80^^,^^88^ Disempowerment has also been widely reported in nursing, a profession similarly dominated by women, along with high stress and burnout as noted above.^21^^,^^89^

Campbell^90^ wrote that the only route to achieve quality of care is “through the health worker,” and that effective universal coverage—with HRH ensuring both availability and quality coverage of needed health services—is the grand challenge for all countries. This finding was echoed by the Lancet Global Health Commission on High Quality Health Systems, which proposes a “reboot” of health systems given the extent of quality deficits.^20^ Competent human resources and necessary physical resources are needed at all times to avoid preventable mortality of women, stillbirths, and newborns.^81^ These principles, elevating the importance of the global health workforce, were articulated in the 2006 World Health Report and with the creation of the Global Health Workforce Alliance. Global strategies to address HRH challenges were reinforced by WHO in the Global Strategy on Human Resources for Health: Workforce 2030.^69^ Given new 2025 newborn health targets and standards for the care of small and sick, newborn-specific HRH strategies, informed by the challenges identified in this article, have recently been published by WHO to address the accessibility and quality of care of this most-vulnerable population.^9^ Challenges to the provision of high-quality care to mothers and newborns in countries are complex, interrelated, and intertwined with broader social and structural challenges and will require ongoing attention and prioritization at all levels of the health system if we are to succeed in reaching the SDGs by 2030.

### Limitations

Narrowing and focusing the research question was a challenge and the broad definition of newborn care resulted in a large amount of information. While an iterative, inductive thematic analysis process was used to identify HRH-related challenges to delivering quality newborn care, some overlaps existed in conceptual areas, which may have masked some of the importance, nuance, or interconnectedness of the categories of challenges. Additionally, our analysis is not representative of all LMICs, given that many countries do not have data on their respective HRH-related challenges, which may be unique and context specific.

## CONCLUSIONS

With only 10 years to reach the SDGs, it is critical to ensure access to quality care for all newborns in need of facility-based care. However, lack of a sufficient number of HWs with neonatal care competencies is a critical gap. This review scoped country-focused articles to explore HRH-related challenges to quality facility-based neonatal care provision by nurses and midwives in LMICs. The review identified and mapped evidence into 10 HRH-related challenges and interpreted the data. The mapping provides needed insight informing new WHO strategies and supporting efforts to address the challenges identified and strengthen human resources for neonatal care, with the ultimate goal of improving newborn care and outcomes.

## Supplementary Material

20-00362-Bolan-Supplement.pdf

GHSP2000362_French.pdf

GHSP2000362_Spanish.pdf

## Translation

**En español**

**Desafíos de los recursos humanos en el área de salud para garantizar atención neonatal de calidad por parte en países con ingresos bajos y medianos: una revisión exploratoria**

**Mensajes clave**

- Se mapeó evidencia de los desafíos de los recursos humanos en el área de la salud (RHS) y el poder brindar atención neonatal de calidad en centros hospitalarios. Esta evidencia se clasificó en 10 categorías:
  ∘ Falta de seguimiento y datos de los RHS
  ∘ Formación deficiente previa al ejercicio de las funciones de los trabajadores de la salud y escaso material de contenido acerca de la salud del recién nacido
  ∘ Falta de acceso a material dirigido a trabajadores de la salud, como guías de práctica clínica basadas en evidencia, educación continua y desarrollo profesional continuo
  ∘ Distribución insuficiente e inequitativa del personal de salud y cargas de trabajo extenuantes
  ∘ Mala retención del personal, ausentismo y rotación del personal experimentado
  ∘ Mal ambiente laboral, así como salarios bajos
  ∘ Supervisión limitada y deficiente
  ∘ Baja moral, falta de motivación y de actitud, así como insatisfacción laboral
  ∘ Debilidades en políticas, regulaciones, administración, liderazgo, gobernanza y financiamiento
  ∘ Barreras estructurales y contextuales
- El mapeo de la evidencia proporcionó información útil para poder presentar las estrategias de la Organización Mundial de la Salud publicadas recientemente, las cuales describen el abordaje sistemático de los desafíos y el fortalecimiento de los RHS para la atención del recién nacido, tanto a nivel mundial como a nivel nacional.
- El proceso de análisis temático también resalta las complicadas interacciones entre los diferentes tipos de desafíos de los RHS.
- Los hallazgos sugieren nuevas estrategias de acción para abordar estos desafíos.

**RESUMEN**

**Antecedentes:** La escasez crítica de trabajadores de la salud que a su vez cuenten con las competencias necesarias para atender a la madre y al recién nacido sigue siendo un desafío importante en la prestación de servicios de atención de calidad, especialmente, en países con ingresos bajos y medianos. La problemática de la oferta de los recursos humanos para la salud (RHS), agrava la falta de estos y puede afectar negativamente el desempeño de los trabajadores de la salud y la calidad de la atención. Esta investigación utiliza fuentes centralizadas en cada país para identificar y mapear la evidencia de los desafíos del campo de los RHS para la prestación de atención neonatal de calidad en centros hospitalarios por parte de enfermeras y parteras.

**Métodos:** La evidencia de esta revisión fue recopilada de forma iterativa, comenzando con documentos pertinentes de la Organización Mundial de la Salud y extendiéndose a los artículos identificados a través de bases de datos y búsquedas manuales en listas de referencias e informes de los países incluidos en el estudio. La evidencia se obtiene de fuentes enfocadas en cada país y se considera la información a partir del año 2000. La extracción de datos fue realizada utilizando una herramienta diseñada de forma reiterativa; además, se hizo uso de un análisis temático para mapear las 10 categorías de los desafíos de los RHS.

**Resultados:** Se examinaron un total de 332 artículos y fueron revisados por pares, 22 de estos cumplieron los criterios de inclusión. Se agregaron catorce fuentes adicionales de búsqueda manual de listas de referencias y fuentes de literatura de carácter no científico. La evidencia se ha mapeado en 10 categorías de desafíos relacionados con los RHS: (1) falta de datos y monitoreo de los trabajadores de la salud; (2) deficiencia en educación previa al servicio como trabajador de la salud; (3)falta de acceso a material dirigido a trabajadores de la salud, como guías de práctica clínica basadas en evidencia, educación continua y desarrollo profesional continuo; (4) distribución insuficiente y desigual de los trabajadores de la salud y extenuantes cargas de trabajo; (5) mala retención del personal, ausentismo y rotación del personal experimentado; (6) mal ambiente laboral, así como bajos salarios; (7) supervisión limitada y deficiente; (8) baja moral, falta de motivación y de actitud, así como insatisfacción laboral; (9) debilidades en políticas, regulaciones, administración, liderazgo, gobierno y financiamiento; y (10) barreras estructurales y contextuales.

**Conclusión:** El mapeo proporciona la información necesaria y muestra las nuevas estrategias de la Organización Mundial de la Salud y los esfuerzos para apoyar el abordaje de los desafíos identificados y fortalecer los recursos humanos para la atención neonatal, con el objetivo final de mejorar la atención y los resultados en el recién nacido.

**Peer Reviewed**

**First published online:** February 25, 2021.
