# Supplementary material for: Human Resources for Health-Related Challenges to Ensuring Quality Newborn Care in Low- and Middle-Income Countries: A Scoping Review
Source: Glob Health Sci Pract. 2021 Mar 31;9(1):160–76. doi: 10.9745/GHSP-D-20-00362 (PMC8087437; doi:10.9745/GHSP-D-20-00362)
Supplement: 20-00362-Bolan-Supplement.pdf [file 20-00362-Bolan-Supplement.pdf]

Supplement to: Bolan N, Cowgill KD, Walker K, et al. Human resources for health-related challenges to ensuring quality newborn care by nurses and midwives in low- and middle-income countries: a scoping review. *Glob Health Sci Pract*. 2021;9(1). https://doi.org/10.9745/GHSP-D-20-00362

Search strategies run by Emilie Ludeman, MSLIS

Total references on 02/04/2020: 332

Total references after de-duplication: 293

Save in Covidence

Limit publication date to 01/01/2000 – current

Limit to human studies

PubMed – 140 references on 02/04/2020

(nurse[tiab] OR nurses[tiab] OR nursing[tiab] OR midwife*[tiab] OR midwives[tiab] OR "Nurses"[mesh] OR "Nursing"[mesh] OR "Nurse Midwives"[mesh] OR "Midwifery"[mesh]) **AND**

(hospital*[tiab] OR newborn care unit*[tiab] OR SNCU[tiab] OR health care facilit*[tiab] OR NICU[tiab] OR neonatal intensive care unit*[tiab] OR health center*[tiab] OR clinic[tiab] OR clinics[tiab] OR "Hospitals"[mesh] OR "Intensive Care, Neonatal"[mesh]) **AND**

(neonat*[tiab] OR newborn*[tiab] OR preterm[tiab] OR low birth weight[tiab] OR infant*[tiab] OR premature[tiab] OR small for gestational age[tiab] OR "Infant, Newborn"[mesh] OR "Infant, Low Birth Weight"[mesh] OR "Infant, Very Low Birth Weight"[mesh] OR "Infant, Premature"[mesh]) **AND**

(educat*[tiab] OR employ*[tiab] OR deploy*[tiab] OR distribut*[tiab] OR retention[tiab] OR retain*[tiab] OR shortage*[tiab] OR salary[tiab] OR salaries[tiab] OR motivation[tiab] OR performance[tiab] OR supervis*[tiab] OR policy[tiab] OR policies[tiab]) **AND**

(health human resources[tiab] OR human resources for health[tiab] OR health worker data[tiab] OR “Workforce”[MeSH] OR "Health Personnel"[MeSH]) **AND**

(developing country[tiab] OR developing countries[tiab] OR “Developing Countries”[Mesh] OR low income country[tiab] OR middle income country[tiab] OR low income countries[tiab] OR middle income countries[tiab] OR Afghanistan[tiab] OR Gambia[tiab] OR Nepal[tiab] OR Bangladesh[tiab] OR Guinea[tiab] OR Niger[tiab] OR Benin[tiab] OR Guinea-Bissau[tiab] OR Rwanda[tiab] OR Burkina Faso[tiab] OR Haiti[tiab] OR Sierra Leone[tiab] OR Burundi[tiab] OR Kenya[tiab] OR Somalia[tiab] OR Cambodia[tiab] OR Korea[tiab] OR Tajikistan[tiab] OR Central African Republic[tiab] OR Liberia[tiab] OR Tanzania[tiab] OR Chad[tiab] OR Madagascar[tiab] OR Togo[tiab] OR Comoros[tiab] OR Malawi[tiab] OR Uganda[tiab] OR Congo[tiab] OR Mali[tiab] OR Zimbabwe[tiab] OR Eritrea[tiab] OR Mozambique[tiab] OR Ethiopia[tiab] OR Myanmar[tiab] OR Armenia[tiab] OR Kiribati[tiab] OR (São Tomé and Principe[tiab]) OR Bhutan[tiab] OR Kosovo[tiab] OR Senegal[tiab] OR Bolivia[tiab] OR Kyrgyz Republic[tiab] OR Solomon Islands[tiab] OR Cameroon[tiab] OR Lao PDR[tiab] OR Laos[tiab] OR South Sudan[tiab] OR Cabo Verde[tiab] OR Lesotho[tiab] OR Sri Lanka[tiab] OR Mauritania[tiab] OR Sudan[tiab] OR Côte d'Ivoire[tiab] OR Micronesia[tiab] OR Swaziland[tiab] OR Djibouti[tiab] OR Moldova[tiab] OR Syrian Arab Republic[tiab] OR Syria[tiab] OR Egypt[tiab] OR Mongolia[tiab] OR Timor-Leste[tiab] OR El Salvador[tiab] OR Morocco[tiab] OR Ukraine[tiab] OR Georgia[tiab] OR Nicaragua[tiab] OR Uzbekistan[tiab] OR Ghana[tiab] OR Nigeria[tiab] OR Vanuatu[tiab] OR Guatemala[tiab] OR Pakistan[tiab] OR Vietnam[tiab] OR Guyana[tiab] OR Papua New Guinea[tiab] OR West Bank and Gaza[tiab] OR Gaza[tiab] OR Honduras[tiab] OR Paraguay[tiab] OR Yemen[tiab] OR Indonesia[tiab] OR Philippines[tiab] OR Zambia[tiab] OR India[tiab] OR Samoa[tiab] OR Angola[tiab] OR Fiji[tiab] OR Palau[tiab] OR Albania[tiab] OR Gabon[tiab] OR Panama[tiab] OR Algeria[tiab] OR Grenada[tiab] OR Peru[tiab] OR American Samoa[tiab] OR Hungary[tiab] OR Romania[tiab] OR Argentina[tiab] OR Iran[tiab] OR Serbia[tiab] OR Azerbaijan[tiab] OR Iraq[tiab] OR Seychelles[tiab] OR Belarus[tiab] OR Jamaica[tiab] OR South Africa[tiab] OR Belize[tiab] OR Jordan[tiab] OR St. Lucia[tiab] OR (Bosnia and Herzegovina[tiab]) OR Bosnia[tiab] OR Kazakhstan[tiab] OR (St. Vincent and the

Grenadines[tiab]) OR Botswana[tiab] OR Lebanon[tiab] OR Suriname[tiab] OR Brazil[tiab] OR Libya[tiab] OR Thailand[tiab] OR Bulgaria[tiab] OR Macedonia[tiab] OR Tonga[tiab] OR China[tiab] OR Malaysia[tiab] OR Tunisia[tiab] OR Colombia[tiab] OR Maldives[tiab] OR Turkey[tiab] OR Costa Rica[tiab] OR Marshall Islands[tiab] OR Turkmenistan[tiab] OR Cuba[tiab] OR Mauritius[tiab] OR Tuvalu[tiab] OR Dominica[tiab] OR Mexico[tiab] OR Venezuela[tiab] OR Dominican Republic[tiab] OR Montenegro[tiab] OR Ecuador[tiab] OR Namibia[tiab]) **AND**

("Humans"[Mesh])

Embase – 123 references on 02/04/2020

(neonat*:ab,ti OR newborn*:ab,ti OR infant*:ab,ti OR preterm:ab,ti OR 'low birth weight':ab,ti OR premature:ab,ti OR 'small for gestational age':ab,ti OR 'newborn'/de OR 'low birth weight'/exp OR 'prematurity'/de) **AND**

(nurse*:ab,ti OR midwife*:ab,ti OR midwives:ab,ti OR 'nurse'/exp OR 'nurse midwife'/exp) AND

(hospital*:ab,ti OR 'newborn care unit*':ab,ti OR SNCU:ab,ti OR 'health care facilit*':ab,ti OR NICU:ab,ti OR 'neonatal intensive care unit*':ab,ti OR 'health center*':ab,ti OR clinic:ab,ti OR clinics:ab,ti OR 'hospital'/exp OR 'neonatal intensive care unit'/de OR 'health center'/de) **AND**

(educat*:ab,ti OR employ*:ab,ti OR deploy*:ab,ti OR distribut*:ab,ti OR retention:ab,ti OR retain*:ab,ti OR shortage*:ab,ti OR salary:ab,ti OR salaries:ab,ti OR motivation:ab,ti OR performance:ab,ti OR supervis*:ab,ti OR policy:ab,ti OR policies:ab,ti) **AND**

('health human resources':ab,ti OR 'human resources for health':ab,ti OR 'health worker data':ab,ti OR 'workforce'/exp OR 'health care personnel'/de) **AND**

('low and middle income countr*':ab,ti OR 'low income countr*':ab,ti OR 'middle income countr*':ab,ti OR 'developing countr*':de,ab,ti OR afghanistan:ab,ti OR gambia:ab,ti OR Nepal;ab,ti OR Bangladesh:ab,ti OR guinea:ab,ti OR niger:ab,ti OR benin:ab,ti OR 'guinea bissau':ab,ti OR Rwanda:ab,ti OR 'burkina faso':ab,ti OR Haiti:ab,ti OR 'sierra leone':ab,ti OR Burundi:ab,ti OR kenya:ab,ti OR Somalia:ab,ti OR Cambodia:ab,ti OR korea:ab,ti OR Tajikistan:ab,ti OR 'central african republic':ab,ti OR Liberia:ab,ti OR Tanzania:ab,ti OR chad:ab,ti OR Madagascar:ab,ti OR togo:ab,ti OR comoros:ab,ti OR Malawi:ab,ti OR Uganda:ab,ti OR congo:ab,ti OR mali:ab,ti OR Zimbabwe:ab,ti OR Eritrea:ab,ti OR Mozambique:ab,ti OR Ethiopia:ab,ti OR Myanmar:ab,ti OR Armenia:ab,ti OR Kiribati:ab,ti OR 'são tomé and principe':ab,ti OR Bhutan:ab,ti OR Kosovo:ab,ti OR Senegal:ab,ti OR Bolivia:ab,ti OR 'kyrgyz republic':ab,ti OR 'solomon islands':ab,ti OR Cameroon:ab,ti OR 'lao pdr':ab,ti OR laos:ab,ti OR 'south sudan':ab,ti OR 'cabo verde':ab,ti OR Lesotho:ab,ti OR 'sri lanka':ab,ti OR Mauritania:ab,ti OR sudan:ab,ti OR 'côte divoire':ab,ti OR Micronesia:ab,ti OR Swaziland:ab,ti OR Djibouti:ab,ti OR moldova:ab,ti OR 'syrian arab republic':ab,ti OR Syria:ab,ti OR Egypt:ab,ti OR Mongolia:ab,ti OR 'timor leste':ab,ti OR 'el salvador':ab,ti OR morocco:ab,ti OR Ukraine:ab,ti OR Georgia:ab,ti OR Nicaragua:ab,ti OR Uzbekistan:ab,ti OR Ghana:ab,ti OR Nigeria:ab,ti OR Vanuatu:ab,ti OR Guatemala:ab,ti OR Pakistan:ab,ti OR Vietnam:ab,ti OR Guyana:ab,ti OR 'papua new guinea':ab,ti OR 'west bank and gaza':ab,ti OR gaza:ab,ti OR Honduras:ab,ti OR Paraguay:ab,ti OR yemen:ab,ti OR Indonesia:ab,ti OR Philippines:ab,ti OR Zambia:ab,ti OR india:ab,ti OR samoa:ab,ti OR angola:ab,ti OR Fiji:ab,ti OR palau:ab,ti OR Albania:ab,ti OR gabon:ab,ti OR panama:ab,ti OR Algeria:ab,ti OR grenada:ab,ti OR peru:ab,ti OR 'american samoa':ab,ti OR hungary:ab,ti OR Romania:ab,ti OR argentina:ab,ti OR iran:ab,ti OR Serbia:ab,ti OR Azerbaijan:ab,ti OR Iraq:ab,ti OR Seychelles:ab,ti OR Belarus:ab,ti OR Jamaica:ab,ti OR 'south africa':ab,ti OR belize:ab,ti OR Jordan:ab,ti OR 'st. lucia':ab,ti OR 'bosnia and herzegovina':ab,ti OR bosnia:ab,ti OR Kazakhstan:ab,ti OR 'st. vincent and the grenadines':ab,ti OR Botswana:ab,ti OR Lebanon:ab,ti OR suriname:ab,ti OR brazil:ab,ti OR Libya:ab,ti OR Thailand:ab,ti OR Bulgaria:ab,ti OR Macedonia:ab,ti OR tonga:ab,ti OR china:ab,ti OR Malaysia:ab,ti OR Tunisia:ab,ti OR Colombia:ab,ti OR Maldives:ab,ti OR turkey:ab,ti OR 'costa rica':ab,ti OR 'marshall islands':ab,ti OR Turkmenistan:ab,ti OR cuba:ab,ti

OR Mauritius:ab,ti OR Tuvalu:ab,ti OR dominica:ab,ti OR mexico:ab,ti OR Venezuela:ab,ti OR 'dominican republic':ab,ti OR Montenegro:ab,ti OR Ecuador:ab,ti OR Namibia:ab,ti) **AND**

[humans]/lim

CENTRAL 13 on 02/04/2020

(newborn* OR neonat* OR infant* OR premature OR preterm OR "low birth weight" OR "small for gestational age") **AND**

(nurs* OR midwife* OR midwives) **AND**

(hospital* OR NICU OR "neonatal intensive care unit*" OR "newborn care unit" OR SNCU OR "health facilit*" OR clinic OR clinics OR "health center*") **AND**

(educat* OR employ* OR deploy* OR distribut* OR retention OR retain* OR shortage* OR salary OR salaries OR motivation OR performance OR supervis* OR policy OR policies) **AND**

("health human resources" OR "human resources for health" OR "health worker data" OR workforce OR "health care personnel") **AND**

("low and middle income countr*" OR low income countr* OR middle income countr* OR developing countr* OR Afghanistan OR Gambia OR Nepal OR Bangladesh OR Guinea OR Niger OR Benin OR Guinea-Bissau OR Rwanda OR "Burkina Faso" OR Haiti OR "Sierra Leone" OR Burundi OR Kenya OR Somalia OR Cambodia OR Korea OR Tajikistan OR "Central African Republic" OR Liberia OR Tanzania OR Chad OR Madagascar OR Togo OR Comoros OR Malawi OR Uganda OR Congo OR Mali OR Zimbabwe OR Eritrea OR Mozambique OR Ethiopia OR Myanmar OR Armenia OR Kiribati OR "São Tomé and Principe" OR Bhutan OR Kosovo OR Senegal OR Bolivia OR "Kyrgyz Republic" OR "Solomon Islands" OR Cameroon OR "Lao PDR" OR Laos OR "South Sudan" OR "Cabo Verde" OR Lesotho OR "Sri Lanka" OR Mauritania OR Sudan OR "Côte d'Ivoire" OR Micronesia OR Swaziland OR Djibouti OR Moldova OR "Syrian Arab Republic" OR Syria OR Egypt OR Mongolia OR Timor-Leste OR "El Salvador" OR Morocco OR Ukraine OR Georgia OR Nicaragua OR Uzbekistan OR Ghana OR Nigeria OR Vanuatu OR Guatemala OR Pakistan OR Vietnam OR Guyana OR "Papua New Guinea" OR "West Bank and Gaza" OR Gaza OR Honduras OR Paraguay OR Yemen OR Indonesia OR Philippines OR Zambia OR India OR Samoa OR Angola OR Fiji OR Palau OR Albania OR Gabon OR Panama OR Algeria OR Grenada OR Peru OR "American Samoa" OR Hungary OR Romania OR Argentina OR Iran OR Serbia OR Azerbaijan OR Iraq OR Seychelles OR Belarus OR Jamaica OR "South Africa" OR Belize OR Jordan OR "St. Lucia" OR "Bosnia and Herzegovina" OR Bosnia OR Kazakhstan OR "St. Vincent and the Grenadines" OR Botswana OR Lebanon OR Suriname OR Brazil OR Libya OR Thailand OR Bulgaria OR Macedonia OR Tonga OR China OR Malaysia OR Tunisia OR Colombia OR Maldives OR Turkey OR "Costa Rica" OR "Marshall Islands" OR Turkmenistan OR Cuba OR Mauritius OR Tuvalu OR Dominica OR Mexico OR Venezuela OR "Dominican Republic" OR Montenegro OR Ecuador OR Namibia)

CINAHL 16 on 02/04/2020

(newborn* OR neonat* OR infant* OR premature OR preterm OR "low birth weight" OR "small for gestational age") **AND**

(nurs* OR midwife* OR midwives) **AND**

(hospital* OR NICU OR "neonatal intensive care unit*" OR "newborn care unit" OR SNCU OR "health facilit*" OR clinic OR clinics OR "health center*") **AND**

(educat* OR employ* OR deploy* OR distribut* OR retention OR retain* OR shortage* OR salary OR salaries OR motivation OR performance OR supervis* OR policy OR policies) **AND**

Supplement to: Bolan N, Cowgill KD, Walker K, et al. Human resources for health-related challenges to ensuring quality newborn care by nurses and midwives in low- and middle-income countries: a scoping review. *Glob Health Sci Pract*. 2021;9(1). https://doi.org/10.9745/GHSP-D-20-00362

(“health human resources” OR “human resources for health” OR “health worker data” OR workforce OR “health care personnel”) **AND**

("low and middle income countr*" OR low income countr* OR middle income countr* OR developing countr* OR Afghanistan OR Gambia OR Nepal OR Bangladesh OR Guinea OR Niger OR Benin OR Guinea-Bissau OR Rwanda OR "Burkina Faso" OR Haiti OR "Sierra Leone" OR Burundi OR Kenya OR Somalia OR Cambodia OR Korea OR Tajikistan OR "Central African Republic" OR Liberia OR Tanzania OR Chad OR Madagascar OR Togo OR Comoros OR Malawi OR Uganda OR Congo OR Mali OR Zimbabwe OR Eritrea OR Mozambique OR Ethiopia OR Myanmar OR Armenia OR Kiribati OR "São Tomé and Principe" OR Bhutan OR Kosovo OR Senegal OR Bolivia OR "Kyrgyz Republic" OR "Solomon Islands" OR Cameroon OR "Lao PDR" OR Laos OR "South Sudan" OR "Cabo Verde" OR Lesotho OR "Sri Lanka" OR Mauritania OR Sudan OR "Côte d'Ivoire" OR Micronesia OR Swaziland OR Djibouti OR Moldova OR "Syrian Arab Republic" OR Syria OR Egypt OR Mongolia OR Timor-Leste OR "El Salvador" OR Morocco OR Ukraine OR Georgia OR Nicaragua OR Uzbekistan OR Ghana OR Nigeria OR Vanuatu OR Guatemala OR Pakistan OR Vietnam OR Guyana OR "Papua New Guinea" OR "West Bank and Gaza" OR Gaza OR Honduras OR Paraguay OR Yemen OR Indonesia OR Philippines OR Zambia OR India OR Samoa OR Angola OR Fiji OR Palau OR Albania OR Gabon OR Panama OR Algeria OR Grenada OR Peru OR "American Samoa" OR Hungary OR Romania OR Argentina OR Iran OR Serbia OR Azerbaijan OR Iraq OR Seychelles OR Belarus OR Jamaica OR "South Africa" OR Belize OR Jordan OR "St. Lucia" OR "Bosnia and Herzegovina" OR Bosnia OR Kazakhstan OR "St. Vincent and the Grenadines" OR Botswana OR Lebanon OR Suriname OR Brazil OR Libya OR Thailand OR Bulgaria OR Macedonia OR Tonga OR China OR Malaysia OR Tunisia OR Colombia OR Maldives OR Turkey OR "Costa Rica" OR "Marshall Islands" OR Turkmenistan OR Cuba OR Mauritius OR Tuvalu OR Dominica OR Mexico OR Venezuela OR "Dominican Republic" OR Montenegro OR Ecuador OR Namibia)

AJOL (African Journals Online) 29 on 02/04/2020

https://www.ajol.info/index.php/index/search/search

(newborn* OR neonat* OR infant* OR preterm OR premature) AND (nurs* OR midwife* OR midwives)

LILACS 11 on 02/04/2020

(newborn* OR neonat* OR infant* OR premature OR preterm OR "low birth weight" OR "small for gestational age") **AND**

(nurs* OR midwife* OR midwives) **AND**

(hospital* OR NICU OR "neonatal intensive care unit*" OR "newborn care unit" OR SNCU OR "health facilit*" OR clinic OR clinics OR "health center*") **AND**

(educat* OR employ* OR deploy* OR distribut* OR retention OR retain* OR shortage* OR salary OR salaries OR motivation OR performance OR supervis* OR policy OR policies) **AND**

(“health human resources” OR “human resources for health” OR “health worker data” OR workforce OR “health care personnel”) **AND**

("low and middle income countr*" OR low income countr* OR middle income countr* OR developing countr* OR Afghanistan OR Gambia OR Nepal OR Bangladesh OR Guinea OR Niger OR Benin OR Guinea-Bissau OR Rwanda OR "Burkina Faso" OR Haiti OR "Sierra Leone" OR Burundi OR Kenya OR Somalia OR Cambodia OR Korea OR Tajikistan OR "Central African Republic" OR Liberia OR Tanzania OR Chad OR Madagascar OR Togo OR Comoros OR Malawi OR Uganda OR Congo OR Mali OR Zimbabwe OR Eritrea OR Mozambique OR Ethiopia OR Myanmar OR Armenia OR Kiribati OR "São Tomé and Principe" OR Bhutan OR Kosovo OR Senegal OR Bolivia OR "Kyrgyz Republic" OR "Solomon Islands" OR Cameroon OR "Lao PDR" OR Laos OR "South Sudan" OR "Cabo Verde" OR Lesotho OR "Sri Lanka" OR Mauritania OR Sudan OR "Côte d'Ivoire" OR

Supplement to: Bolan N, Cowgill KD, Walker K, et al. Human resources for health-related challenges to ensuring quality newborn care by nurses and midwives in low- and middle-income countries: a scoping review. *Glob Health Sci Pract*. 2021;9(1). https://doi.org/10.9745/GHSP-D-20-00362

Micronesia OR Swaziland OR Djibouti OR Moldova OR "Syrian Arab Republic" OR Syria OR Egypt OR Mongolia OR Timor-Leste OR "El Salvador" OR Morocco OR Ukraine OR Georgia OR Nicaragua OR Uzbekistan OR Ghana OR Nigeria OR Vanuatu OR Guatemala OR Pakistan OR Vietnam OR Guyana OR "Papua New Guinea" OR "West Bank and Gaza" OR Gaza OR Honduras OR Paraguay OR Yemen OR Indonesia OR Philippines OR Zambia OR India OR Samoa OR Angola OR Fiji OR Palau OR Albania OR Gabon OR Panama OR Algeria OR Grenada OR Peru OR "American Samoa" OR Hungary OR Romania OR Argentina OR Iran OR Serbia OR Azerbaijan OR Iraq OR Seychelles OR Belarus OR Jamaica OR "South Africa" OR Belize OR Jordan OR "St. Lucia" OR "Bosnia and Herzegovina" OR Bosnia OR Kazakhstan OR "St. Vincent and the Grenadines" OR Botswana OR Lebanon OR Suriname OR Brazil OR Libya OR Thailand OR Bulgaria OR Macedonia OR Tonga OR China OR Malaysia OR Tunisia OR Colombia OR Maldives OR Turkey OR "Costa Rica" OR "Marshall Islands" OR Turkmenistan OR Cuba OR Mauritius OR Tuvalu OR Dominica OR Mexico OR Venezuela OR "Dominican Republic" OR Montenegro OR Ecuador OR Namibia)
