# Supplementary material for: Human Resources for Health-Related Challenges to Ensuring Quality Newborn Care in Low- and Middle-Income Countries: A Scoping Review
Source: Glob Health Sci Pract. 2021 Mar 31;9(1):160–76. doi: 10.9745/GHSP-D-20-00362 (PMC8087437; doi:10.9745/GHSP-D-20-00362)
Supplement: GHSP2000362_French.pdf [file GHSP2000362_French.pdf]

REVUE

Défis liés aux Ressources Humaines pour la Santé dans l'offre de Soins de Qualité aux Nouveau-nés par les Infirmières et les Sages-femmes dans les Pays à Revenu Faible et Moyen: Une revue de cadre

Nancy Bolan,[a] Karen D. Cowgill,[b] Karen Walker, [c] Lily Kak,[d] Theresa Shaver,[e] Sarah Moxon,[f] Ornella Lincetto[g]

Messages clés

- Nous avons classé en dix catégories les défis liés aux ressources humaines pour santé (RHS) dans l'offre de soins néonatals de qualité dans les structures:
  - Le manque de données et de suivi concernant les RHS
  - La mauvaise formation de base des personnels de santé (PS)
  - Le manque d'accès du personnel de santé aux lignes directrices basées sur des données probantes, à la formation continue et au développement professionnel continu
  - La faible et inéquitable répartition des personnels de santé ainsi que la lourde charge de travail
  - Le faible taux de rétention, l'absentéisme et la rotation du personnel expérimenté
  - Les mauvaises conditions de travail, y compris le bas salaire
  - Une supervision médiocre et limitée
  - Faible moral, faible motivation et mauvaise attitude, et insatisfaction vis-à-vis du travail
  - Les faiblesses de la stratégie, de la réglementation, de la gestion, du leadership, de la gouvernance et du financement
  - Les barrières structurelles et contextuelles
- La cartographie des données a fourni des informations utiles pour éclairer les stratégies récemment publiées par l'Organisation Mondiale de la Santé afin de relever systématiquement les défis et de renforcer les RHS pour les soins néonatals au niveau mondial et national.
- Le processus d'analyse thématique a également mis en évidence les interactions complexes entre les différents types de défis liés aux RHS.
- Les résultats de cette étude soutiennent de nouvelles stratégies d'action pour relever ces défis.

[a] Office of Global Health, University of Maryland School of Nursing, Baltimore, MD, USA.
[b] University of Washington Department of Global Health, Seattle, WA, USA.
[c] The George Institute for Global Health, Newtown, Australia.
[d] U.S. Agency for International Development, Washington, DC, USA.
[e] Social Solutions International, Inc., Washington, DC, USA.
[f] London School of Hygiene and Tropical Medicine, London, United Kingdom.
[g] World Health Organization, Geneva, Switzerland.
Correspondence to Nancy Bolan (nbolan@umaryland.edu).

RÉSUMÉ

Contexte: La pénurie critique de personnels de santé possédant les compétences nécessaires pour les mères et les nouveau-nés reste un défi majeur pour l'offre de soins de qualité aux mères et aux nouveau-nés, en particulier dans les pays à revenu faible et moyen. Les défis liés aux ressources humaines pour la santé (RHS) aggravent les pénuries et peuvent avoir un impact négatif sur les performances des personnels de santé et la qualité des soins. Cette étude a analysé des sources nationales afin d'identifier et de cartographier les défis liés aux RHS en ce qui concerne la qualité des soins néonatals dispensés dans les structures par les infirmières et les sages-femmes.

Méthodes: Les données probantes de cette étude ont été recueillies de manière itérative, en commençant par les documents pertinents de l'Organisation Mondiale de la Santé et en s'étendant aux articles identifiés par des recherches dans les bases de données et les manuels de référence ainsi qu'aux rapports nationaux. Les données provenant de sources nationales à partir de 2000 ont été extraites à l'aide d'un outil d'extraction de données conçu de manière itérative ; une analyse thématique a été utilisée pour cartographier les 10 catégories de défis en matière de RHS.

Résultats: Au total, 332 articles évalués par des pairs ont été analysés, dont 22 répondaient aux critères d'inclusion. Quatorze sources supplémentaires ont été ajoutées à partir de la recherche manuelle de références et de sources de littérature grise. Les données ont été classées en dix catégories de défis liés aux RHS : (1) manque de données et de suivi des personnels de santé ; (2) mauvaise de base des personnels de santé ; (3) manque d'accès des personnels de santé à des lignes directrices de pratique basées sur des données probantes, à la formation continue et au développement professionnel continu ; (4) répartition insuffisante et inéquitable des personnels de santé et lourde charge de travail ; (5) le faible taux de rétention, d'absentéisme et de rotation du personnel expérimenté ; (6) les mauvaises conditions de travail, y compris le bas salaire ; (7) la surveillance déficiente et limitée ; (8) le faible moral, la faible motivation et la mauvaise attitude, et l'insatisfaction vis-à-vis du travail ; (9) les faiblesses de la stratégie, de la réglementation, de la gestion, du leadership, de la gouvernance et du financement ; et (10) les barrières structurelles et contextuelles.

Conclusion: La cartographie fournit les informations nécessaires à l'élaboration de nouvelles stratégies de l'Organisation Mondiale de la Santé et au soutien des efforts visant à relever les défis identifiés et à renforcer les RHS pour les soins néonatals, dans le but ultime d'améliorer les soins et les résultats pour les nouveau-nés.

CONTEXTE

Les nouveau-nés sont extrêmement vulnérables ; à l'échelle mondiale, environ 2,5 millions de bébés meurent au cours de leurs 28 premiers jours de vie (la période néonatale), et environ 77 % de ces décès surviennent pendant la première semaine de vie.[1,2] En outre, près de 2 millions de mortinaissances ont lieu chaque année au cours des trois derniers mois de la grossesse ou pendant l'accouchement,[3,4] et des millions de nourrissons développent des morbidités et des problèmes neurocognitifs à court et à long terme.[5,6] La plupart des nouveau-nés peuvent survivre et s'épanouir grâce à l'accès à des soins de santé de qualité, mais la réduction de la mortalité néonatale reste lente et inégale en raison de la couverture variable des actions essentielles et de la qualité des soins dispensés par les personnels de santé (PS).[7,8]

L'accès universel à des soins de qualité pourrait prévenir 1,7 million de décès néonatals chaque année, soit 68 % des décès qui surviendront à défaut d'ici 2030.[5]

Il est essentiel de disposer d'accoucheuses et de sages-femmes qualifiées capables d'offrir des soins de qualité à l'accouchement, au nouveau-né et au niveau de la référence, car les décès néonatals précoces sont inextricablement liés à la santé maternelle et à la qualité des soins que la mère et son bébé reçoivent pendant le travail, l'accouchement et la période post-partum immédiate.[7] En outre, des personnels compétents pour les nouveau-nés - principalement des infirmières et des sages-femmes avec le soutien de médecins et d'autres spécialistes de la santé - sont nécessaires pour fournir des soins dans les structures à environ 30 millions de nouveau-nés chaque année qui ont besoin de soins en milieu hospitalier.[5,9] Alors que la majorité des femmes choisissent l'accouchement institutionnel et que la mortalité néonatale descend en dessous de 30/1000 naissances vivantes dans le monde, les prestations effectuées dans des structures de soins primaires (de base), secondaires ("soins spéciaux") et tertiaires (soins intensifs néonatals) deviennent de plus en plus importantes pour parvenir à de nouvelles baisses.[10] Les soins maternels et néonatals dans une structure sont des services cliniques fournis 24 heures sur 24 par du personnel qualifié dans des structures de soins de santé, axés sur les soins de routine et la gestion des complications.[11,12] Ensemble, les infirmières et les sages-femmes représentent le plus grand pourcentage des personnels de santé dans le monde et sont essentielles non seulement pour améliorer la santé des mères et des nouveau-nés, mais aussi pour renforcer les systèmes de santé qui garantissent que tous les nouveau-nés non seulement survivent, mais aussi s'épanouissent et réalisent leurs droits aux normes de santé et de bien-être les plus élevées possibles.[5,13-15]

Toutefois, une pénurie critique de personnels de santé possédant les compétences nécessaires en matière de soins maternels et néonatals reste un frein à l'extension de la l'offre de soins qualifiés aux mères et aux nouveau-nés, en particulier dans les pays à revenu faible et moyen (PRFM). Actuellement, les pays à revenu élevé comptent en moyenne 10,9 infirmières et sages-femmes pour 1 000 habitants, contre 2,5 et 0,9 respectivement dans les pays à revenu faible et moyen et dans les pays à faible revenu.[16] les défis liés à la disponibilité des RHS aggravent les pénuries et peuvent affecter négativement leur performance et la qualité des soins.[17,18] Comme la pandémie de coronavirus 2019 (COVID-19) surcharge les systèmes de santé dans de nombreux pays, les nouveau-nés - bien que moins susceptibles de mourir de la COVID-19 - sont exposés à un risque accru de mortalité due à d'autres affections évitables et traitables, car l'accès aux services de santé et leur disponibilité sont perturbés.[19]

Malgré l'attention accrue portée à la question des RHS depuis le Rapport mondial sur la santé et l'Assemblée mondiale de la santé de 2006, et la création de la Global Health Workforce Alliance, qui a été le fer de lance des efforts de l'Organisation Mondiale de la Santé (OMS) en matière de RHS de 2006 à 2016, l'attention spécifique portée aux RHS pour les soins néonatals est plus récente, tout comme l'accent mis sur la qualité des soins offerts.[20,21] Il est maintenant essentiel de remédier à la grave pénurie de personnels de santé compétents pour atteindre les objectifs ambitieux du plan d'action "Chaque nouveau-né" pour 2025, récemment publié, et les nouvelles normes de l'OMS visant à améliorer la qualité des soins aux nouveau-nés de faible poids et malades dans les structures de santé, dans le cadre des

progrès accomplis vers la réalisation des Objectifs de Développement Durable (ODD).[22,23] Cette revue de cadre répond à la demande d'informations opportunes pour l'Année de l'Infirmière et de la Sage-femme de l'OMS afin de garantir que ce sujet relativement négligé ait une place dans la discussion. Elle passe en revue la littérature afin d'identifier et de cartographier les données probantes, axées sur les pays, concernant les défis liés aux ressources humaines pour la santé (RHS) en matière de prestation de soins de qualité aux nouveau-nés dans des structures par les infirmières et les sages-femmes dans les pays à faible revenu et fournit la base factuelle des stratégies récemment publiées par l'OMS pour relever ces défis.[9,24] Si les soins de proximité sont également essentiels pour réduire la mortalité et la morbidité maternelles et néonatales, cet article se focalise sur les soins néonatals dispensés dans les structures de santé.

MÉTHODES

Approche

Une méthodologie de cadrage a été choisie pour cet examen parce que cette approche permet d'accumuler rapidement à grande échelle la littérature et de cartographier les preuves qu'elle contient et de déterminer l'étendue des preuves et des lacunes nécessitant des recherches supplémentaires.[25] L'approche appliquée dans cette revue de cadre utilise un processus en 5 étapes : (1) identification de la question de recherche, (2) identification des études pertinentes, (3) sélection des études, (4) consignation des données et (5) collecte, résumé et communication des résultats.[26] Les données de cette étude ont été recueillies de manière itérative, en commençant par les documents pertinents de l'OMS et les séries thématiques publiées et en s'étendant aux articles identifiés par des recherches dans les bases de données et des recherches de références manuelles, ainsi qu'aux rapports des pays et des organisations.

Recherches dans les bases de données

Cette revue s'est penchée sur la question de recherche suivante : quelles sont les données disponibles sur les défis liés aux RHS pour l'offre de soins de qualité aux nouveau-nés par les infirmières et les sages-femmes dans les PRFM. Les recherches dans la base de données visaient à trouver des articles, des commentaires et des rapports de pays à revenu faible ou moyen qui traitaient du sujet de recherche et qui ont été publiés à partir de 2000, avec le lancement des Objectifs du Millénaire pour le Développement.

Stratégie de recherche et critères de sélection

Les données pour cette revue ont été identifiées par des recherches dans PubMed, EMBASE, CENTRAL, Cumulative Index of Nursing and Allied Health Literature (CINAHL), African Journals Online (AJOL), Latin American & Caribbean Health Sciences Literature (LILACS), et des références d'articles pertinents en utilisant les termes de recherche suivants : infirmiers, infirmiers, sages-femmes, sage-femme, infirmiers-sages-femmes, nouveau-nés, nourrissons, prématurés, prématurés, faible poids à la naissance, pays en développement, pays à faible revenu, pays à revenu intermédiaire, patients hospitalisés, hôpitaux, structures de santé, centres de santé, cliniques, unités de soins néonatals, unités de soins aux nouveau-nés, unités

de soins intensifs néonatals, personnel de santé, ressources humaines pour la santé, effectifs, personnel de santé, politiques, éducation, emploi, déploiement, répartition, rétention, pénuries, salaires, motivation, performance, supervision combinés en utilisant les opérateurs booléens ET et OU et limités aux humains. La littérature grise a été recherchée par le biais d'Open Grey (www.opengrey.eu), Grey Literature Report (www.greylit.org) et Healthy Newborn Network. Les recherches ont été menées en anglais, sans restrictions linguistiques. Seuls les articles publiés à partir de 2000 ont été inclus.

La stratégie de recherche complète est fournie dans un Supplément.

Critères d'inclusion et d'exclusion

Les résultats de la recherche ont été saisis dans le logiciel Covidence (www.covidence.org), et deux évaluateurs (NEB et KDC) ont procédé à une sélection indépendante des titres et des résumés d'études en fonction de critères d'inclusion et d'exclusion. Les articles sélectionnés pour la revue du texte intégral répondaient aux critères d'inclusion conformes aux manuscrits portant sur les défis en matière de RHS pour les soins néonatals offerts par les infirmières et les sages-femmes au niveau des structures dans les PRFM.

Les critères spécifiques d'inclusion étaient les suivants:

1. Thème: relève le défi lié aux RHS
2. Prestataires: les infirmières et/ou les sages-femmes
3. Population de patients : de la naissance à 28 jours de vie (période néonatale) et soignant
4. Cadre : structures de santé au niveau primaire, secondaire ou tertiaire dans les PRFM : pays figurant sur la liste de la Banque Mondiale pour les PRFM [27]
5. Approche nationale ; données provenant du niveau national, régional, préfectoral ou de la structure sanitaire du pays
6. Années: de 2000 à nos jours

Les raisons de l'exclusion étaient les suivantes : des sources uniquement communautaires (et non des structures) ou décrivant des initiatives avec des accoucheuses traditionnelles, des agents communautaires de la santé ou des médecins uniquement ; des initiatives politiques théoriques mais non mises en œuvre ; ou des recherches menées dans des milieux de réfugiés (car ces milieux sont souvent confrontés à des défis uniques). Chaque membre de l'équipe a examiné indépendamment chaque document dans son intégralité afin de déterminer si la source devait être incluse ou exclue, et les désaccords ont été résolus par la discussion. Les résultats de la recherche et de la revue sont présentés (Figure).

Figure. Diagramme du flux des résultats de recherche et de revue des données probantes sur les ressources humaines pour la santé concernant les défis liés à la qualité des soins néonatals dans les pays à revenu faible et moyen

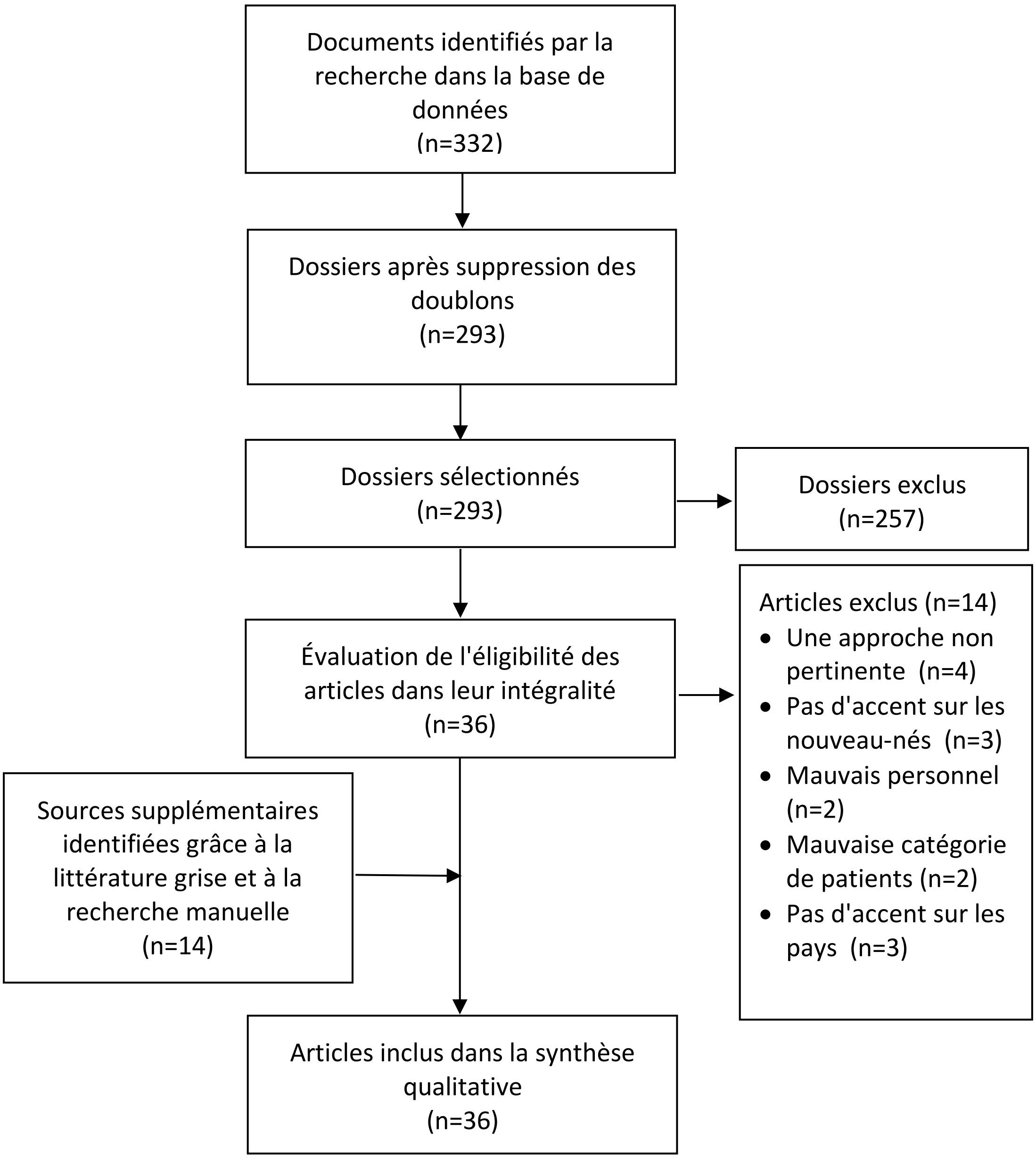

Extraction et gestion des données

Défi des RHS, exemple (détail) de défi, pays, contexte, méthodes de recherche, type de document (révisé par des pairs ou en gris), type de prestataire, année de publication, source et toute note supplémentaire. Les données extraites ont été utilisées pour créer une carte conceptuelle ou un tableau des défis en matière de RHS, puis pour regrouper des thèmes similaires en catégories par le biais d'une analyse thématique inductive décrite par Braun et Clarke.[28] Les catégories de défis ont été examinées par deux auteurs (NEB et OL), qui ont regroupé les thèmes en fonction du volume de données probantes dans chaque catégorie et ont utilisé un processus itératif et inductif pour parvenir à un consensus sur les dix dernières catégories de défis.

RÉSULTATS

Au total, 332 articles évalués par des pairs, dont 39 doublons, ont été récupérés. Les 293 résumés ont ensuite été examinés en utilisant des critères d'inclusion et d'exclusion pré-identifiés. Parmi ces sources, 22 répondaient aux critères d'inclusion (figure). Quatorze enregistrements supplémentaires ont été ajoutés à partir de la recherche manuelle de références et de sources de littérature grise, ce qui porte le nombre total d'articles inclus à 36. Les sources répondant aux critères d'inclusion ont couvert 20 des 138 PRFM. Avec 8 articles revus par des pairs, l'Inde avait la plus forte représentation, et les 19 pays restants étaient représentés par 1 ou 2 publications chacun. Une publication couvrait 4 pays (Cambodge, RDP Lao, Vietnam et Malaisie).[29]

La plupart des sources incluses étaient des publications revues par des pairs (n=33). L'année de publication s'étend de 2005 à 2019, et les sources s'appuient principalement sur des données concernant les infirmières (y compris les infirmières en obstétrique et en néonatalogie, les infirmières des unités de soins intensifs néonatals [USIN] et les étudiantes en soins infirmiers), les sages-femmes (y compris les infirmières sages-femmes auxiliaires) et, dans une moindre mesure, les médecins, les responsables médicaux, les responsables de districts sanitaires, les gestionnaires de structures et les décideurs politiques. Les méthodes de recherche utilisées dans les articles examinés étaient à la fois qualitatives (entretiens et discussions de groupe dirigées) et quantitatives (enquêtes, vérifications, tests à choix multiples et tests de compétences, questionnaires) ; les articles ont également eu recours à la revue de documents, à l'examen de carnets de patients et à l'observation pour recueillir les données. Un article s'est appuyé sur des données provenant d'un groupe national multipartite[30] et un autre sur l'analyse des données secondaires.[31] La plupart des études ont fait état de données provenant d'unités de soins intensifs néonatals, de centres de santé, d'hôpitaux ou d'hôpitaux de préfectoraux de régionaux spécifiques. Quatre articles ont utilisé des données nationales pour leur analyse (Éthiopie, Indonésie, Ghana et Ouganda).[30,32-34]

L'analyse thématique des données a permis d'établir dix catégories de défis liés aux RHS auxquels sont confrontés les infirmières et les sages-femmes pour offrir des soins de qualité aux nouveau-nés dans les structures. Un résumé des données, classées par défi en matière de RHS, est fourni dans le tableau. Les données de chaque source ont souvent été mises en correspondance avec plus d'un défi, comme indiqué dans le tableau.

Tableau. Cartographie des Ressources Humaines pour les Défis Liés à la Santé avec les Sources de la Revue

Défi des RHS	Exemples	Pays	Source Type	Source
1. Manque de données sur les RHS	La rareté des données sur les RHS comme barrière à la planification des RHS	Nigéria	RP	Adegoke et al.[35]
	Peu d'indicateurs pour la profession de sage-femme	Mongolie	RP	Kildea et al.[36]
2. Mauvaise formation de base des personnels de santé / insuffisance de programmes pour les nouveau-nés	Manque de formateurs qualifiés et de conseillers cliniques pour les sages-femmes	RD Congo	RP	Bogren et al.[37]
	La composante néonatale de base dans la formation de base des infirmières est insuffisante	Ghana	RP	Elikplim Pomevor et Adomah-Afari[38]
	Le programme d'études et les performances des infirmières de niveau licence sont médiocres par rapport à d'autres prestataires (y compris les infirmières diplômées)	Ethiopié	Rapport	Getachew et al.[32]
	Les agents de santé reçoivent une formation de base limitée en matière de soins néonatals	Kenya	RP	Aluvaala et al.[10]
	Les soins aux nouveau-nés ne constituent pas une compétence de base dans la formation générale des infirmières	Inde	RP	Campbell-Yeo et al.[39]
3. Manque d'accès des personnels de santé aux Directives et protocoles de pratique basés sur des résultats probants, CE et DPC	Non-disponibilité des lignes directrices/protocoles relatifs aux SEN et aux RN	Ethiopie	RP	Haile-Mariam et al.[41]
	Absence de lignes directrices/protocoles affichés RN	Malawi	RP	Bream et al.[42]
	Peu de protocoles disponibles pour la prise en charge des nouveau-nés malades	Ghana	RP	Elikplim Pomevor et Adomah-Afari[38]
	Guide "Aider les Bébés à Respirer" dans 25 % des structures de santé	Malawi	RP	Kozuki et al.[43]
	Non-disponibilité de protocoles pour nouveau-nés	Tanzanie	RP	Nyamtema et al.[47]
	Mauvaise diffusion des protocoles de pratique pour les infirmières des unités de soins intensifs néonatals	Thailand	RP	Jirapaet et al.[44]
	Peu de formation continue sur le nouveau-né, la RN, et les soins aux nouveau-nés de faibles poids et malades	Ghana	RP	Elikplim Pomevor et Adomah-Afari[38]
		Inde	RP	Malhotra et al.[40]
	75% des centres de santé concernés n'avaient pas de clinicien formé aux	Malawi	RP	Kozuki et al.[43]

	"soins obstétriques et néonatals d'urgence de base".			
	Des possibilités de formation continue variables pour les infirmières en néonatalogie	Inde	RP	Campbell-Yeo et al.[39]
	Accès limité au FC pour les infirmières et les sages-femmes	RD Congo	RP	Bolan et al.[66]
	Absence de stratégie de formation continue/renouvellement des compétences	Malawi	RP	Bream et al.[42]
	Peu de FC pour les personnels de santé sur la gestion des urgences liées aux RN ou aux nouveau-nés	Gambie	RP	Cole-Ceesay et al.[45]
	Pas de formation continue pour les SF	Mongolie	RP	Kildea et al.[36]
	Manque de coordination de la DPC des personnels de santé, dans le passé la DPC n'était pas obligatoire pour les SF et un contrôle de qualité de la DPC faisait défaut	Libéria	RP	Michel-Schuldt et al.[46]
4. Répartition insuffisante et inéquitable des personnels de santé, lourde charge de travail	Insuffisance PS en général	Nigéria	RP	Adegoke et al.[35]
		Malawi	RP	Bream et al.[42]
		Tanzanie	RP	Nyamtema et al.[47]
	Manque de PS dans les centres de santé/les hôpitaux préfectoraux	Népal	RP	Allen et Jeffrey[54]
	Insuffisance du personnel de l'USIN	Thailand	RP	Jirapaet et al.[44]
	Manque de personnels infirmiers spécialisés pour les soins néonatals et les nouveau-nés malades	Asie du Sud-Est (4 pays)	RP	Martinez et al.[29]
		Îles Salomon	RP	Tosif et al.[55]
		Inde	RP	Neogi et al.[31]
	Pénurie aiguë de personnels infirmiers dans les unités de soins néonatals	Kenya	RP	Nzinga et al.[48]
		Kenya	RP	Aluvaala et al.[10]
	Pénurie de PS ayant une formation en néonatologie	Rwanda	RP	Ntigurirwa et al.[49]
	Le faible nombre de personnels de santé est aggravé par une mauvaise répartition.	Indonésie	Rapport	Conseil national de recherche [33]
	Mauvaise répartition dans les zones rurales/éloignées	Népal	RP	Allen et Jeffrey[54]
		Nigéria	RP	Adegoke et al.[35]
		Inde	RP	Fischer et al.[50]
	Lourde charge de travail pour les personnels existants	Inde	RP	Amin et al.[51]
		Cambodge	RP	Ith et al.[52]

		Inde	RP	Morgan et al.[58]
	Le comblement des lacunes des personnels de niveau inférieur.	Tanzanie	RP	Prytherch et al.[53]
5. Faible rétention, mutation à l'extérieur, absentéisme	Faible rétention des personnels de santé en raison de (exemples)<br>- Décès de personnels de santé dus au VIH<br>- Migration des zones rurales vers les zones urbaines<br>- La fuite des cerveaux<br>- Appartenance au secteur privé et aux organisations non gouvernementales	Tanzanie	RP	Nyamtema et al.[47]
		Rwanda	RP	Ntigurirwa et al.[49]
		Inde	RP	Neogi et al.[11]
		Gambie	RP	Cole-Ceesay et al.[45]
	Absentéisme	Inde	RP	Neogi et al.[11]
		Tanzanie	RP	Nyamtema et al.[47]
		Tanzanie	RP	Prytherch et al.[53]
	Rotation/mutation des infirmières spécialisées en néonatalogie<br>- Vers d'autres unités<br>- Vers d'autres structures	Inde	RP	Dewez et al.[57]
		Ghana	RP	Elikplim Pomevor et Adomah-Afari[38]
		Rwanda	RP	Ntigurirwa et al.[49]
6. Mauvaises conditions de travail, notamment : bas salaires, logement insalubre, manque de matériels, de médicaments, d'équipements, d'électricité et d'eau, et mauvaises conditions de sécurité et d'infrastructure	Faible salaire et paiement irrégulier	Nigéria	RP	Adegoke et al.[35]
		Cambodge	RP	Ith et al.[52]
		Tanzanie	RP	Prytherch et al.[53]
	Mauvaises conditions d'hébergement	Gambie	RP	Cole-Ceesay et al.[45]
	Manque de sécurité dans le travail<br>- Manque d'équipements de protection<br>- Peur de vol, agressions, violence basée sur le genre, agression par les familles	Nigéria	RP	Adegoke et al.[35]
		Gambie	RP	Cole-Ceesay et al.[45]
		Inde	RP	Morgan et al.[58]
		Tanzanie	RP	Prytherch et al.[53]
	Manque de matériel, de médicaments essentiels, d'équipements	Nigéria	RP	Adegoke et al.[35]
		Asie du Sud-Est (4 pays)	RP	Martinez et al.[29]
		Ethiopie	RP	Haile-Mariam et al.[41]
		Inde	RP	Morgan et al.[58]

	Manque de moyens techniques, retards dans les travaux de maintenance	Îles Salomon	RP	Tosif et al.[55]
	La mise en place d'un service constituant une barrière aux soins	Inde	RP	Neogi et al.[56]
		Malawi	RP	Bream et al.[42]
7. Une supervision limitée et médiocre	Capacité limitée pour la surveillance de routine	RDP Lao	RP	Horiuchi et al.[59]
	Pas de mentors sur place ni de soutien technique pour les SF ayant des compétences limitées	Nigéria	RP	Adegoke et al.[35]
	Manque de supervision clinique dans les unités de soins intensifs néonatals	Thailand	RP	Jirapaet et al.[12]
	Supervision punitive, pas de retour d'information, manque de confidentialité	Tanzanie	RP	Prytherch et al.[53]
	Les problèmes restent non résolus après les visites de supervision	RDP Lao	RP	Horiuchi et al.[59]
8. Faible moral, manque de motivation et mauvaise attitude, insatisfaction	Poor conditions, salaries, lack of incentives	Gambie	RP	Cole-Ceesay et al.[45]
	Absence de plan de carrière et de possibilités de promotion pour les personnels de santé	Nigéria	RP	Adegoke et al.[35]
		Mongolie	RP	Kildea et al.[36]
		Tanzanie	RP	Prytherch et al.[53]
	Absence de plan de carrière et de possibilités de promotion pour les personnels de santé	Inde	RP	Amin et al.[51]
		Inde	RP	Dewez et al.[57]
	Impuissance sur les conditions de travail, manque de contrôle sur les mutations	Tanzanie	RP	Prytherch et al. 2012[53]
		Inde	RP	Dewez et al.[57]
	Le moral est bas en raison de la mortalité élevée des patients et de l'incapacité à offrir de bons soins due aux mauvaises conditions	Gambie	RP	Cole-Ceesay et al.[45]
		Inde	RP	Dewez et al.[57]
		Tanzanie	RP	Prytherch et al.[53]
9. Faiblesses de la stratégie, de la réglementation, de la gestion, du leadership, de la gouvernance et du financement	Absence de cadres stratégiques et réglementaires pour les soins néonatals et les protocoles cliniques	Ghana	RP	Elikplim Pomevor et Adomah-Afari[38]
		Cambodge	RP	Ith et al.[52]
	Cadre de pratique limité pour les sages-femmes, non conforme au cadre de pratique international	Mongolie	RP	Kildea et al.[36]
	Pas de normes de compétence reconnues au niveau national pour les SF et les infirmières	Mongolie	RP	Kildea et al.[36]
		Indonésie	Rapport	Conseil national de recherche [33]

	Absence de parité entre les salaires des travailleurs du secteur publique et ceux des travailleurs du secteur privé.	Tanzanie	RP	Prytherch et al.[53]
	Absence de normes en matière de personnel par niveau de structure	Ghana	Rapport	Ministère de la Santé Ghana[30]
	Non-prise en compte par la direction du besoin de ratios de type USI dans les unités de soins néonatals	Inde	RP	Dewez et al.[57]
	Manque de soutien de la part de l'équipe de gestion de l'hôpital, de la direction de la structure ou des autorités	Inde	RP	Dewez et al.[57]
		Inde	RP	Fischer et al.[50]
	Absence de descriptions de poste, de directives néonatales ou d'orientation dans les unités de soins aux nouveau-nés	Kenya	RP	Nzinga et al.[48]
		Tanzanie	RP	Prytherch et al.[53]
		Inde	RP	Campbell-Yeo et al.[39]
	Faible productivité des personnels de santé	Tanzanie	RP	Nyamtema et al.[47]
	Une gestion peu encourageante, des horaires de travail non flexibles	Tanzanie	RP	Prytherch et al.[53]
	Les déficiences des ressources humaines au niveau de la direction se traduisent par une gestion inefficace, notamment en ce qui concerne le budget.	RDP Lao	RP	Sychareun et al.[60]
	La non-priorisation de la santé des nouveau-nés par les décideurs politiques dans le passé	Ghana	Rapport	Ministère de la Santé Ghana[30]
		Tanzanie	RP	Prytherch et al.[53]
	Faiblesse des associations professionnelles qui n'améliorent pas les conditions des personnels de santé	Ouganda	RP	Mbonye et al.[34]
		Tanzanie	RP	Nyamtema et al.[47]
		Gambie	RP	Cole-Ceesay et al.[45]
10. Barrières structurelles et contextuelles	Manque de prise en compte des besoins des nouveau-nés en matière de soins spécialisés	Inde	RP	Campbell-Yeo et al.[39]
	Manque de perception par la communauté du fardeau des maladies néonatales	Népal	RP	Allen et Jeffrey[54]
	Négligence des nouveau-nés de sexe féminin	Inde	RP	Morgan et al.[58]
	Les femmes en âge de procréer n'ont pas le pouvoir de déterminer	Népal	RP	Brunson[61]

	le comportement à adopter en cas d'urgence			

Abréviations: FC, formation continue; FPC, formation professionnelle continue; SEN, soins essentiels aux nouveau-nés; RHS, ressources humaines pour la santé; PS, personnel de santé; USI, unité de soins intensifs; SF, sage-femme; NN, nouveau-né; USIN, unité de soins intensifs néonatals; RN, réanimation néonatale; RP, Revue par les pairs.

Catégories de défis en matière de RHS

1. Manque de données et de suivi sur les RHS nécessaires à la santé maternelle et néonatale

Les sources étudiées ont fait état d'un manque de données sur les RHS concernant la disponibilité, la répartition, les tendances et les besoins en personnel.[35,36] Par exemple, le Nigeria connaît une grave pénurie de RHS, en particulier pour les structures de santé dans les zones rurales, et la tâche problématique de planification de l'embauche et de la répartition des personnels est rendue plus difficile par le manque de données essentielles sur les RHS.[35] Similarly, in Mongolia, Kildea et al.[36] note qu'en plus d'une pénurie générale d'infirmières, de sages-femmes et de professionnels paramédicaux pour les soins aux mères et aux nouveau-nés, il est difficile de déterminer les effectifs nécessaires des différents types de personnels de santé, en raison de l'absence de paramètres permettant de mesurer ces effectifs.

2. Mauvaise formation de base des personnels de santé et insuffisances du volet "nouveau-né"

Selon certaines sources, la formation de base en matière de santé maternelle est souvent faible, notamment en ce qui concerne les connaissances et les compétences en matière de soins néonatals offertes à tous les niveaux, et il existe peu de programmes de formation d'infirmières spécialisées en néonatalogie dans les pays à revenu faible et moyen.[10,32,37-39] Au Kenya, par exemple, le personnel de santé reçoit une formation de base limitée en matière de soins néonatals, et acquiert la plupart de ses expériences pratiques lors de stages cliniques ou d'internats dans les hôpitaux.[10] De même, il a été observé que les soins néonatals ne faisaient pas partie des compétences de base de l'enseignement général des soins infirmiers en Inde [39]; au Ghana, on a constaté que le volet "nouveau-né" était insuffisant dans la formation de base des infirmières.[38]

3. Manque d'accès des personnels de santé aux directives et protocoles de pratique basés sur des données probantes, à la formation continue et au développement professionnel continu

L'indisponibilité ou le manque d'accès aux lignes directrices ou aux protocoles actuels fondés sur des données probantes, à la formation continue et au développement personnel continu pour les soins néonatals est une plainte courante dans les sources des PRFM.[36,38-47] Jirapaet et al.[44] a fait état d'une étude menée dans quatre unités de soins intensifs néonatals en Thaïlande, où les chercheurs ont constaté un manque de diffusion des protocoles de pratique aux infirmières. De même, une étude menée en Éthiopie dans tous les hôpitaux et centres de santé où se déroulent les accouchements a fait état d'un manque de disponibilité des directives et protocoles sur les soins essentiels aux nouveau-nés et la réanimation néonatale.[41] La formation continue, définie comme un processus d'apprentissage permanent, est la pierre

angulaire du maintien des compétences et est étroitement liée à la qualité des soins et à la sécurité des patients.[21] le développement professionnel continu se réfère au processus de suivi et de documentation des compétences, des connaissances et de l'expérience acquises par le personnel de santé, tant formellement qu'informellement, au-delà de toute formation de base. Michel-Schuldt et al.[46] a noté que la formation professionnelle continue n'est pas obligatoire pour les sages-femmes au Liberia et, plus généralement, qu'il y a un manque de coordination de la formation professionnelle continue pour les personnels de santé ainsi qu'une absence de contrôle de la qualité de la formation des sages-femmes. En Gambie, Cole-Ceesay et al.[45] a constaté que la formation continue des personnels de santé sur la gestion de la réanimation néonatale ou des urgences pour les nouveau-nés était peu développée ; le Ghana a fait état d'une formation continue limitée sur les soins néonatals, la réanimation néonatale ou les soins aux nouveau-nés à faible poids et malades.[38]

4. Répartition insuffisante et inéquitable des ressources humaines et de la charge de travail

Les sources ont donné de nombreux exemples de répartition insuffisante et équitable des personnels de santé - en particulier le manque d'accoucheuses et d'infirmières néonatales qualifiées - avec pour conséquence une lourde charge de travail pour le personnel existant et des ratios d'effectifs inacceptables.[10,29,33,35,42,44,47-56] Une étude menée dans deux unités de soins néonatals des hôpitaux publiques de Nairobi a fait état d'un ratio d'effectifs de 1 infirmière pour 15 bébés.[48] Aluvaala et al.[10] a constaté que sur les 22 unités néonatales hospitalières étudiées au Kenya, 6 présentaient une telle pénurie de personnel qu'elles n'étaient pas en mesure d'affecter ne serait-ce qu'une infirmière à chaque unité néonatale. De même, une étude portant sur 9 unités de soins intensifs néonatals en Inde a fait état de ratios de personnel inéquitables allant de 1 pour 4 à 1 pour 8 dans les structures privées, tandis qu'ils allaient de 1 pour 25 à 1 pour 35 dans les structures publiques.[51] Une étude menée dans plusieurs pays d'Asie du Sud-Est a révélé que les unités néonatales de Hanoï prenaient systématiquement en charge 50 % de patients de plus que les lits et le personnel alloués et qu'elles étaient donc obligées de mettre deux patients dans un lit.[29] Neogi et al.[56] a évalué 8 unités spéciales de soins néonatals dans des hôpitaux de district ruraux de toute l'Inde et a constaté que le ratio infirmière/nouveau-né semblait jouer un rôle essentiel dans l'amélioration de la survie des nouveau-nés dans ces unités : près de 15 % de la variation du taux de mortalité néonatale entre les unités pourrait s'expliquer par le ratio infirmière/nouveau-né. En plus de la lourde charge de travail du personnel en place dans les structures sanitaires rurales, la Tanzanie a fait état d'un déséquilibre entre la proportion de personnel de santé qualifié et les personnels de niveau inférieur dans les structures rurales de soins maternels et néonatals, 43 % de l'effectif étant constitués de personnels de niveau inférieur (par exemple, les aides-soignants, les assistants cliniques et les accoucheuses).[53]

5. Faible rétention, absentéisme et rotation du personnel expérimenté

De nombreuses sources décrivent le faible taux de rétention du personnel de santé maternelle et néonatale.[11,38,45,47,49,53,57] L'absentéisme a été signalé[11] en raison du fait que des personnels

de santé du secteur publique travaillent également dans le secteur privé en Inde. Une étude réalisée dans deux centres de santé ruraux en Tanzanie a révélé que les personnels de santé ont souvent des activités parallèles génératrices de revenus, comme l'agriculture, le commerce ou d'autres activités liées à la santé, en complément de leur salaire, ce qui peut entraîner des distractions au travail et de l'absentéisme.[53] De nombreux articles inclus dans cette revue ont également cité la rotation au sein de la structure ou le transfert vers une autre structure comme une préoccupation majeure. Dans une étude portant sur six structures de santé au Ghana, de nombreux personnels de la santé interrogés étaient préoccupés par la rotation annuelle du personnel néonatal, car elle entravait la qualité des soins en raison de la perte de personnel expérimenté et du temps nécessaire à la mise à niveau du nouveau personnel.[38] Dewez et al.[57] ont décrit que lorsque les infirmières ont été formées en Inde aux soins néonatals et ont été mutées dans d'autres services, elles ont perdu leur motivation et leurs compétences en matière de soins néonatals.

6. Mauvaises conditions de travail

On signale souvent des salaires médiocres, des paiements incohérents et des logements de mauvaise qualité.[29,31,35,41,42,45,52,53,55,58] Une étude qualitative réalisée avec des personnels de santé au Cambodge [52] a rapporté que les salaires des accoucheuses qualifiées étaient faibles, de sorte qu'elles ne dispensent des soins postnatals à la mère et au nouveau-né que si des membres de la famille effectuent des paiements supplémentaires. Comme indiqué dans le défi 5 des RHS, les personnels soignants crée souvent des activités génératrices de revenus supplémentaires pour compléter leurs rémunérations.[53] Le manque d'équipement, de matériels, de médicaments, d'électricité et d'eau est souvent signalé dans la littérature.[29,35,41,58] Un manque de matériels et d'équipements de protection, tels que des gants et des masques, peut affecter la sécurité en matière de déchets dangereux. Il peut être exacerbé lors d'épidémies telles que la pandémie actuelle de COVID-19 et contribue à la mauvaise qualité des soins.[19] En outre, le manque de sécurité des personnels de santé dû aux reproches et aux agressions apparaît dans la littérature ; un article en provenance d'Inde a noté que les prestataires renvoient les cas compliqués afin de ne pas être agressés par les membres de la famille en cas de mauvais résultats des soins maternels et néonatals.[58]

7. Une supervision limitée et médiocre

La mauvaise supervision est citée comme un problème conduisant à une mauvaise rétention des ressources humaines, à un moral et une motivation bas et à une mauvaise qualité des soins.[35,44,53,59] Horiuchi et al.[59] a noté que bien que les visites de supervision externes soient une approche courante pour promouvoir le changement de comportement des personnels de santé néonatals, renforcer les compétences et maintenir la qualité des soins, ces visites ne sont souvent pas réalisables dans des milieux aux ressources limitées, comme la RDP Lao, en raison des coûts élevés et des besoins en ressources humaines. Les auteurs précisent que la capacité à mettre en œuvre une supervision de routine est minimale ; cependant, lorsque des visites de supervision ont lieu, les problèmes identifiés restent souvent non résolus.[59] De même, les agents de santé maternelle et néonatale interrogés en Tanzanie ont déclaré que la

supervision et l'évaluation des performances étaient punitives et manquaient de confidentialité.[53]

8. Faible moral, faible motivation et mauvaise attitude, et insatisfaction professionnelle

Le moral et la motivation ont été fréquemment mentionnés dans les sources.[35,36,45,51,53,57] La faible motivation et l'insatisfaction professionnelle sont liées à divers facteurs, notamment les bas salaires, les mauvaises conditions de travail, le manque de possibilités de carrière et de promotion, l'absence de contrôle sur les mutations, l'insuffisance de la formation et de l'orientation technique, l'épuisement professionnel et le stress lié à une lourde charge de travail, et une supervision démoralisante.[36,45,51,53] Plusieurs articles ont fait état d'un stress élevé et d'un moral bas en raison d'une mortalité maternelle et néonatale élevée.[45,51] et la culpabilité des personnels de santé et les sentiments d'impuissance liés à l'incapacité d'offrir de meilleurs soins aux patients.[57] Le stress et la fatigue ont été signalés chez les personnels des unités de soins intensifs néonatals en Inde et en Thaïlande.[44,51] On a également constaté que le faible moral et les mauvaises attitudes affectent la prestation des soins ; par exemple, la passivité des prestataires de soins face à des situations d'urgence mettant la vie en danger en Tanzanie a contribué aux mauvais résultats.[47]

9. Faiblesses de stratégies, de réglementations, de gestion, de leadership, de gouvernance et de financement

Les sources documentant les faiblesses dans le domaine de la stratégie et de la gestion étaient de nature variée.[30,33,34,36,38,39,47,48,50,52,53,57,60] Une étude menée en Tanzanie a conclu que les facteurs qui découragent les prestataires de soins maternels et néonatals pourraient être divisés en deux catégories : ceux qui ont trait aux conditions d'emploi (par exemple, liés aux stratégies) et ceux qui ont trait à l'organisation des processus de travail (par exemple, liés à la gestion des structures).[53] Le leadership, la gouvernance et les financements évoqués ont mis en évidence le manque de priorité accordée aux soins néonatals et le faible financement de ces soins dans les budgets nationaux.[34]

10. Barrières structurelles et contextuelles

Diverses barrières structurelles et contextuelles potentielles à l'offre de soins de haute qualité aux nouveau-nés viennent s'ajouter aux défis en matière de RHS mentionnés ci-dessus.[39,54,58,61] Une étude menée dans les zones rurales du Népal a révélé qu'une barrière importante à l'amélioration des soins néonatals était le manque de perception de la charge de morbidité néonatale dans la communauté d'où proviennent les prestataires de soins de santé et que sans la perception d'un problème, les prestataires sont peu incités à améliorer leurs performances professionnelles.[54] Un rapport du Bihar, en Inde, a observé une préférence pour les enfants de sexe masculin et la négligence des nouveau-nés de sexe féminin, au point que les familles menacent parfois les personnels de santé qui tentent de réanimer les nouveau-nés de sexe féminin.[58] Les préjugés basés sur le sexe affectent les soins néonatals de diverses autres manières, qu'il s'agisse de la dynamique du pouvoir pour la majorité des sages-femmes et des infirmières ou de la dynamique du pouvoir basée sur le sexe liée aux décisions relatives

à l'accouchement, comme le comportement de recherche de soins d'urgence, tel que signalé au Népal.[61]

DISCUSSION

À notre connaissance, cette étude est la première à aborder de manière exhaustive les défis en matière de RHS pour l'offre de soins de qualité aux nouveau-nés dans les PRFM. En menant cette étude, nous avons cherché à mieux comprendre les défis liés aux RHS pour l'offre de soins néonatals de qualité en structure par les infirmières et les sages-femmes dans les PRFM en identifiant des thématiques concrètes à aborder aux niveaux national, régional et international. Nous avons cherché à synthétiser les données probantes identifiées dans les sources nationales et à fournir des informations nécessaires pour éclairer les stratégies de renforcement des RHS pour les soins néonatals au niveau mondial et national, en renforçant la capacité des pays à répondre aux nouvelles normes de l'OMS pour les soins aux nouveau-nés de faible poids et malades, aux objectifs de l'ENAP 2025 et aux ODD. Les objectifs de cette série de défis urgents sont d'améliorer la qualité des soins et de réduire la mortalité, les mortinaissances et la morbidité à court et long terme des nouveau-nés.

La faiblesse des données et du suivi des RHS rend la prise de décision et la planification des effectifs particulièrement difficiles.[5,18] Des données essentielles sur la disponibilité des personnels de santé, en particulier des infirmières et des sages-femmes, font souvent défaut.[36] Il manque des données montrant que la structure nécessaire à la combinaison des compétences du personnel fait défaut, ce qui augmente les difficultés de planification.[7] Pour améliorer la qualité des soins offerts aux nouveau-nés et pour faciliter la défense de leurs intérêts, il faut disposer de données sur les personnes, les lieux et les modalités de prise en charge des nouveau-nés par les personnels de santé.[62] La recherche sur les indicateurs et les cadres de suivi des RHS pour guider la planification nationale est une priorité mondiale.[5]

Les données recueillies dans plusieurs pays montrent que la formation de base des médecins, des infirmières et des sages-femmes est souvent faible ; en outre, la multiplication rapide des écoles privées non accréditées soulève des questions quant à leur qualité.[63] Le volet "soins néonatals" est souvent limité dans les programmes de formation de base des personnels concernés.[64] L'analyse des goulots d'étranglement pour les soins hospitaliers des nouveau-nés de faible poids et malades a révélé un manque de formation axée sur les compétences dans les pays où la mortalité néonatale est la plus élevée, et a indiqué que la formation dans ce domaine est incohérente et mal structurée.[12,17] Une partie de la difficulté provient du manque de professeurs qualifiés et de cliniciens compétents pour enseigner les soins néonatals, ce qui limite les possibilités d'acquérir des compétences en matière de soins néonatals pendant la période de formation de base.[37,64] Les moyens nécessaires pour améliorer la formation des enseignants en matière de contenu néonatal et d'assurer la rétention des enseignants ne sont pas abordés dans la littérature et constituent un domaine où la recherche est nécessaire.

Il est souvent difficile, voire impossible, pour les infirmières et les sages-femmes d'avoir accès à des lignes directrices et à des protocoles pertinents et actualisés, ainsi qu'à des possibilités de formation continue et de perfectionnement professionnel, en particulier dans

les zones rurales.[21,65,66] Pour les soins spécifiques aux bébés de faible poids et malades, des recherches menées dans 12 pays présentant des taux de mortalité néonatale élevés ont révélé que le niveau de formation ou de perfectionnement basé sur les compétences, y compris la formation continue et le recyclage, était insuffisant ou inexistant, en particulier dans les structures de bas niveau.[12] Les directives changent régulièrement et, par conséquent, la diffusion et les méthodes d'actualisation des connaissances sont essentielles ; cependant, la simple diffusion de directives écrites est inefficace.[67] La plupart des urgences obstétriques et néonatales ont lieu dans des structures de santé périphériques, dont l'accès est difficile avec les programmes de formation classiques et qui nécessitent des stratégies d'apprentissage innovantes.[68] Les possibilités de relever ces défis sont désormais disponibles grâce à des ensembles d'apprentissage basés sur les expériences et les compétences qui intègrent la formation par simulation et le mentorat, des initiatives d'apprentissage numérique en ligne et d'autres outils d'apprentissage innovants qui utilisent la technologie tels que les outils de formation basés sur la réalité virtuelle, la décision clinique et les outils d'apprentissage et de soutien dans les services.[66,69,70] Cependant, des recherches supplémentaires sont nécessaires sur le e-learning et le m-learning, en particulier dans les PRFM, qui mesurent les résultats des nouveau-nés comme un résultat principal.[70]

La pénurie de ressources humaines (thème 4) est l'un des principaux facteurs expliquant la persistance de taux de mortalité élevés chez les femmes et les nouveau-nés dans de nombreux pays.[5,71] Moins d'un pays sur six ayant la charge la plus élevée de mortalité maternelle et néonatale atteint le seuil minimum nécessaire pour offrir un ensemble de soins de base, soit 23 médecins, sages-femmes et infirmières pour 10 000 habitants.[63] Un nombre insuffisant de personnels de santé, combiné à de mauvaises conditions de travail et au peu d'incitations pour le personnel à vivre et à travailler dans des zones reculées ou parmi les populations défavorisées, entraîne une répartition inégale. Des déséquilibres existent non seulement dans le nombre et la répartition géographique des personnels de santé disponibles, mais aussi dans le secteur de l'emploi (publique/privé)[72] et dans l'éventail des compétences en matière de ressources humaines. La plupart des pays ont encore trop peu de spécialistes par rapport aux besoins de santé de leur population ; les pénuries sont particulièrement évidentes pour les médecins spécialistes (par exemple, néonatologistes, chirurgiens, obstétriciens et anesthésistes) et les infirmières néonatales, avec peu de programmes disponibles pour former ces personnels dans les milieux à faibles revenus.[64,71] En revanche, dans les pays à revenu élevé, les infirmières néonatales constituent l'épine dorsale de la structure de soins aux nouveau-nés et à leur famille, notamment grâce à des rôles élargis tels que celui d'infirmière clinicienne spécialisée en néonatologie.[12,73]

La pénurie de personnel de santé et l'inégalité de la répartition augmentent la charge de travail du personnel en place, ce qui entraîne un ratio personnel/patient élevé et un stress accru, entrave la capacité à fournir des soins de haute qualité et influence directement le pronostic des patients.[31,40] Des recherches sur les effectifs et la charge de travail des infirmières des unités de soins intensifs néonatals dans les pays à revenu élevé ont montré que le manque de personnel par rapport aux directives nationales était associé à un risque accru d'infection nosocomiale chez les nouveau-nés de très faible poids de naissance.[74,75] En

revanche, la dotation en personnel infirmier de ces unités réduit la mortalité hospitalière dans une proportion de 1 pour 1.[76,77] Il n'existe pas de recommandations globalement acceptées pour les ratios de personnel aux différents niveaux de la prestation de soins néonatals, mais nous pouvons comparer les ratios dans les PRFM avec les ratios recommandés au Royaume-Uni, à savoir 1 infirmière spécialisée en néonatalogie pour 1 patient de soins intensifs néonatals et 1 infirmière ou sage-femme diplômée pour 4 patients de soins spéciaux.[78]

Le faible taux de rétention, l'absentéisme et la rotation des infirmières expérimentées hors des unités néonatales peuvent à la fois créer les dynamiques ci-dessus et contribuer à leur aggravation (thème 5). La majorité des pays (81 %) affichent un personnel qui préfère fortement les zones urbaines, ce qui peut être lié à de nombreux facteurs, tels que les possibilités accrues de pratique privée et le manque d'attrait des zones rurales et éloignées en raison des mauvaises conditions de travail, des logements inadaptés, des possibilités limitées de développement professionnel et des possibilités d'éducation limitées pour les enfants.[72,79]

En outre, même les personnels de santé les mieux formés et les plus motivés ont besoin d'un environnement favorable et propice pour travailler efficacement, notamment d'infrastructures bien entretenues et d'un approvisionnement fiable en médicaments, en matériel et en technologies (thème 6). Les salaires et les conditions de travail médiocres contribuent à d'autres problèmes, tels qu'un faible taux de rétention, en particulier dans les régions reculées, ainsi qu'à un moral et une attitude médiocres.[17] Une cartographie systématique des barrières à l'offre de soins de sage-femme de qualité a révélé l'absence systématique de conditions de travail sûres, telles que la disponibilité de moyens d'élimination des objets tranchants, d'eau pour le lavage des mains et d'équipements de protection de base tels que des gants.[80] De même, une enquête menée dans 364 structures de santé en Afrique, en Asie et en Amérique latine a révélé que les matériels et équipements essentiels étaient largement indisponibles et a conclu que le personnel était souvent incapable de pratiquer les procédures fondamentales et que les femmes, les enfants mort-nés et les nouveau-nés mouraient inutilement en conséquence.[81]

La supervision formative (thème 7) est un autre outil qui est utilisé dans la plupart des contextes pour soutenir les personnels de santé et améliorer leurs performances professionnelles. Si elle est correctement effectuée, la supervision peut être un mécanisme permettant d'assurer le développement professionnel, d'améliorer la satisfaction au travail et d'accroître la motivation.[67] Cependant, la réalité est que les visites de contrôle n'atteignent souvent pas leur objectif. Les superviseurs peuvent manquer de compétences, d'outils et de moyens de transport pour assurer une supervision de qualité.[17,67] Les études prévues pour évaluer la surveillance continue autogérée par des revues en pairs et des sessions de retour d'information comme moyen plus durable d'améliorer la qualité des soins pourraient s'avérer une alternative aux approches actuelles de supervision.[59]

La qualité de la prestation de services de soins dépend de la motivation des professionnels de la santé - un facteur essentiel pour qu'ils soient prêts à maintenir leurs compétences professionnelles, à s'appliquer à leur travail et à rester à leur poste.[67,82-85] Le faible moral des personnels soignants et les niveaux élevés de stress et d'épuisement ont été identifiés dans la

littérature comme un problème souvent négligé (thème 8) et ont été largement documentés parmi les infirmières, infirmiers et sages-femmes des unités de soins intensifs néonatals.[51,80,86] Les chercheurs suggèrent que la motivation peut à la fois influencer directement les performances et atténuer l'effet d'autres facteurs ; ainsi, la motivation - et les interventions qui améliorent la satisfaction au travail (par exemple, les salaires, les conditions de travail) - sont susceptibles d'être des déterminants importants des performances et du maintien au poste.[67]

Les défis liés à la faiblesse ou à l'absence de stratégies, de réglementations, de gestion, de leadership, de gouvernance et de financement (thème 9) sont essentiels à l'offre de soins néonatals de qualité.[30,33,34,36,38,39,47,48,50,52,53,57,60] Les exemples comprennent le manque d'harmonie entre la politique nationale définissant les domaines de compétences légaux pour les différents personnels et la réglementation des personnels, les descriptions de poste, la formation de base ou la pratique réelle.[72] Il y a également un manque de réglementation des établissements d'enseignement et des prestataires de soins de santé du secteur privé,[87] et l'absence des stratégies nécessaires, telles que des ratios personnel/patient bien définis, des systèmes de référence, des critères de sortie et des niveaux de soins harmonisés.[12] Tous les programmes de santé - qu'ils soient financés par les gouvernements, les partenaires au développement, la société civile ou le secteur privé - doivent contribuer aux priorités nationales définies par le gouvernement ; la réalisation de cet objectif nécessite une meilleure gouvernance et une meilleure coordination entre les systèmes nationaux et infranationaux.[87] Des défenseurs au niveau national sont nécessaires pour faire progresser les soins de haute qualité pour les nouveau-nés, notamment les décideurs politiques, les personnes clés au sein des organismes professionnels, les académiciens et les institutions nationales. En termes de financement de la santé, le manque de financement durable et coordonné des soins néonatals reste un problème,[34] et la mobilisation d'un financement suffisant avec un meilleur flux de trésorerie est nécessaire.[87]

Enfin, la littérature décrit les barrières structurelles et contextuelles qui exacerbent d'autres défis liés aux RHS, en particulier le faible statut social des professions de soins et l'inégalité des sexes dans une main-d'œuvre à prédominance féminine. Par exemple, une cartographie des barrières à l'offre de soins de sage-femme de qualité a identifié l'inégalité entre les sexes et le manque d'autonomisation des femmes comme étant les barrières les plus importantes conduisant au stress et à l'épuisement professionnel, qui à leur tour conduisent à la désautonomisation, à la diminution de l'estime de soi et finalement à l'adoption de comportements négatifs.[80,88] Le manque de confiance en soi a également été largement signalé dans le secteur des soins infirmiers, une profession également dominée par les femmes, ainsi que le stress élevé et l'épuisement professionnel, comme indiqué ci-dessus.[21,89]

Campbell[90] a écrit que la seule voie pour atteindre la qualité des soins est "par l'intermédiaire du personnel de santé", et qu'une couverture universelle efficace - les RHS assurant à la fois la disponibilité et la qualité de la couverture des services de santé nécessaires - est le grand défi pour tous les pays. Cette conclusion a été reprise par la commission du Lancet Global Health sur les systèmes de santé de haute qualité, qui propose un "redémarrage" des systèmes de santé étant donné l'ampleur des déficits de qualité.[20] Des ressources humaines compétentes

et les ressources physiques nécessaires sont indispensables à tout moment pour éviter la mortalité évitable des femmes, des mort-nés et des nouveau-nés.[81] Ces principes, qui renforcent l'importance du personnel de santé mondial, ont été énoncés dans le Rapport sur la santé dans le monde 2006 et avec la création de l'Alliance Mondiale pour les Personnels de Santé. L'OMS a renforcé les stratégies mondiales visant à relever les défis en matière de RHS dans la Stratégie mondiale sur les RHS: Workforce 2030.[69] Compte tenu des nouveaux objectifs et normes en matière de santé néonatale pour 2025 concernant les soins aux petits enfants et aux malades, l'OMS a récemment publié des stratégies de RHS spécifiques aux nouveau-nés, éclairées par les défis identifiés dans cet article, afin d'aborder l'accessibilité et la qualité des soins de Cette couche de la population extrêmement vulnérable.[9] Les défis liés à l'offre de soins de qualité aux mères et aux nouveau-nés dans les pays sont complexes, interdépendants et entrelacés avec des défis sociaux et structurels plus larges et nécessiteront une attention constante et un classement par ordre de priorité à tous les niveaux du système de santé si nous voulons réussir à atteindre les ODD d'ici 2030.

Limites

Restreindre et cibler la question de recherche était un défi et la définition large des soins aux nouveau-nés a permis d'obtenir une grande quantité d'informations. Bien qu'un processus d'analyse thématique itératif et inductif ait été utilisé pour identifier les défis liés aux RHS en matière d'offre de soins de qualité aux nouveau-nés, certains chevauchements ont été constatés dans les domaines conceptuels, ce qui a pu masquer l'importance, la nuance ou l'interconnexion des catégories de défis. En outre, notre analyse n'est pas représentative de tous les PRFM, étant donné que de nombreux pays ne disposent pas de données sur leurs défis respectifs en matière de RHS, qui peuvent être uniques et spécifiques au contexte.

CONCLUSIONS

À dix ans seulement de l'échéance des ODD, il est essentiel de garantir l'accès à des soins de qualité à tous les nouveau-nés qui ont besoin de soins en structure. Cependant, le manque d'un nombre suffisant d'assistants sociaux ayant des compétences en matière de soins néonatals constitue une lacune critique. Cette revue a examiné des articles centrés sur les pays afin d'explorer les défis liés aux RHS pour la prestation de soins néonatals de qualité en structure par les infirmières et les sages-femmes dans les pays à faible revenu et à revenu moyen. L'étude a identifié et mis en évidence dix défis liés aux RHS et a interprété les données. La cartographie fournit les informations nécessaires pour éclairer les nouvelles stratégies de l'OMS et soutenir les efforts visant à relever les défis identifiés et à renforcer les RHS pour les soins néonatals, dans le but ultime d'améliorer les soins aux nouveau-nés ainsi que leurs issues.

REFERENCES

1. United Nations Inter-agency Group for Child Mortality Estimation (UN IGME). *Levels &*

Trends in Child Mortality: Report 2019, Estimates Developed by the United Nations Inter-Agency Group for Child Mortality Estimation. UN IGME; 2019. https://www.unicef.org/media/60561/file/UN-IGME-child-mortality-report-2019.pdf

2. Sankar MJ, Natarajan CK, Das RR, Agarwal R, Chandrasekaran A, Paul VK. When do newborns die? A systematic review of timing of overall and cause-specific neonatal deaths in developing countries. *J Perinatol.* 2016;36(S1)(Suppl 1):S1-S11. CrossRef. Medline
3. Lawn JE, Blencowe H, Oza S, et al.; Lancet Every Newborn Study Group. Every Newborn: progress, priorities, and potential beyond survival. *Lancet.* 2014;384(9938):189-205. CrossRef. Medline
4. United Nations Inter-agency Group for Child Mortality Estimation (UN IGME). *A Neglected Tragedy: The Global Burden of Stillbirths*. UN IGME; 2020. Accessed December 22, 2020. https://www.unicef.org/reports/neglected-tragedy-global-burden-of-stillbirths-2020
5. World Health Organization (WHO). *Survive and Thrive: Transforming Care for Every Small and Sick Newborn.* WHO; 2019. Accessed December 22, 2020. https://www.unicef.org/media/58076/file
6. Lawn JE, Blencowe H, Darmstadt GL, Bhutta ZA. Beyond newborn survival: the world you are born into determines your risk of disability-free survival. *Pediatr Res.* 2013;74(S1)(Suppl 1):1-3. CrossRef. Medline
7. Bhutta ZA, Salam RA, Lassi ZS, Austin A, Langer A. Approaches to improve Quality of Care (QoC) for women and newborns: conclusions, evidence gaps and research priorities. *Reprod Health*. 2014;11(S2)(Suppl 2):S5. CrossRef. Medline
8. United Nations Inter-agency Group for Child Mortality Estimation (UN IGME). *Levels and Trends in Child Mortality: Report 2018.* UN IGME; 2018. Accessed December 22, 2020. https://childmortality.org/wp-content/uploads/2018/12/UN-IGME-Child-Mortality-Report-2018.pdf
9. World Health Organization (WHO). *Roadmap on Human Resource Strategies to Improve Newborn Care in Health Facilities in Low and Middle-Income Countries*. WHO; 2020. Accessed December 22, 2020. https://apps.who.int/iris/bitstream/handle/10665/336677/9789240015227-eng.pdf
10. Aluvaala J, Nyamai R, Were F, et al.; SIRCLE/Ministry of Health Hospital Survey Group. Assessment of neonatal care in clinical training facilities in Kenya. *Arch Dis Child*. 2015;100(1):42-47. CrossRef. Medline
11. Neogi SB, Khanna R, Chauhan M, et al. Inpatient care of small and sick newborns in healthcare facilities. *J Perinatol*. 2016;36(S3)(s3):S18-S23. CrossRef. Medline
12. Moxon SG, Lawn JE, Dickson KE, et al. Inpatient care of small and sick newborns: a multi-country analysis of health system bottlenecks and potential solutions. *BMC Pregnancy Childbirth*. 2015;15(S2)(Suppl 2):S7. CrossRef. Medline
13. Every Woman Every Child (EWEC). *The Global Strategy for Women's, Children's, and Adolescents' Health (2016-2030): Survive, Thrive, Transform.* EWEC; 2015. Accessed December 22, 2020. http://www.everywomaneverychild.org/global-strategy/
14. World Health Organization (WHO). *Global Strategic Directions for Strengthening Nursing and Midwifery 2016-2020.* WHO; 2016.

https://www.who.int/hrh/nursing_midwifery/global-strategy-midwifery-2016-2020/en/

15. Bell SA, Rominski S, Bam V, Donkor E, Lori J. Analysis of nursing education in Ghana: priorities for scaling-up the nursing workforce. *Nurs Health Sci*. 2013;15(2):244-249. CrossRef. Medline

16. World Bank. World Development Indicators. Data Bank. Accessed December 22, 2020. https://databank.worldbank.org/reports.aspx?source=2&series=SH.MED.NUMW.P3&country=

17. Sharma G, Mathai M, Dickson KE, et al. Quality care during labour and birth: a multi-country analysis of health system bottlenecks and potential solutions. *BMC Pregnancy Childbirth*. 2015;15(S2)(Suppl 2):S2. CrossRef. Medline

18. World Health Organization (WHO). *2018 Progress Report: Reaching Every Newborn National 2020 Milestones*. WHO; 2018. Accessed December 22, 2020. https://www.healthynewbornnetwork.org/hnn-content/uploads/Final-Country-Progress-Report-v9-low-res.pdf

19. Roberton T, Carter ED, Chou VB, et al. Early estimates of the indirect effects of the COVID-19 pandemic on maternal and child mortality in low-income and middle-income countries: a modelling study. *Lancet Glob Health*. 2020;8(7):e901-e908. CrossRef. Medline

20. Kruk ME, Pate M. The Lancet Global Health Commission on High Quality Health Systems 1 year on: progress on a global imperative. *Lancet Glob Health*. 2020;8(1):e30-e32. CrossRef. Medline

21. World Health Organization (WHO). *The World Health Report 2006: Working Together for Health*. WHO; 2006. Accessed December 22, 2020. https://www.who.int/whr/2006/en/

22. World Health Organization (WHO), UNICEF. *Ending Preventable Newborn Deaths and Stillbirths by 2030*. WHO, UNICEF; 2020. Accessed December 22, 2020. https://www.healthynewbornnetwork.org/hnn-content/uploads/Final-draft-Every-Newborn-Coverage-target-Milestones-to-2025.pdf

23. World Health Organization (WHO). *Standards for Improving the Quality of Care for Small and Sick Newborns in Health Facilities*. WHO; 2020. Accessed December 22, 2020. https://www.who.int/publications/i/item/9789240010765

24. Tunçalp Ö, Were WM, MacLennan C, et al. Quality of care for pregnant women and newborns—the WHO vision. *BJOG*. 2015;122(8):1045-1049. CrossRef. Medline

25. Levac D, Colquhoun H, O'Brien KK. Scoping studies: advancing the methodology. *Implement Sci*. 2010;5(1):69. CrossRef. Medline

26. Arksey H, O'Malley L. Scoping studies: towards a methodological framework. *Int J Soc Res Methodol*. 2005;8(1):19-32. CrossRef.

27. The World Bank. World Bank country and lending groups. Accessed December 23, 2020. https://datahelpdesk.worldbank.org/knowledgebase/articles/906519-world-bank-country-and-lending-groups

28. Braun V, Clarke V. Using thematic analysis in psychology. *Qual Res Psychol*. 2006;3(2):77-101. CrossRef.

29. Martinez AM, Khu DTK, Boo NY, Neou L, Saysanasongkham B, Partridge JC. Barriers to neonatal care in developing countries: parents' and providers' perceptions. *J Paediatr*

Child Health. 2012;48(9):852-858. CrossRef. Medline

30. Ministry of Health Ghana. *Ghana National Newborn Health Strategy and Action Plan 2014-2018*. Accessed December 23, 2020. https://www.healthynewbornnetwork.org/hnn-content/uploads/FINAL_Ghana-Newborn_Strategy_2014July10.pdf
31. Neogi SB, Malhotra S, Zodpey S, Mohan P. Challenges in scaling up of special care newborn units-lessons from India. *Indian Pediatr*. 2011;48(12):931-935. CrossRef. Medline
32. Getachew A, Ricca J, Cantor D, et al. Quality of Care for Prevention and Management of Common Maternal and Newborn Complications: A Study of Ethiopia's Hospitals. Jhpiego; 2011. Accessed December 23, 2020. https://www.healthynewbornnetwork.org/hnn-content/uploads/Ethiopia_Qualtiy-of-Care-Assement-report_MCHIP_final-1.pdf
33. National Research Council. *Reducing Maternal and Neonatal Mortality in Indonesia: Saving Lives, Saving the Future*. National Academies Press; 2013. CrossRef.
34. Mbonye AK, Sentongo M, Mukasa GK, et al.; Uganda Decade of Change and Future Implications Analysis Group. Newborn survival in Uganda: a decade of change and future implications. *Health Policy Plan*. 2012;27(suppl_3):iii104-iii117. CrossRef. Medline
35. Adegoke AA, Atiyaye FB, Abubakar AS, Auta A, Aboda A. Job satisfaction and retention of midwives in rural Nigeria. *Midwifery*. 2015;31(10):946-956. CrossRef. Medline
36. Kildea S, Larsson M, Govind S. A review of midwifery in Mongolia utilising the 'Strengthening Midwifery Toolkit'. *Women Birth*. 2012;25(4):166-173. CrossRef. Medline
37. Bogren M, Ndela B, Toko C, Berg M. Midwifery education, regulation and association in the Democratic Republic of Congo (DRC)—current state and challenges. *Glob Health Action*. 2020;13(1):1717409. CrossRef. Medline
38. Elikplim Pomevor K, Adomah-Afari A. Health providers' perception of quality of care for neonates in health facilities in a municipality in Southern Ghana. *Int J Health Care Qual Assur*. 2016;29(8):907-920. CrossRef. Medline
39. Campbell-Yeo M, Deorari A, McMillan DD, et al. Educational barriers of nurses caring for sick and at-risk infants in India. *Int Nurs Rev*. 2014;61(3):398-405. CrossRef. Medline
40. Malhotra S, Zodpey SP, Vidyasagaran AL, et al. Assessment of essential newborn care services in secondary-level facilities from two districts of India. *J Health Popul Nutr*. 2014;32(1):130-141. CrossRef. Medline
41. Haile-Mariam A, Tesfaye N, Otterness C, Bailey PE. Assessing the health system's capacity to conduct neonatal resuscitation in Ethiopia. *Ethiop Med J*. 2012;50(1):43-55. Medline.
42. Bream KDW, Gennaro S, Kafulafula U, Mbweza E, Hehir D. Barriers to and facilitators for newborn resuscitation in Malawi, Africa. *J Midwifery Womens Health*. 2005;50(4):329-334. CrossRef. Medline
43. Kozuki N, Oseni L, Mtimuni A, et al. Health facility service availability and readiness for intrapartum and immediate postpartum care in Malawi: a cross-sectional survey. *PLoS One*. 2017;12(3):e0172492. CrossRef. Medline

44. Jirapaet V, Jirapaet K, Sopajaree C. The nurses' experience of barriers to safe practice in the neonatal intensive care unit in Thailand. *J Obstet Gynecol Neonatal Nurs*. 2006;35(6):746-754. CrossRef. Medline

45. Cole-Ceesay R, Cherian M, Sonko A, et al. Strengthening the emergency healthcare system for mothers and children in The Gambia. *Reprod Health*. 2010;7(1):21. CrossRef. Medline

46. Michel-Schuldt M, Billy Dayon M, Toft Klar R, et al. Continuous professional development of Liberia's midwifery workforce—a coordinated multi-stakeholder approach. *Midwifery*. 2018;62:77-80. CrossRef. Medline

47. Nyamtema AS, Urassa DP, Massawe S, Massawe A, Lindmark G, Van Roosmalen J. Staffing needs for quality perinatal care in Tanzania. *Afr J Reprod Health*. 2008;12(3):113-124. Medline.

48. Nzinga J, McKnight J, Jepkosgei J, English M. Exploring the space for task shifting to support nursing on neonatal wards in Kenyan public hospitals. *Hum Resour Health*. 2019;17(1):18. CrossRef. Medline

49. Ntigurirwa P, Mellor K, Langer D, et al. A health partnership to reduce neonatal mortality in four hospitals in Rwanda. *Global Health*. 2017;13(1):28. CrossRef. Medline

50. Fischer EA, Jayana K, Cunningham T, et al. Nurse mentors to advance quality improvement in primary health centers: lessons from a pilot program in Northern Karnataka, India. *Glob Health Sci Pract*. 2015;3(4):660-675. CrossRef. Medline

51. Amin AA, Vankar JR, Nimbalkar SM, Phatak AG. Perceived stress and professional quality of life in neonatal intensive care unit nurses in Gujarat, India. *Indian J Pediatr*. 2015;82(11):1001-1005. CrossRef. Medline

52. Ith P, Dawson A, Homer CSE, Klinken Whelan A. Practices of skilled birth attendants during labour, birth and the immediate postpartum period in Cambodia. *Midwifery*. 2013;29(4):300-307. CrossRef. Medline

53. Prytherch H, Kakoko DCV, Leshabari MT, Sauerborn R, Marx M. Maternal and newborn healthcare providers in rural Tanzania: in-depth interviews exploring influences on motivation, performance and job satisfaction. *Rural Remote Health*. 2012;12(3):2072 Medline.

54. Allen CW, Jeffery H. Implementation and evaluation of a neonatal educational program in rural Nepal. *J Trop Pediatr*. 2006;52(3):218-222. CrossRef. Medline

55. Tosif S, Nasi T, Gray A, Sadr-Azodi N, Ogaoga D, Duke T. Assessment of the quality of neonatal care in the Solomon Islands. *J Paediatr Child Health*. 2018;54(2):165-171. CrossRef. Medline

56. Neogi SB, Malhotra S, Zodpey S, Mohan P. Assessment of special care newborn units in India. *J Health Popul Nutr*. 2011;29(5):500-509. CrossRef. Medline

57. Dewez JE, Chellani H, Nangia S, et al. Healthcare workers' views on the use of continuous positive airway pressure (CPAP) in neonates: a qualitative study in Andhra Pradesh, India. *BMC Pediatr*. 2018;18(1):347. CrossRef. Medline

58. Morgan MC, Dyer J, Abril A, et al. Barriers and facilitators to the provision of optimal obstetric and neonatal emergency care and to the implementation of simulation-enhanced mentorship in primary care facilities in Bihar, India: a qualitative study. *BMC Pregnancy Childbirth*. 2018;18(1):420. CrossRef. Medline

59. Horiuchi S, Rattana S, Saysanasongkham B, et al. Study protocol of a cluster randomized controlled trial to evaluate effectiveness of a system for maintaining high-quality early essential newborn care in Lao PDR. *BMC Health Serv Res*. 2018;18(1):489. CrossRef. Medline

60. Sychareun V, Phommachanh S, Soysouvanh S, et al. Provider perspectives on constraints in providing maternal, neonatal and child health services in the Lao People's democratic republic: a qualitative study. *BMC Pregnancy Childbirth*. 2013;13(1):243. CrossRef. Medline

61. Brunson J. Confronting maternal mortality, controlling birth in Nepal: the gendered politics of receiving biomedical care at birth. *Soc Sci Med.* 2010;71(10):1719-1727. CrossRef. Medline

62. Moxon SG, Ruysen H, Kerber KJ, et al. Count every newborn; a measurement improvement roadmap for coverage data. *BMC Pregnancy Childbirth*. 2015;15(S2)(Suppl 2):S8. CrossRef. Medline

63. World Health Organization (WHO). Transforming and Scaling Up Health Professional Education and Training. Policy Brief on Regulation of Health Professions Education. WHO; 2013, Accessed December 23, 2020. https://apps.who.int/iris/bitstream/handle/10665/93635/9789241506502_eng.pdf?sequence=1

64. Kalyan G, Vatsa M. Neonatal nursing: an unmet challenge in India. *Indian J Pediatr*. 2014;81(11):1205-1211. CrossRef. Medline

65. Hudspeth J, Curry CL, Sacks Z, Surena C. Continuing professional development in low-resource settings: Haiti as example. *Ann Glob Health*. 2015;81(2):255-259. CrossRef. Medline

66. Bolan NE, Sthreshley L, Ngoy B, et al. mLearning in the Democratic Republic of the Congo: a mixed-methods feasibility and pilot cluster randomized trial using the safe delivery app. *Glob Health Sci Pract*. 2018;6(4):693-710. CrossRef. Medline

67. Rowe AK, de Savigny D, Lanata CF, Victora CG. How can we achieve and maintain high-quality performance of health workers in low-resource settings? *Lancet*. 2005;366(9490):1026-1035. CrossRef. Medline

68. Nilsson C, Sørensen BL, Sørensen JL. Comparing hands-on and video training for postpartum hemorrhage management. *Acta Obstet Gynecol Scand*. 2014;93(5):517-520. CrossRef. Medline

69. World Health Organization (WHO). *Global Strategy on Human Resources for Health: Workforce 2030*. WHO; 2016. Accessed December 23, 2020. https://www.who.int/hrh/resources/globstrathrh-2030/en/

70. Bolan NE, Newman SD, Nemeth LS. Technology-based newborn health learning initiatives for facility-based nurses and midwives in low-and middle-income countries: a scoping review. *Int J Childbirth*. 2018;8(4):252-268. CrossRef.

71. World Health Organization (WHO). Every Newborn: An Action Plan to End Preventable Deaths. WHO; 2014. Accessed December 23, 2020. https://www.who.int/maternal_child_adolescent/documents/every-newborn-action-plan/en/

72. Gupta N, Maliqi B, França A, et al. Human resources for maternal, newborn and child health: from measurement and planning to performance for improved health outcomes. *Hum Resour Health*. 2011;9(1):16. CrossRef. Medline

73. Smith SL, Hall MA. Advanced neonatal nurse practitioners in the workforce: a review of the evidence to date. *Arch Dis Child Fetal Neonatal Ed*. 2011;96(2):F151-F155. CrossRef. Medline

74. Rogowski JA, Staiger D, Patrick T, Horbar J, Kenny M, Lake ET. Nurse staffing and NICU infection rates. *JAMA Pediatr*. 2013;167(5):444-450. CrossRef. Medline

75. Küng E, Waldhör T, Rittenschober-Böhm J, Berger A, Wisgrill L. Increased nurse workload is associated with bloodstream infections in very low birth weight infants. *Sci Rep*. 2019;9(1):6331. CrossRef. Medline

76. Watson SI, Arulampalam W, Petrou S, et al.; Neonatal Data Analysis Unit (NDAU) and the Neonatal Economic, Staffing, and Clinical Outcomes Project (NESCOP) Group. The effects of a one-to-one nurse-to-patient ratio on the mortality rate in neonatal intensive care: a retrospective, longitudinal, population-based study. *Arch Dis Child Fetal Neonatal Ed*. 2016;101(3):F195-F200. CrossRef. Medline

77. Hamilton KE, Redshaw ME, Tarnow-Mordi W. Nurse staffing in relation to risk-adjusted mortality in neonatal care. *Arch Dis Child Fetal Neonatal Ed*. 2007;92(2):F99-F103. CrossRef. Medline

78. BLISS. The chance of a lifetime? BLISS Baby Report 2010. *BLISS*. 2010;(8):8-10. Accessed January 21, 2021. https://issuu.com/bliss-charity/docs/baby_report_2010_web

79. Buchan J, Shaffer FA, Catton H. *Policy Brief: Nurse Retention*. International Centre on Nursing Migration; 2018. Accessed December 23, 2020. https://www.icn.ch/sites/default/files/inline-files/2018_ICNM%20Nurse%20retention.pdf

80. Filby A, McConville F, Portela A. What prevents quality midwifery care? A systematic mapping of barriers in low and middle income countries from the provider perspective. *PLoS One*. 2016;11(5):e0153391. CrossRef. Medline

81. Manasyan A, Saleem S, Koso-Thomas M, et al.; EmONC Trial Group. Assessment of obstetric and neonatal health services in developing country health facilities. *Am J Perinatol*. 2013;30(09):787-794. CrossRef. Medline

82. Franco LM, Bennett S, Kanfer R. Health sector reform and public sector health worker motivation: a conceptual framework. *Soc Sci Med*. 2002;54(8):1255-1266. CrossRef. Medline

83. Dieleman M, Toonen J, Touré H, Martineau T. The match between motivation and performance management of health sector workers in Mali. *Hum Resour Health*. 2006;4(1):2. CrossRef. Medline

84. Thi Hoai Thu N, Wilson A, McDonald F. Motivation or demotivation of health workers providing maternal health services in rural areas in Vietnam: findings from a mixed-methods study. *Hum Resour Health*. 2015;13(1):91. CrossRef. Medline

85. Fonn S, Ray S, Blaauw D. Innovation to improve health care provision and health systems in sub-Saharan Africa—promoting agency in mid-level workers and district managers. *Glob Public Health*. 2011;6(6):657-668. CrossRef. Medline

86. Inoue KC, Gomes da Silva Versa GL, Misue Matsuda L. Stress level among intensive care nurses in the municipality of Paraná (Brazil). *Invest Educ Enferm*. 2014;32(1):69-77.

CrossRef. Medline

87. Campbell J, Cometto G, Rasanathan K, et al. Improving the resilience and workforce of health systems for women's, children's, and adolescents' health. *BMJ*. 2015;351:h4148. CrossRef. Medline
88. World Health Organization (WHO). *Midwives' Voices Midwives' Realities.* WHO; 2016. Accessed December 23, 2020. https://www.who.int/maternal_child_adolescent/documents/midwives-voices-realities/en/
89. Gallagher A. Moral distress and moral courage in everyday nursing practice. *Online J Issues Nurs*. 2011;16(2):8. CrossRef. Medline
90. Campbell J. The route to effective coverage is through the health worker: there are no shortcuts. *Lancet*. 2013;381(9868):725. CrossRef. Medline

Peer Reviewed

Received: July 3, 2020; **Accepted**: December 9, 2020; **First published online**:

Citer cet article: Bolan N, Cowgill KD, Walker K, et al. Défis liés aux ressources humaines pour la santé dans l'offre de soins de qualité aux nouveau-nés par les infirmières et les sages-femmes dans les pays à revenu faible et moyen: une revue de cadre. *Glob Health Sci Pract*. 2021;9(1). https://doi.org/10.9745/GHSP-D-20-00362

© Bolan et al. Ceci est un oeuvre d'accès ouvert, distribué sous les conditions de la license Creative Commons Attribution, qui autorize l'utilization, la distribution et la reproduction non restraint, sous condition que l'auteur original soit correctement cité. Pour une copie de la license, voir: http://creativecommons.org/licenses/by/4.0/. https://doi.org/10.9745/GHSP-D-20-00362
