# Supplementary material for: Human Resources for Health-Related Challenges to Ensuring Quality Newborn Care in Low- and Middle-Income Countries: A Scoping Review
Source: Glob Health Sci Pract. 2021 Mar 31;9(1):160–76. doi: 10.9745/GHSP-D-20-00362 (PMC8087437; doi:10.9745/GHSP-D-20-00362)
Supplement: GHSP2000362_Spanish.pdf [file GHSP2000362_Spanish.pdf]

GLOBAL HEALTH: SCIENCE AND PRACTICE
Dedicated to what works in global health programs
OPEN ACCESS

REVISIÓN EXPLORATORIA

Desafíos de los recursos humanos en el área de salud para garantizar atención neonatal de calidad en países con ingresos bajos y medianos: una revisión exploratoria

Nancy Bolan,[a] Karen D. Cowgill,[b] Karen Walker,[c] Lily Kak,[d] Theresa Shaver,[e] Sarah Moxon,[f] Ornella Lincetto[g]

Mensajes clave

- Se mapeó evidencia de los desafíos de los recursos humanos en el área de la salud (RHS) y el poder brindar atención neonatal de calidad en centros hospitalarios. Esta evidencia se clasificó en 10 categorías:
  - Falta de seguimiento y datos de los RHS
  - Formación deficiente previa al ejercicio de las funciones de los trabajadores de la salud y escaso material de contenido acerca de la salud del recién nacido
  - Falta de acceso a material dirigido a trabajadores de la salud, como guías de práctica clínica basadas en evidencia, educación continua y desarrollo profesional continuo
  - Distribución insuficiente e inequitativa del personal de salud y cargas de trabajo extenuantes
  - Mala retención del personal, ausentismo y rotación del personal experimentado
  - Mal ambiente laboral, así como salarios bajos
  - Supervisión limitada y deficiente
  - Baja moral, falta de motivación y de actitud, así como insatisfacción laboral
  - Debilidades en políticas, regulaciones, administración, liderazgo, gobernanza y financiamiento
  - Barreras estructurales y contextuales
- El mapeo de la evidencia proporcionó información útil para poder presentar las estrategias de la Organización Mundial de la Salud publicadas recientemente, las cuales describen el abordaje sistemático de los desafíos y el fortalecimiento de los RHS para la atención del recién nacido, tanto a nivel mundial como a nivel nacional.
- El proceso de análisis temático también resalta las complicadas interacciones entre los diferentes tipos de desafíos de los RHS.
- Los hallazgos sugieren nuevas estrategias de acción para abordar estos desafíos.

[a] Oficina de Salud Global, Escuela de Enfermería de la Universidad de Maryland, Baltimore, MD, EE. UU.
[b] Departamento de Salud Global de la Universidad de Washington, Seattle, WA, EE. UU.
[c] The George Institute for Global Health, Newtown, Australia.
[d] Agencia de los Estados Unidos para el Desarrollo Internacional, Washington, DC, EE. UU.
[e] Social Solutions International, Inc., Washington, DC, EE. UU.
[f] Escuela de Higiene y Medicina Tropical de Londres, Londres, Reino Unido.
[g] Organización Mundial de la Salud, Ginebra, Suiza.
Correspondencia a Nancy Bolan (nbolan@umaryland.edu).

RESUMEN

Antecedentes: La escasez crítica de trabajadores de la salud que a su vez cuenten con las competencias necesarias para atender a la madre y al recién nacido sigue siendo un desafío importante en la prestación de servicios de atención de calidad, especialmente, en países con ingresos bajos y medianos. La problemática de la oferta de los recursos humanos para la salud (RHS), agrava la falta de estos y puede afectar negativamente el desempeño de los trabajadores de la salud y la calidad de la atención. Esta investigación utiliza fuentes centralizadas en cada país para identificar y mapear la evidencia de los desafíos del campo de los RHS para la prestación de atención neonatal de calidad en centros hospitalarios por parte de enfermeras y parteras.
Métodos: La evidencia de esta revisión fue recopilada de forma iterativa, comenzando con documentos pertinentes de la Organización Mundial de la Salud y extendiéndose a los artículos identificados a través de bases de datos y búsquedas manuales en listas de referencias e informes de los países incluidos en el estudio. La evidencia se obtiene de fuentes enfocadas en cada país y se considera la información a partir del año 2000. La extracción de datos fue realizada utilizando una herramienta diseñada de forma reiterativa; además, se hizo uso de un análisis temático para mapear las 10 categorías de los desafíos de los RHS.
Resultados: Se examinaron un total de 332 artículos y fueron revisados por pares, 22 de estos cumplieron los criterios de

Los trabajadores sanitarios con competencias maternas y neonatales son sumamente necesarios para ampliar la prestación de atención especializada para madres y recién nacidos.

inclusión. Se agregaron catorce fuentes adicionales de búsqueda manual de listas de referencias y fuentes de literatura de carácter no científico. La evidencia se ha mapeado en 10 categorías de desafíos relacionados con los RHS: (1) falta de datos y monitoreo de los trabajadores de la salud; (2) deficiencia en educación previa al servicio como trabajador de la salud; (3)falta de acceso a material dirigido a trabajadores de la salud, como guías de práctica clínica basadas en evidencia, educación continua y desarrollo profesional continuo; (4) distribución insuficiente y desigual de los trabajadores de la salud y extenuantes cargas de trabajo; (5) mala retención del personal, ausentismo y rotación del personal experimentado; (6) mal ambiente laboral, así como bajos salarios; (7) supervisión limitada y deficiente; (8) baja moral, falta de motivación y de actitud, así como insatisfacción laboral; (9) debilidades en políticas, regulaciones, administración, liderazgo, gobierno y financiamiento; y (10) barreras estructurales y contextuales.

Conclusión: El mapeo proporciona la información necesaria y muestra las nuevas estrategias de la Organización Mundial de la Salud y los esfuerzos para apoyar el abordaje de los desafíos identificados y fortalecer los recursos humanos para la atención neonatal, con el objetivo final de mejorar la atención y los resultados en el recién nacido.

ANTECEDENTES

A nivel mundial, los recién nacidos son extremadamente vulnerables; alrededor de 2,5 millones de bebés mueren durante sus primeros 28 días de vida (el período neonatal), y alrededor del 77% de esas muertes ocurren durante la primera semana de vida.[1,2] Además, cada año, casi 2 millones de muertes fetales ocurren en los últimos 3 meses del embarazo o durante el parto[3,4] y millones de bebés desarrollan morbilidades a corto y largo plazo, así como problemas neurocognitivos.[5,6] La mayoría de los recién nacidos pueden sobrevivir y continuar con su etapa de desarrollo si cuentan con acceso a atención médica de calidad. Sin embargo, la reducción en la mortalidad neonatal sigue siendo lenta y desigual debido a una cobertura variable de intervenciones esenciales y la calidad de la atención en la prestación de servicios por parte del personal de salud (PS).[7,8] El acceso universal a la atención de calidad podría prevenir 1,7 millones de muertes neonatales cada año, o el 68% de las muertes que de otro modo ocurrirían para el año 2030.[5]

Las asistentes capacitadas y las parteras capaces de brindar atención de alta calidad durante parto, proveer atención esencial para el recién nacido y seguir correctamente un protocolo de remisión, son fundamentales, ya que las muertes neonatales tempranas están indisolublemente vinculadas a la salud de la madre y a la calidad de la atención que reciben una madre y su bebé durante la labor de parto, el parto, y el período de posparto inmediato.[7]

Asimismo, se necesita personal competente en el cuidado del recién nacido que esté compuesto principalmente por enfermeras y parteras y que ellas a su vez reciban el apoyo de médicos y otros especialistas de la salud; de modo que en los centros hospitalarios se pueda brindar atención a aproximadamente 30 millones de recién nacidos que cada año requieren atención médica.[5,9]

Dado que la mayoría de las mujeres eligen el parto institucional y la mortalidad neonatal desciende por debajo de 30/1000 nacidos vivos en todo el mundo, las intervenciones que se llevan a cabo en centros de atención primaria (básica), secundaria ("atención especial") y terciaria (cuidado intensivo neonatal) se vuelven cada vez más importantes para alcanzar cifras de mortalidad más bajas.[10] La atención materna y neonatal en centros hospitalarios implica un servicio clínico de 24 horas, brindado por personal capacitado en los centros de salud, enfocados en la atención de rutina y en el manejo de las complicaciones.[11,12]

En conjunto, las enfermeras y las parteras componen el mayor porcentaje del personal de salud en todo el mundo y son fundamentales para lograr no solo mejores resultados en la salud materna y neonatal, sino también sistemas de salud más sólidos que garanticen que todos los recién nacidos sobrevivan, se desarrollen y ejerzan su derecho a los más altos estándares de salud y bienestar alcanzables.[5,13–15]

Sin embargo, una crítica escasez de trabajadores sanitarios con las competencias maternas y neonatales necesarias sigue siendo un impedimento para ampliar la prestación de atención especializada para madres y recién nacidos, especialmente en países de ingresos bajos y medianos (PIBM). En la actualidad, los países de ingresos altos tienen un promedio de 10,9 enfermeras y parteras por cada 1000 habitantes, en comparación al 2,5 y 0,9 en países de ingresos bajos y medianos y países de bajos ingresos, respectivamente.[17,18]

A medida que la pandemia de la enfermedad del coronavirus del 2019 (COVID-19) sobrecarga los sistemas de salud en muchos países, los recién nacidos, aún y cuando es menos probable que mueran a causa de la COVID-19, tienen un mayor riesgo de mortalidad por otras enfermedades prevenibles y condiciones tratables a medida que se

interrumpe el acceso y la disponibilidad de los servicios de salud.

A pesar de que se le ha prestado mayor atención al tema de los recursos humanos en el área de la salud (RHS) a raíz del Informe Mundial de la Salud del 2006 y la Asamblea Mundial de la Salud, así como desde la creación de la Red Mundial de Personal Sanitario (GHWA, por sus siglas en inglés). La red fue quien encabezó los esfuerzos para los RHS de la Organización Mundial de la Salud (OMS) del 2006 al 2016; la atención especial a los RHS para recién nacidos, iniciativa más reciente, al igual que un enfoque en la calidad de la atención brindada.[20,21] Hoy en día es esencial abordar la escasez crítica de los trabajadores de la salud competentes para lograr las ambiciosas metas recientemente lanzadas como parte del Plan de Acción 2025 "Todos los Recién Nacidos", al igual que los nuevos estándares de la OMS que pretenden mejorar la calidad de la atención de los recién nacidos pequeños y enfermos en los centros de salud; lo anterior como parte del progreso hacia el logro de los Objetivos de Desarrollo Sostenible (ODS).[22,23]

Esta revisión exploratoria responde al llamado de brindar información oportuna en el Año de la Enfermera y Partera de la OMS que pretende asegurar que este tema, relativamente descuidado, obtenga un lugar en las mesas de discusión. El estudio, de igual forma analiza el alcance de la literatura para identificar y mapear la evidencia, enfocada por país y los desafíos de los RHS para garantizar atención neonatal de calidad por parte de enfermeras y parteras en países de ingresos bajos y medianos.

Así mismo, proporciona la base de evidencia para las estrategias de la OMS recientemente publicadas para abordar estos desafíos.[9,24] Si bien, la atención clínica en la comunidad también es fundamental para reducir la mortalidad y morbilidad materna y neonatal, este artículo se centra en la atención del recién nacido que se brinda en los centros de salud.

MÉTODOS

Enfoque

Para este estudio se seleccionó una metodología de determinación exploratoria debido a que el enfoque permite la acumulación expedita a gran escala de literatura y el mapeo de la evidencia adicional así como determinar el alcance de la evidencia y las brechas que requieren una mayor investigación.[25] El enfoque aplicado en esta revisión exploratoria utiliza un proceso de cinco etapas: (1) identificar la pregunta de investigación, (2) identificar los estudios relevantes, (3) seleccionar los estudios, (4) registrar los datos y (5) cotejar, resumir e informar los resultados.[26]

La evidencia del estudio se recopiló de forma iterativa, comenzando con los documentos pertinentes de la OMS y las series publicadas sobre el tema, siguiendo con artículos identificados mediante búsquedas en bases de datos y búsquedas manuales en listas de referencias, así como informes de países y organizaciones.

Búsquedas en bases de datos

Este estudio planteó la siguiente pregunta de investigación: ¿qué evidencia está disponible sobre los desafíos de RHS para la prestación de atención neonatal de calidad por parte de enfermeras y parteras en los países de ingresos bajos y medianos? Las búsquedas en la base de datos tenían como objetivo encontrar artículos, comentarios e informes revisados por pares de países de ingresos bajos y medianos que abordaran el tema de la investigación y que se publicaran a partir del año 2000, con el inicio de los Objetivos de Desarrollo del Milenio.

Esta revisión investigó qué evidencia se encuentra disponible sobre los desafíos relacionados con los RHS para la prestación de atención neonatal de calidad por parte de enfermeras y parteras en los países de ingresos bajos y medianos.

Estrategia de búsqueda y criterios de selección

Los datos de este estudio se identificaron mediante búsquedas en PubMed, EMBASE, CENTRAL, Índice acumulativo de literatura de enfermería y salud afín (CINAHL, por sus siglas en inglés), Revistas Africanas en línea (AJOL, por sus siglas en inglés), Literatura de Ciencias de la Salud para Latinoamérica y el Caribe (LILACS, por sus siglas en inglés) y referencias de artículos relevantes. Los términos de búsqueda utilizados fueron los siguientes: enfermeras, enfermería, parteras, partería, enfermeras parteras, recién nacidos, recién nacidos, lactantes, prematuros, prematuros, de bajo peso al nacer, países en desarrollo, países de ingresos bajos, países de ingresos medios, pacientes hospitalizados, hospitales, centros de atención hospitalaria, centros de salud, clínicas, unidades en el cuidado de recién nacidos, unidades de cuidados neonatales, unidades de cuidados intensivos neonatales, recursos humanos de salud, recursos humanos para la salud, fuerza laboral, personal de salud, políticas, educación, empleo, despliegue, distribución, retención, escasez, salarios, motivación, desempeño, supervisión, combinados con el uso de operadores booleanos "Y" y "O" y limitados a humanos.

Se realizaron búsquedas en literatura de carácter no científico a través de Open Gray (www.opengrey.eu), Grey Literature Report (www.greylit.org) y Healthy Newborn Network. Dichas búsquedas se realizaron en inglés, sin restricciones de idioma y únicamente fueron tomados en cuenta artículos publicados a partir del año 2000.

La estrategia de búsqueda completa se proporciona en un anexo.

Criterios de inclusión y exclusión

Los resultados de la búsqueda se ingresaron en el software Covidence (www.covidence.org) y dos revisores (NEB y KDC) examinaron de forma independiente los títulos y resúmenes de los estudios en función de criterios de inclusión y exclusión. Los artículos seleccionados para la revisión de texto completo cumplieron con los criterios de inclusión consistentes con los manuscritos que evalúan los desafíos de los RHS para la atención del recién nacido por parte de enfermeras y parteras a nivel de centros hospitalarios en países de ingresos bajos y medianos.

Los criterios específicos de inclusión fueron:

1. Tema: aborda el desafío de RHS
2. Proveedores: enfermeras y/o parteras
3. Población de pacientes: desde el nacimiento hasta los 28 días de vida (período neonatal) y el respectivo cuidador
4. Entorno: centros de atención de la salud de nivel primario, secundario o terciario en PIBM: países incluidos en la lista de PIBMs del Banco Mundial.[27]
5. Enfoque por país; evidencia nacional, regional, de distrito o de centro de salud en el país
6. Años: 2000 al presente

Las razones de la exclusión agrupan fuentes que se basan únicamente en la comunidad y no en los centros de atención o que describen iniciativas con parteras tradicionales, trabajadores sanitarios comunitarios o médicos únicamente; iniciativas de política que eran teóricas y aún no habían sido implementadas; o investigaciones llevada a cabo en entornos de refugiados (estos entornos a menudo enfrentan desafíos únicos). Cada revisor examinó de forma independiente cada documento de texto completo para determinar si la fuente debía incluirse o excluirse, y los desacuerdos se resolvieron mediante discusión. Se muestran los resultados de la búsqueda y revisión (Figura).

FIGURA. Diagrama de flujo de los resultados de la búsqueda y revisión de evidencia sobre recursos humanos para los desafíos relacionados con la salud para la atención neonatal de calidad en países de ingresos bajos y medios

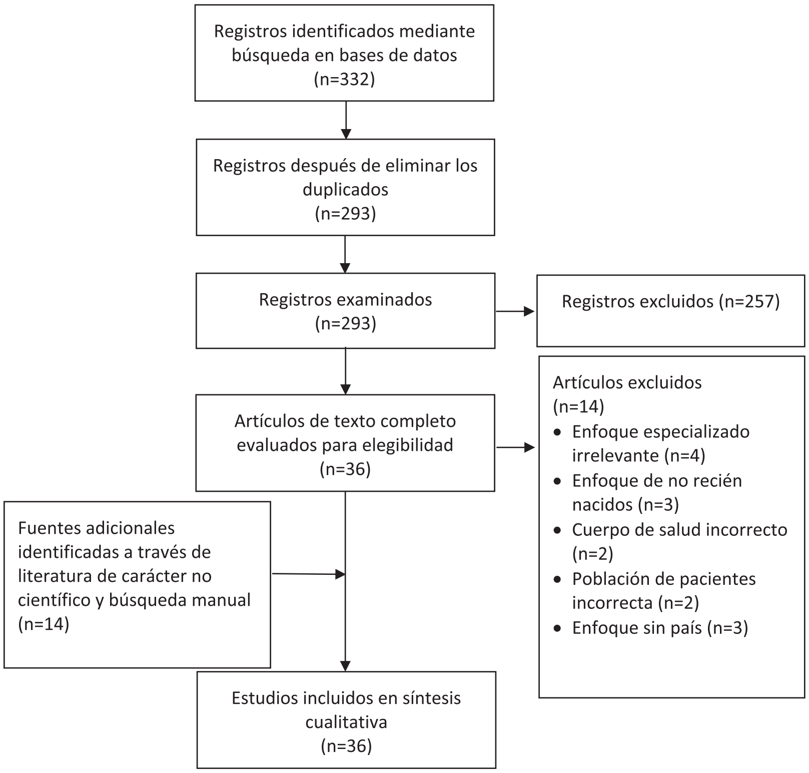

Extracción y gestión de datos

El autor principal (NEB) leyó y codificó cada documento de texto completo utilizando un formulario de extracción de datos, creado de forma iterativa a medida de que se iban revisando los textos completos. Los campos de datos fueron los siguientes: desafío de los RHS, ejemplo (detalle) del desafío, país, entorno, métodos de investigación, tipo de documento (revisado por pares o literatura de carácter no científico), tipo de proveedor, año de publicación, fuente y cualquier nota adicional.

Los datos extraídos se utilizaron para crear un mapa conceptual o cuadro de desafíos de loa RHS y luego agrupar temas similares por categorías a través del análisis temático inductivo descrito por Braun y Clarke.[28] Las categorías de los desafíos fueron revisadas por 2 autores (NEB y OL), y a su vez colapsaron temas basados en el volumen de evidencia de cada categoría usando un proceso iterativo e inductivo para llegar a un consenso sobre las 10 categorías finalistas.

RESULTADOS

Se recuperaron un total de 332 artículos, los cuales fueron revisados por pares, inclusive los 39 duplicados encontrados. 293 resúmenes se revisaron bajo criterios de inclusión y exclusión previamente identificados. 22 de estas fuentes, cumplieron los criterios de inclusión (Figura). Se agregaron 14 registros adicionales de la búsqueda en manuales de referencias y fuentes de literatura de carácter no científico, lo que elevó el número total de artículos incluidos a 36. Las fuentes que cumplieron los criterios de inclusión cubrieron 20 de 138 PIBM. Con 8 artículos revisados por pares, la India tuvo la representación más alta y los 19 países restantes estuvieron representados por 1 o 2 publicaciones cada uno. Una publicación cubrió 4 países (Camboya, República Democrática Popular Lao, Vietnam y Malasia).[29]

La mayoría de las fuentes incluidas fueron publicaciones revisadas por pares (n = 33). Los años de publicación abarcados fueron del 2005 al 2019, y las fuentes se basaron principalmente en evidencia de enfermeras (incluidas enfermeras obstétricas y neonatales, enfermeras de la unidad de cuidados intensivos neonatales [UCIN] y estudiantes de enfermería), parteras (incluidas las enfermeras parteras auxiliares) y en un alcance menor, evidencia de médicos, oficiales médicos, oficiales de salud de distrito, administradores de centros de salud y legisladores.

Los métodos de investigación utilizados en los artículos revisados fueron tanto cualitativos (entrevistas y grupos focales) como cuantitativos (encuestas, auditorías, pruebas de selección múltiple y de habilidades, cuestionarios); los artículos también empleaban la revisión de documentos, la revisión de registros y la observación para recopilar datos. Un artículo se basó en datos de un grupo nacional de múltiples partes interesadas[30] y otro en el análisis de datos secundarios[31]. La mayoría de los estudios reportaron datos de UCIN, centros de salud, hospitales y hospitales de distrito de regiones específicas. Cuatro artículos utilizaron datos a nivel nacional para su análisis (Etiopía, Indonesia, Ghana y Uganda).[30,32–34]

El análisis temático de los datos dio como resultado 10 categorías de desafíos relacionados con los RHS que enfrentan las enfermeras y las parteras para brindar atención neonatal de calidad en centros de salud. En el cuadro se proporciona un resumen de los datos clasificados por desafío de los RHS. En ciertas ocasiones, los datos de cada fuente se asignaron a más de un desafío, como se indica en el cuadro.

Un análisis de datos temáticos arrojó 10 categorías de desafíos relacionados con los RHS que enfrentan las enfermeras y parteras para brindar atención neonatal de calidad.

Categorías de los desafíos de los RHS

1. Falta de datos y seguimiento de los RHS necesarios para la salud materna y neonatal

Las fuentes revisadas reportaron falta de datos de los RHS sobre la disponibilidad, distribución, tendencias y requisitos del personal.[35,36] Por ejemplo, Nigeria presentó una escasez crítica de funcionarios de la salud, particularmente en centros salud ubicados en áreas rurales, así como la problemática tarea de planificar la contratación y asignación del personal; esta última tarea representa un verdadero reto dada la falta de datos críticos sobre los RHS.[35] De manera similar, en Mongolia, Kildea et al.[36] se observaron que además de la escasez general de enfermeras, parteras y profesionales de la salud aliados para la atención materna y neonatal, es difícil determinar el número necesario de diferentes cuerpos de funcionarios de la salud, vinculado a la falta de métricas para evaluar esos cuerpos.

2. Formación deficiente previa al ejercicio de las funciones de los trabajadores de la salud y escaso material de contenido acerca de la salud del recién nacido

Las fuentes respaldan que el proceso de formación que precede al ejercicio de las funciones de trabajadores de la salud es a menudo deficiente, especialmente en lo que respecta a la formación de conocimientos y habilidades del recién nacido, esta característica se evidenció en todos los cuerpos de los trabajadores de la salud y existen pocos programas para la formación de enfermeras

CUADRO. Recursos humanos mapeados para los desafíos relacionados con la salud y fuentes de revisión respectivas.

Desafío de los recursos Humanos para la salud (RHS)	Ejemplo	País	Tipo de Fuente	Fuente
1. Falta de datos y seguimiento de los RHS necesarios para la salud materna y neonatal	La escasez de datos de los RHS como una barrera para la planificación de los RHS.	Nigeria	RP	Adegoke et al.[35]
	Pocos indicadores de fuerza laboral para la prestación de servicios de parto.	Mongolia	RP	Kildea et al.[36]
2. Formación deficiente previa al ejercicio de las funciones de los trabajadores de la salud y escaso material de contenido acerca de la salud del recién nacido	Falta de instructores calificados y de preceptores clínicos para parteras	República Democrática del Congo	RP	Bogren et al.[37]
	Componente neonatal básico insuficiente en formación previa al ejercicio de la enfermería.	Ghana	RP	Elikplim Pomevor y Adomah-Afari[38]
	Deficiencias en el plan de estudios de enfermería a nivel de licenciatura y bajo rendimiento en comparación con otros proveedores (incluyendo a las enfermeras diplomadas).	Etiopía	Reporte	Getachew et al.[32]
	Durante su formación básica, el personal de salud recibe instrucción limitada previa al servicio que se enfoque en la atención neonatal.	Kenia	RP	Aluvaala et al.[10]
	El cuidado del neonato no es una competencia central en la educación general de la enfermería.	La India	RP	Campbell-Yeo et al.[39]
3. Falta de acceso a material dirigido a trabajadores de la salud, como guías de práctica clínica basadas en evidencia, educación continua y desarrollo profesional continuo	Falta de disponibilidad de guías / protocolos de cuidado neonatal y de resucitación neonatal.	Etiopía	RP	Haile-Mariam et al.[41]
	Falta de guías / protocolos publicados en resucitación neonatal.	Malawi	RP	Bream et al.[42]
	Poca disponibilidad de protocolos para la atención de neonatos enfermos.	Ghana	RP	Elikplim Pomevor y Adomah-Afari[38]
	Guías que indiquen como ayudar a respirar a los bebés en el 25% de los centros de salud.	Malawi	RP	Kozuki et al.[43]
	Falta de disponibilidad de Protocolos de resucitación neonatal.	Tanzania	RP	Nyamtema et al.[47]
	Poca difusión de Protocolos de Práctica para las enfermeras de UCIN.	Tailandia	RP	Jirapaet et al.[44]
	Poca formación en servicio sobre recién nacidos, resucitación neonatal, así como los cuidados de recién nacidos pequeños y enfermos.	Ghana	RP	Elikplim Pomevor y Adomah-Afari[38]
		La India	RP	Malhotra et al.[40]
	El 75% de los centros de salud examinados no tenían un médico capacitado en atención básica obstétrica y neonatal de emergencia.	Malawi	RP	Kozuki et al.[43]
	Oportunidades variables de EC para enfermeras neonatales.	La India	RP	Campbell-Yeo et al.[39]
			RP	Bolan et al.[66]

Continued

CUADRO. Continued

Desafío de los recursos Humanos para la salud (RHS)	Ejemplo	País	Tipo de Fuente	Fuente
	Acceso limitado de EC para enfermeras y parteras	República Democrática del Congo		
	Falta de políticas de EC / renovación de habilidades.	Malawi	RP	Bream et al.[42]
	EC deficiente para trabajadores sanitarios en el manejo de emergencias de recién nacidos o resucitación neonatal.	Gambia	RP	Cole-Ceesay et al.[45]
	Falta de educación continua para parteras.	Mongolia	RP	Kildea et al.[36]
	Falta de coordinación de continuidad del desarrollo profesional de los trabajadores de la salud. Anteriormente el DPC no era obligatorio para las parteras y existía una ausencia de control de calidad sobre el DPC	Liberia	RP	Michel-Schuldt et al.[46]
4. Distribución insuficiente e inequitativa de funcionarios de la salud y extenuante carga de trabajo	Cantidad insuficiente de trabajadores de la salud, en general	Nigeria	RP	Adegoke et al.[35]
		Malawi	RP	Bream et al.[42]
		Tanzania	RP	Nyamtema et al.[47]
	Falta de trabajadores de la salud en la atención primaria de salud / hospitales de distrito.	Nepal	RP	Allen y Jeffrey[54]
	Personal de UCIN insuficiente.	Tailandia	RP	Jirapaet et al.[44]
	Falta de personal de enfermería especializado en cuidados neonatales y recién nacidos enfermos	Sudeste asiático (4 países)	RP	Martinez et al.[29]
		Islas Salomón	RP	Tosif et al.[55]
		La India	RP	Neogi et al.[31]
	Escasez crítica de enfermería en unidades neonatales	Kenia	RP	Nzinga et al.[48]
		Kenia	RP	Aluvaala et al.[10]
	Escasez de trabajadores de la salud con formación neonatal	Ruanda	RP	Ntigurirwa et al.[49]
	Baja cantidad de trabajadores de la salud que se ha agravado por una distribución desigual	Indonesia	Reporte	Consejo Nacional de Investigación[33]
	Mala distribución en áreas rurales / remotas	Nepal	RP	Allen y Jeffrey[54]
		Nigeria	RP	Adegoke et al.[35]
		La India	RP	Fischer et al.[50]
	Extenuante carga de trabajo para el personal existente	La India	RP	Amin et al.[51]
		Camboya	RP	Ith et al.[52]
		La India	RP	Morgan et al.[58]
	Ocupación de plazas con personal de menor nivel	Tanzania	RP	Prytherch et al.[53]

Continued

CUADRO. Continued

Desafío de los recursos Humanos para la salud (RHS)	Ejemplo	País	Tipo de Fuente	Fuente
5. Retención, ausentismo y rotación deficientes del personal experimentado	Mala retención del personal de salud debido a (ejemplos):<br>- Muertes de trabajadores de la salud por VIH<br>- Migración rural-urbana<br>- Fuga de Profesionales<br>- Deserción hacia el sector privado y organizaciones no gubernamentales	Tanzania	RP	Nyamtema et al.[47]
		Ruanda	RP	Ntigurirwa et al.[49]
		La India	RP	Neogi et al.[11]
		Gambia	RP	Cole-Ceesay et al.[45]
	Ausentismo	La India	RP	Neogi et al.[11]
		Tanzania	RP	Nyamtema et al.[47]
		Tanzania	RP	Prytherch et al.[53]
	Rotación<br>- traslado de enfermeras neonatales a otras unidades o centros de salud	La India	RP	Dewez et al.[57]
		Ghana	RP	Elikplim Pomevor y Adomah-Afari[38]
		Ruanda	RP	Ntigurirwa et al.[49]
6. Mal ambiente de trabajo	Salario bajo y pagos irregulares	Nigeria	RP	Adegoke et al.[35]
		Camboya	RP	Ith et al.[52]
		Tanzania	RP	Prytherch et al.[53]
	Alojamiento precario	Gambia	RP	Cole-Ceesay et al.[45]
	Falta de seguridad en el trabajo:<br>- Falta de equipo de protección<br>- Temor a robos, agresiones, violencia de género y agresiones familiares	Nigeria	RP	Adegoke et al.[35]
		Gambia	RP	Cole-Ceesay et al.[45]
		La India	RP	Morgan et al.[58]
		Tanzania	RP	Prytherch et al.[53]
	Falta de equipo, medicamentos esenciales y suministros	Nigeria	RP	Adegoke et al.[35]
		Sudeste Asiático (4 países)	RP	Martinez et al.[29]
		Etiopía	RP	Haile-Mariam et al.[41]
		La India	RP	Morgan et al.[58]
	Falta de soporte técnico, retrasos en la reparación de los equipos existentes	Islas Salomón	RP	Tosif et al.[55]
	Mala distribución y equipamiento de las salas impiden una atención adecuada	La India	RP	Neogi et al.[56]
		Malawi	RP	Bream et al.[42]
7. Supervisión limitada y deficiente	Capacidad limitada para supervisión de rutina	RDP Lao	RP	Horiuchi et al.[59]
	Falta mentores en sitio y soporte técnico para las parteras con habilidades limitadas	Nigeria	RP	Adegoke et al.[35]
	Falta de supervisión clínica en las UCIN.	Tailandia	RP	Jirapaet et al.[12]
	Supervisión punitiva, sin retroalimentación, falta de confidencialidad	Tanzania	RP	Prytherch et al.[53]

Continued

CUADRO. Continued

Desafío de los recursos Humanos para la salud (RHS)	Ejemplo	País	Tipo de Fuente	Fuente
	Después de efectuadas las visitas de supervisión, los problemas no son resueltos	RDP Lao	RP	Horiuchi et al.[59]
8. Baja moral, falta de motivación y de actitud, así como insatisfacción laboral	Malas condiciones laborales, salarios bajos, falta de incentivos	Gambia	RP	Cole-Ceesay et al.[45]
	Falta de ruta de crecimiento Profesional y oportunidades de Promoción para los trabajadores sanitarios	Nigeria	RP	Adegoke et al.[35]
		Mongolia	RP	Kildea et al.[36]
		Tanzania	RP	Prytherch et al.[53]
	Agotamiento, estrés y consternación debido a cargas de trabajo extenuantes y el número de pacientes	La India	RP	Amin et al.[51]
		La India	RP	Dewez et al.[57]
	Impotencia debido al entorno de trabajo, falta de control sobre los traslados de personal.	Tanzania	RP	Prytherch et al. 2012[53]
		La India	RP	Dewez et al.[57]
	Baja moral debido a la alta mortalidad de los pacientes y la incapacidad para brindar una buena atención debido al mal estado.	Gambia	RP	Cole-Ceesay et al.[45]
		La India	RP	Dewez et al.[57]
		Tanzania	RP	Prytherch et al.[53]
9. Debilidades de en las políticas, los reglamentos, la gestión, el liderazgo, la gobernanza y el financiamiento	Falta de marcos políticos y normativos para la atención neonatal y los Protocolos clínicos.	Ghana	RP	Elikplim Pomevor y Adomah-Afari[38]
		Camboya	RP	Ith et al.[52]
	Alcance limitado de la práctica de las parteras, no está en concordancia con el alcance internacional de la práctica.	Mongolia	RP	Kildea et al.[36]
	No existen estándares de competencia aceptados a nivel nacional para parteras y enfermeras.	Mongolia	RP	Kildea et al.[36]
		Indonesia	Reporte	Consejo Nacional de Investigación[33]
	Falta de paridad entre los salarios de los trabajadores públicos y religiosos.	Tanzania	RP	Prytherch et al.[53]
	Ausencia de normas de dotación de personal por nivel de centro de salud.	Ghana	Reporte	Ministerio de Salud de Ghana[30]
	Falta de reconocimiento en el manejo de la necesidad de proporciones similares a las de la UCI en unidades de cuidados neonatales.	La India	RP	Dewez et al.[57]
	Falta de apoyo por parte del equipo de gestión del hospital, los líderes del centro de salud o las autoridades.	La India	RP	Dewez et al.[57]
		La India	RP	Fischer et al.[50]
	Falta de descripciones de trabajo, guías neonatales u orientación en las unidades de recién nacidos.	Kenia	RP	Nzinga et al.[48]
		Tanzania	RP	Prytherch et al.[53]
		La India	RP	Campbell-Yeo et al.[39]
	Baja productividad del PS.	Tanzania	RP	Nyamtema et al.[47]
	Gestión sin apoyo, horarios de trabajo inflexibles.	Tanzania	RP	Prytherch et al.[53]

Continued

CUADRO. Continued

Desafío de los recursos Humanos para la salud (RHS)	Ejemplo	País	Tipo de Fuente	Fuente
	Deficiencias de recursos humanos a nivel gerencial que resultan en una gestión ineficiente, particularmente relacionada con el cálculo de presupuesto	RDP Lao	RP	Sychareun et al.[60]
	Falta de priorización de la salud del recién nacido por parte de los responsables políticos, en el pasado	Ghana	Reporte	Ministerio de salud de Ghana[30]
	Asociaciones profesionales débiles que no mejoran las condiciones del PS	Tanzania	RP	Prytherch et al.[53]
	Financiamiento inadecuado en los presupuestos nacionales para la atención neonatal (sector público)	Uganda	RP	Mbonye et al.[34]
		Tanzania	RP	Nyamtema et al.[47]
		Gambia	RP	Cole-Ceesay et al.[45]
10. Barreras estructurales y contextuales	Falta de reconocimiento de los requisitos en atención especializada del recién nacido	La India	RP	Campbell-Yeo et al.[39]
	Falta de percepción comunitaria del peso que conlleva la enfermedad neonatal.	Nepal	RP	Allen y Jeffrey[54]
	Descuido de las recién nacidas.	La India	RP	Morgan et al.[58]
	Las mujeres en edad fértil carecen de poder para determinar el comportamiento de búsqueda de cuidados en situaciones de emergencia.	Nepal	RP	Brunson[61]

Abreviaturas: ED, educación continua; DPC, desarrollo Profesional continuo; CERN, cuidados esenciales del recién nacido; RHS, recursos humanos para la salud; TS, trabajador de salud; UCI, Unidad de cuidados intensivos; RP, partera; RN, recién nacido; UCIN, unidad de cuidados intensivos neonatales; RN, reanimación neonatal; RP, revisado por pares.

neonatales especializadas en PIBM.[10,32,37–39] Por ejemplo, los funcionarios de la salud en Kenia reciben una limitada instrucción previa al ejercicio de su labor por lo que no cuentan con suficiente educación relacionada a los cuidados neonatales en su formación básica, la mayor experiencia práctica se obtiene durante las prácticas clínicas o pasantías en hospitales.[10] De forma similar, se observó que el cuidado del recién nacido no era una competencia central en la educación general de enfermería en la India[39]; En Ghana, se observó que el material de contenido para recién nacidos era insuficiente en la educación previa a su ejercicio.[38]

3. Falta de acceso a material dirigido a trabajadores de la salud, como guías de práctica clínica basadas en evidencia, educación continua y desarrollo profesional continuo.

La falta de disponibilidad o la falta de acceso a guías o protocolos de práctica actuales basados en la evidencia, la educación continua y el desarrollo profesional continuo para el cuidado del recién nacido es una queja común en las fuentes de países de ingresos bajos y medianos.[36,38–47] Jirapaet et al.[44] informaron sobre un estudio realizado en 4 UCIN en Tailandia donde los investigadores notaron una falta de difusión de los protocolos de práctica entre las enfermeras. De manera similar, un estudio realizado en todos los hospitales y centros de salud con servicio de maternidad de Etiopía reportó la falta de disponibilidad de directrices y protocolos en la atención esencial del recién nacido y la reanimación neonatal.[41]

La educación continua, definida como un proceso continuo de aprendizaje, es una piedra angular de la competencia continua y está estrechamente relacionada a la calidad de la atención y la seguridad del paciente.[21] El desarrollo profesional continuo se refiere al proceso de seguimiento y documentación de las habilidades, el conocimiento y la experiencia que el funcionario de la salud adquiere tanto formal

como informalmente más allá de cualquier capacitación inicial. Michel-Schuldt et al.[46] señalaron que el desarrollo profesional continuo no es obligatorio para las parteras en Liberia y de manera más general, existe una falta de coordinación del desarrollo profesional continuo para los profesionales de la salud, así como una ausencia de control de calidad en la formación de las parteras.

En Gambia, Cole-Ceesay et al[45] encontraron poca educación continua para los trabajadores sanitarios en el manejo de la reanimación neonatal o las emergencias neonatales; Ghana reportó una capacitación limitada en los servicios de atención neonatal, reanimación neonatal o atención de recién nacidos pequeños y enfermos.

4. Distribución insuficiente e inequitativa de los trabajadores de la salud y extenuante carga de trabajo

Las fuentes aportaron numerosos ejemplos de la falta de una distribución adecuada y equitativa de los trabajadores sanitarios, específicamente la falta de parteras calificadas y enfermeras neonatales calificadas, y en consecuencia, se observaron pesadas cargas de trabajo para el personal en servicio y proporciones inaceptables de personal.[10,29,33,35,42,44,47–56] Un estudio realizado en 2 unidades de recién nacidos ubicadas en hospitales públicos de Nairobi reportó proporciones de personal de 1 enfermera por cada 15 bebés.[48] Aluvaala et al.[10] encontraron que de 22 unidades de recién nacidos que se encuestaron en hospitales de Kenia, 6 de ellas tenían una escasez de personal tan severa que no se pudo asignar ni siquiera una enfermera al cuidado exclusivo de cada unidad de recién nacidos.

De manera similar, un estudio en 9 UCIN de la India reportó proporciones desiguales de personal que variaban de 1: 4 a 1: 8 en centros de salud privados, pero de 1:25 a 1:35 en centros de salud gubernamentales.[51] Un estudio de varios países en el sudeste asiático informó que las unidades neonatales de Hanoi atendían habitualmente a un 50% más de pacientes con respecto a las camas y el personal disponible, por lo cual se veían obligados a colocar a 2 pacientes en una misma cama.[29] Neogi et al.[56] evaluaron 8 unidades especiales de atención neonatal en hospitales de distritos rurales en toda la India y encontraron que la proporción de enfermeras por recién nacido pareció desempeñar un papel fundamental en la mejora de la supervivencia de los recién nacidos en estas unidades: casi el 15% de la variación en la tasa de mortalidad neonatal entre las unidades podría justificarse con la proporción de enfermeras por recién nacidos.

Sumado a la extenuante carga de trabajo del personal que labora en zonas rurales, Tanzania informó sobre un desequilibrio en la proporción del personal de salud calificado y el de menor nivel dedicado a la atención a madres y recién nacido en este tipo de centros hospitalarios. Se demostró que el 43% de la fuerza laboral estaba compuesta por cuerpos laborales de nivel inferior (p. Ej., auxiliares de salud materno-infantil, asistentes clínicos y asistentes).[53]

5. Retención, ausentismo y rotación deficientes del personal experimentado

La mala retención de los trabajadores maternos y neonatales se describió en muchas fuentes[11,38,45,47,49,53,57]. Se informó de ausentismo[11] relacionado con el hecho de que los trabajadores de la salud del sector público también trabajaban en la práctica privada en la India. Un estudio realizado en 2 centros de salud rurales en Tanzania reportó que los trabajadores sanitarios a menudo tienen actividades de generación de ingresos paralelas, como la agricultura, el comercio u otro trabajo de salud, para complementar sus salarios, lo que puede generar distracciones en el trabajo y ausentismo.

Los artículos incluidos en esta revisión también citaron que la rotación dentro del centro de salud y el traslado del personal a otro centro son una preocupación importante. En un estudio de 6 centros de salud en Ghana, se entrevistó a muchos profesionales sanitarios sobre la rotación anual del personal neonatal, ya que esto obstaculizaba la calidad de la atención debido a la pérdida de personal experimentado y el tiempo requerido en entrenar al nuevo personal.[38] Dewez et al.[57] describió que cuando las enfermeras de la India se capacitaban en atención neonatal y se rotaban a otras salas, perdían confianza y habilidades neonatales.

6. Mal ambiente de trabajo

A menudo se reportan salarios deficientes, pagos inconsistentes y alojamientos precarios.[29,31,35,41,42,45,52,53,55,58] Un estudio cualitativo llevado a cabo con trabajadores de la salud en Camboya[52] reportó salarios bajos para parteras calificadas, por lo que ellas solo brindan atención posnatal a la madre y al recién nacido si los miembros de la familia realizan pagos adicionales. Como se señaló en el quinto desafío de RHS, los trabajadores de la salud a menudo crean actividades adicionales de generación de ingresos para complementar sus ingresos.[53]

La falta de equipo, suministros, medicamentos, electricidad y agua son frecuentemente mencionados en la literatura.[29,35,41,58] Los suministros y equipos de protección, como guantes y máscaras, pueden afectar la seguridad del personal de salud. Esta problemática puede exacerbarse durante brotes como el de la pandemia actual de COVID-19 y contribuye a la mala calidad de la atención.[19] Además, la falta de seguridad del personal de la salud en donde son expuestos a escrutinio y agresiones, también se menciona en la literatura; un artículo de la India señaló que los proveedores remiten los casos complicados para no ser agredidos por miembros de la familia en caso de malos resultados maternos y neonatales.

7. Supervisión limitada y deficiente

La supervisión deficiente se cita como un problema que conduce a una retención deficiente del personal de salud, baja moral y falta de motivación, así como a una prestación de atención de mala calidad.[35,44,53,59] Horiuchi et al.[59] señalaron que aunque las visitas de supervisión externa son un enfoque común para promover una conducta de cambio entre el personal de salud dedicado a la atención de los recién nacidos, así como para reforzar las habilidades y mantener la calidad de la atención, estas visitas no siempre son factibles en entornos con recursos limitados. Lo mencionado anteriormente ocurre en lugares como la República Democrática Popular Lao debido a factores relacionados con los altos costos y alta demanda del recurso humano. Los autores señalan que existe una capacidad mínima para implementar supervisión de rutina; sin embargo, cuando ocurren visitas de supervisión, los problemas identificados, comúnmente, permanecen sin resolver [59]. De manera similar, los trabajadores de la salud del servicio de maternidad y de recién nacidos, entrevistados en Tanzania informaron que la supervisión y la evaluación del desempeño eran punitivas y que carecían de confidencialidad.[53]

8. Baja moral, falta de motivación y de actitud, así como insatisfacción laboral

La moral y la motivación se mencionaron con frecuencia en las fuentes.[35,36,45,51,53,57] La baja motivación y la insatisfacción laboral se relacionaron con una variedad de factores, incluidos los bajos salarios, las malas condiciones de trabajo, la falta de oportunidades profesionales y de promoción, la falta de control sobre el traslado de puestos, capacitación y orientación técnica insuficientes, agotamiento y estrés ligados a una extenuante carga de trabajo y supervisión desmoralizadora.[36,45,51,53]

Varios artículos reflejan el alto nivel de estrés y la baja moral es causado por la elevada tasa de mortalidad materna y neonatal.[45,51] Los trabajadores de la salud lidian además con sentimientos de culpa e impotencia relacionados con el no poder brindar una mejor atención a los pacientes.[57] Se reportó estrés y agotamiento entre los trabajadores de la UCIN en la India y Tailandia.[44,51] Igualmente, en Tanzania se observó que la baja moral y la mala actitud afectaron la provisión de atención; por ejemplo, se observó que la pasividad del proveedor a la hora de atender emergencias potencialmente mortales contribuía a los malos resultados.

9. Debilidades en políticas, reglamentos, gestión, liderazgo, gobernanza y financiamiento

Las fuentes que documentan debilidades en el ámbito de políticas y gestión fueron de naturaleza variada.[30,33,34,36,38,39,47,48,50,52,53,57,60] Un estudio realizado en Tanzania concluyó que los factores que desalientan a los proveedores de servicios de maternidad y neonatales podrían dividirse en los que pertenecen a las condiciones de empleo (por ejemplo, relacionados con la política) y los que pertenecen a la organización de los procesos de trabajo (por ejemplo, relacionados con la gestión de centros de salud).[53] Liderazgo, gobernanza y referencias de financiación apuntan a una falta de priorización de la atención del recién nacido y su falta de financiamiento en los presupuestos nacionales.

10. Barreras estructurales y contextuales

Una variedad de posibles barreras estructurales y contextuales para la provisión de atención neonatal de alta calidad aumenta los desafíos de RHS antes mencionados.[39,54,58,61] Un estudio en las zonas rurales de Nepal menciona que una barrera significativa para mejorar la atención neonatal es la falta de percepción acerca del peso que conlleva la enfermedad neonatal en la comunidad de donde provienen los proveedores de salud, y que, sin la percepción de un problema, los proveedores tienen pocos incentivos para mejorar el desempeño laboral.[54]

Un informe de Bihar, observó que en la India existe una preferencia por los niños varones y la negligencia con las recién nacidas, llegando al grado en que las familias, en ocasiones, amenazan al personal de salud que intenta resucitar a las recién nacidas[58] Los prejuicios de género afectan la atención del recién nacido en una variedad de formas adicionales, desde la dinámica del potencial de la mayoría de las profesionales de partería

y enfermería hasta las dinámicas de potencial basadas en el género relacionadas con decisiones de parto, como comportamiento selectivo en la atención de emergencias, como se informó en Nepal.[61]

DISCUSIÓN

Hasta donde es conocido, esta revisión es la primera que analiza de manera integral los desafíos de los RHS para brindar atención neonatal de calidad en países de ingresos bajos y medianos. Al realizar esta revisión exploratoria, se busca comprender mejor los desafíos relacionados con los RHS para la prestación de atención neonatal de calidad en centros hospitalarios por parte de enfermeras y parteras, enfocada en países de ingresos bajos y medianos mediante la identificación de áreas temáticas tangibles que permitan el abordaje a nivel nacional, regional e internacional.

El objetivo fue sintetizar la evidencia identificada en fuentes centralizadas en cada país y proporcionar la información necesaria para informar las estrategias enfocadas en el fortalecimiento de los RHS y la atención del recién nacido a nivel mundial y nacional, reforzando la capacidad del país y la capacidad en el cumplimiento de los nuevos estándares de la OMS para la atención de recién nacidos pequeños y enfermos, metas de ENAP 2025 y los ODS. Los objetivos para abordar este urgente conjunto de desafíos son mejorar la calidad de la atención y reducir la mortalidad, los mortinatos y la morbilidad a corto y largo plazo de los recién nacidos.

Los datos y el seguimiento insuficiente de los RHS dificultan la planificación y la toma de decisiones de la fuerza laboral.[5,18] Con frecuencia, faltan datos esenciales sobre la disponibilidad de los trabajadores sanitarios, especialmente enfermeras y parteras.[36] Se carece de datos que muestren la combinación necesaria de habilidades del personal en los centros de salud, lo que consecuentemente dificulta la planificación [7] para mejorar la calidad de la atención en los recién nacidos y facilitar la promoción; se necesitan datos sobre quién, dónde y cómo los trabajadores sanitarios cuidan a los recién nacidos.[62] La investigación de métricas de los RHS y equipos de monitoreo que puedan guiar la planificación nacional es una prioridad global.[5]

La evidencia de varios países muestra que la educación previa al ejercicio de la labor de médicos, enfermeras y parteras tiende a ser deficiente; del mismo modo, la rápida proliferación de escuelas del sector privado no acreditadas plantea interrogantes sobre su calidad.[63] El material incluido en los planes de estudio de pregrado y dirigidos a la atención neonatal está frecuentemente limitado a cuerpos de salud relevantes.[64] Un análisis de cuellos de botella para la atención hospitalaria de recién nacidos pequeños y enfermos reveló que en los países con mayor mortalidad neonatal, hay una falta de capacitación basada en habilidades e indicó que la educación relacionada es inconsistente y está mal estructurada.[12,17] Parte de la dificultad proviene de la falta de profesores capacitados y preceptores clínicos calificados en la enseñanza de atención neonatal, lo que resulta en oportunidades limitadas para adquirir habilidades para el cuidado del recién nacido durante el período de estudio.

El acceso a material dirigido a funcionarios de la salud, como guías de práctica clínica basadas en evidencia, educación continua y desarrollo profesional continúo es difícil o imposible de adquirir, especialmente en áreas rurales.[21,65,66] En cuanto a la atención específica para bebés pequeños y enfermos, las investigaciones realizadas en 12 países con altas tasas de mortalidad neonatal, informaron que no existía capacitación o educación continua adecuada y si lo había esta no estaba basada en competencias. Dicho escenario también aplicaba para capacitaciones y actualizaciones en sitio, particularmente en centros de salud de nivel inferior.[12] Las directrices cambian regularmente, por ello, la importancia en la difusión y la implementación métodos de actualización para que así los trabajadores de la salud adquieran nuevo conocimiento; sin embargo, la simple divulgación de las guías escritas resulta ineficaz.[67] La mayoría de las emergencias obstétricas y neonatales tienen lugar en centros de salud periféricos, a los cuales es difícil hacerles llegar programas de capacitación convencionales y por ello requieren estrategias de aprendizaje innovadoras.[68]

Los objetivos para abordar los desafíos de los RHS son mejorar la calidad de la atención y reducir la mortalidad, los mortinatos y la morbilidad a corto y largo plazo de los recién nacidos.

Las oportunidades para abordar estos desafíos ahora están disponibles a través de paquetes de aprendizaje basados en competencias y en evidencia que incorporan capacitación y tutoría en simulación, iniciativas de aprendizaje electrónico y móvil digital, y otras herramientas de aprendizaje innovadoras que utilizan tecnología como herramientas de capacitación de realidad virtual en decisiones clínicas, aprendizaje y apoyo en el centro de atención.[66,69,70] No obstante, se necesita investigación adicional sobre el aprendizaje electrónico y móvil, particularmente en los países de ingresos bajos y medianos, que mida los resultados del recién nacido como un resultado primario.[70]

La escasez de personal de salud (categoría 4) es uno de los principales factores que explican la

persistencia de las altas tasas de mortalidad de mujeres y recién nacidos en muchos países.[5,71] Menos de 1 de cada 6 países con la carga más alta de mortalidad materna y neonatal alcanza el punto de referencia mínimo necesario para proporcionar un paquete básico de atención, el cual debería constar de 23 médicos, parteras y enfermeras por cada 10,000 habitantes.[63] Un número insuficiente de trabajadores de la salud, combinado con malas condiciones de trabajo y pocos incentivos para que el personal viva y trabaje en áreas remotas o entre poblaciones desfavorecidas, lleva a una distribución desigual.

Existen desequilibrios no solo en el número y la distribución geográfica del personal de salud disponible, sino también en el sector del empleo (público/privado)[72] y en la gama de sus competencias. La mayoría de los países todavía tienen muy pocos especialistas en relación con las necesidades de salud de su población. La escasez es particularmente evidente en los médicos especialistas (por ejemplo, neonatólogos, cirujanos, obstetras y anestesistas) y enfermeras neonatales, contando pocos programas disponibles para capacitar a estos cuerpos sanitarios en entornos de bajos ingresos.[64,71] En contraste, los centros hospitalarios de países con altos ingresos, poseen una la columna vertebral compuesta por enfermeras neonatales dedicadas al cuidado del recién nacido y sus familias, incluso en roles extendidos como enfermeras neonatales avanzadas.[12,73]

La escasez del personal de salud y su inadecuada distribución, aumentan la carga de trabajo del personal existente, lo que genera una alta desproporción entre el personal y el paciente, así como mayor estrés. Lo anterior impide la capacidad de brindar atención de alta calidad e influye directamente en los resultados de los pacientes.[31,40] La investigación enfocada en la dotación de personal de enfermería de la UCIN y la carga de trabajo en los países de ingresos altos mostró que la falta de personal en relación con las directrices nacionales estaba asociado a un mayor riesgo de infección nosocomial en bebés de muy bajo peso al nacer.[74,75]

Por el contrario, el personal de enfermería de la UCIN que se asigna 1: 1, reduce la mortalidad hospitalaria.[76,77] No existen recomendaciones aceptadas a nivel mundial para las proporciones de personal en los diferentes niveles de atención neonatal, pero podemos contrastar las proporciones en los países de ingresos bajos y medianos con las proporciones recomendadas en el Reino Unido de 1 enfermera neonatal especializada por 1 paciente de cuidados intensivos neonatales y 1 enfermera titulada o partera a 4 paciente de cuidados especiales.

La mala retención, el ausentismo y la rotación de enfermeras experimentadas fuera de las unidades neonatales pueden crear la dinámica anteriormente mencionada y contribuir a su decadencia (desafío 5). La mayoría de los países (81%) muestran una fuerza laboral que favorece fuertemente las áreas urbanas, lo que puede estar relacionado con muchos factores, como mayores posibilidades de práctica privada y poco atractivo de las áreas rurales y remotas debido a las malas condiciones laborales, viviendas inadecuadas, oportunidades limitadas para desarrollo profesional y oportunidades educativas limitadas para los niños.[72,79]

Incluso, los mejores trabajadores de la salud, los más capacitados y los más motivados necesitan un entorno propicio y adecuado para trabajar de manera eficaz, que incluya una infraestructura en buenas condiciones y un suministro confiable de medicamentos, así como herramientas tecnológicas (desafío 6). Los salarios y entornos laborales deficientes contribuyen a otros desafíos, como la mala retención, especialmente en áreas remotas, y también a la baja moral y actitud.[17]

Un mapeo sistemático de las barreras para la prestación de atención de partos de calidad mostró la ausencia rutinaria de condiciones de trabajo seguras, como el no poder descartar objetos punzantes, escasez de agua para lavarse las manos y falta de suministros de protección básicos como guantes.[80] De manera similar, una encuesta realizada en 364 establecimientos de salud en África, Asia y América Latina encontró que los suministros y equipos esenciales no estaban disponibles en gran medida y concluyó que, generalmente, el personal no puede realizar procedimientos clave y que las mujeres, los mortinatos y los recién nacidos mueren innecesariamente como consecuencia de ello.[81]

La supervisión de apoyo (desafío 7) es otra herramienta que se utiliza en la mayoría de los entornos para apoyar a los trabajadores sanitarios y mejorar el desempeño laboral. Si se realiza correctamente, la supervisión puede ser un mecanismo para proporcionar desarrollo profesional, mejorar la satisfacción laboral y aumentar la motivación.[67] No obstante, la realidad es que las visitas de supervisión no siempre alcanzan su objetivo. Los supervisores pueden carecer de habilidades, herramientas y transporte para brindar una supervisión de calidad.[17,67] Los estudios planificados para evaluar el monitoreo continuo autogestionado por revisiones de pares y sesiones de retroalimentación, como una forma más sostenible de mejorar la calidad de la atención, pueden resultar una alternativa a los enfoques de supervisión actuales.[59]

El brindar atención de calidad depende de la motivación de los trabajadores profesionales y esta se convierte en un factor fundamental para mantener la voluntad de los trabajadores profesionales, su competencia profesional, dedicación a su labor y continuidad de la fuerza laboral.[67,82–85] Bajos niveles de la moral de los trabajadores profesionales, altos niveles de estrés y el agotamiento se ha resaltado en la literatura como un problema comúnmente dejado de lado (desafío 8) y ha sido ampliamente documentado entre las enfermeras de UCIN, enfermeras y parteras.[51,80,86]

Los investigadores sugieren que la motivación puede influir directamente en el desempeño y mediar cierto efecto en otros factores. Por lo tanto, es probable que la motivación y las intervenciones que mejoran la satisfacción laboral (por ejemplo: salarios, condiciones laborales) sean determinantes y de vital importancia en el desempeño laboral y la retención de los colaboradores.[67]

Los desafíos relacionados a la deficiencia o inexistencia en políticas, regulaciones, administración, liderazgo, gobernanza y financiamiento (desafío 9) son fundamentales para la atención neonatal de calidad.[30,33,34,36,38,39,47,48,50,52,53,57,60] Los ejemplos incluyen la falta de alineación entre la política nacional que define el alcance legal de la práctica para varios cuerpos laborales y la regulación de los mismos, las descripciones de los puestos, la educación previa al servicio o la práctica real.[72] Así mismo, existe una falta de regulación de instituciones educativas del sector privado y proveedores de salud,[87] falta de las políticas necesarias, como proporciones bien definidas de personal por paciente, sistemas de remisión, criterios de alta y niveles de atención estandarizados.[12]

Todos los programas de salud ya sean financiados por gobiernos, socios para el desarrollo, la sociedad civil o el sector privado, deben contribuir a las prioridades nacionales impulsadas por el gobierno. Lograr este objetivo requiere una mejor gobernanza y coordinación entre los sistemas nacionales y subnacionales.[87] Se necesitan promotores a nivel nacional para el avance de la atención de alta calidad en los recién nacidos, incluidos los formuladores de políticas, las personas clave dentro de los organismos profesionales, académicos e instituciones nacionales. En términos de financiamiento de la salud, la falta de financiamiento sostenido y coordinado para recién nacidos sigue siendo un desafío,[34] y es necesaria la movilización de financiamiento suficiente con un mejor flujo de efectivo.[87]

Finalmente, la literatura describe las barreras estructurales y contextuales que exacerban otros desafíos relacionados con los RHS, particularmente el bajo estatus social de las profesiones solidarias y la desigualdad de género en una fuerza laboral predominantemente femenina. Por ejemplo, un mapeo de las barreras para la provisión de atención de parto de calidad identificó la desigualdad de género y la falta de empoderamiento de la mujer como las barreras más importantes que conducen al estrés y al agotamiento, que a su vez conducen al desempoderamiento, disminución de la autoestima y, en última instancia, la adopción de actitudes negativas[80,88] El desempoderamiento también se ha mencionado ampliamente en la enfermería, una profesión igualmente dominada por las mujeres, junto con un alto estrés y agotamiento como se señaló anteriormente.[21,89]

Campbell[90] escribió que la única vía para lograr calidad en la atención es “a través del trabajador de la salud” y que la cobertura universal efectiva, con los RHS aseguren tanto la disponibilidad como la cobertura de calidad de los servicios de salud necesarios, lo mencionado anteriormente es el gran desafío para todos los países. Este hallazgo fue compartido por la Comisión de Salud Global de Lancet sobre Sistemas de Salud de Alta Calidad, que propone un reinicio de los sistemas de salud dada la magnitud de los déficits de calidad.[20] Se necesitan recursos humanos competentes y recursos físicos en todo momento para evitar la mortalidad evitable de mujeres, mortinatos y recién nacidos.[81]

Estos principios, que elevan la importancia del personal sanitario mundial, fueron articulados en el Informe sobre la salud mundial 2006 y reforzados con la creación de la Red Mundial de Personal Sanitario. La OMS rectificó las estrategias mundiales para abordar los desafíos de los RHS en la Estrategia mundial sobre recursos humanos para la salud: Fuerza Laboral 2030.[69] Dadas las nuevas metas y estándares de salud en beneficio de los recién nacidos pequeños y enfermos para el año 2025, las estrategias de los RHS específicas para recién nacidos (presentadas en los desafíos identificados de este artículo) se publicaron recientemente por la OMS con el propósito de abordar la accesibilidad y la calidad de la atención de esta población más vulnerable.[9] Los desafíos para la provisión de atención de alta calidad a las madres y los recién nacidos son complejos en cada país, interrelacionados y entrelazados con más amplios desafíos sociales y estructurales, lo cual requerirá atención y priorización continua en todos los niveles del sistema de salud si queremos tener éxito en alcanzar los ODS para el año 2030.

Limitaciones

Reducir y enfocar la pregunta de investigación fue un desafío, así como la amplia definición de lo que implica el cuidado del recién nacido, dio como resultado, una importante cantidad de información. Si bien se utilizó un proceso de análisis temático iterativo e inductivo para identificar los desafíos relacionados con los RHS para brindar atención neonatal de calidad, existían algunas superposiciones en áreas conceptuales que pueden haber enmascarado parte de la importancia, así como los matices o la interconexión de las categorías de desafíos. Además, nuestro análisis no es representativo de todos los PIBM, dado que muchos países no cuentan con los datos de sus respectivos desafíos relacionados con los RHS, que pueden ser únicos y específicos del contexto.

CONCLUSIONES

A tan solo 10 años de alcanzar los ODS, es fundamental garantizar el acceso a una atención de calidad para todos los recién nacidos que necesitan atención en un centro de salud. Sin embargo, la falta de un número suficiente de profesionales sanitarios con competencias en atención neonatal es una brecha crítica. Esta revisión abarcó artículos enfocados a nivel de país para explorar los desafíos relacionados con los RHS en la prestación de atención neonatal de calidad dentro de los centros hospitalarios por parte de enfermeras y parteras en países de ingresos bajos y medianos. La revisión identificó y mapeó la evidencia en 10 desafíos relacionados con los RHS y se interpretaron los datos. El mapeo proporciona la información necesaria que informa sobre las nuevas estrategias de la OMS y apoya los esfuerzos para abordar los desafíos identificados y fortalecer los recursos humanos para la atención neonatal, con el objetivo final de mejorar la atención y los resultados en el recién nacido.

REFERENCES

1. United Nations Inter-agency Group for Child Mortality Estimation (UN IGME). *Levels & Trends in Child Mortality: Report 2019, Estimates Developed by the United Nations Inter-Agency Group for Child Mortality Estimation.* UN IGME; 2019. https://www.unicef.org/media/60561/file/UN-IGME-child-mortality-report-2019.pdf
2. Sankar MJ, Natarajan CK, Das RR, Agarwal R, Chandrasekaran A, Paul VK. When do newborns die? A systematic review of timing of overall and cause-specific neonatal deaths in developing countries. *J Perinatol.* 2016;36(S1)(Suppl 1):S1–S11. CrossRef. Medline
3. Lawn JE, Blencowe H, Oza S, et al.; Lancet Every Newborn Study Group. Every Newborn: progress, priorities, and potential beyond survival. *Lancet.* 2014;384(9938):189–205. CrossRef. Medline
4. United Nations Inter-agency Group for Child Mortality Estimation (UN IGME). *A Neglected Tragedy: The Global Burden of Stillbirths.* UN IGME; 2020. Accessed December 22, 2020. https://www.unicef.org/reports/neglected-tragedy-global-burden-of-stillbirths-2020
5. World Health Organization (WHO). *Survive and Thrive: Transforming Care for Every Small and Sick Newborn.* WHO; 2019. Accessed December 22, 2020. https://www.unicef.org/media/58076/file
6. Lawn JE, Blencowe H, Darmstadt GL, Bhutta ZA. Beyond newborn survival: the world you are born into determines your risk of disability-free survival. *Pediatr Res.* 2013;74(S1)(Suppl 1):1–3. CrossRef. Medline
7. Bhutta ZA, Salam RA, Lassi ZS, Austin A, Langer A. Approaches to improve Quality of Care (QoC) for women and newborns: conclusions, evidence gaps and research priorities. *Reprod Health.* 2014;11(S2)(Suppl 2):S5. CrossRef. Medline
8. United Nations Inter-agency Group for Child Mortality Estimation (UN IGME). *Levels and Trends in Child Mortality: Report 2018.* UN IGME; 2018. Accessed December 22, 2020. https://childmortality.org/wp-content/uploads/2018/12/UN-IGME-Child-Mortality-Report-2018.pdf
9. World Health Organization (WHO). *Roadmap on Human Resource Strategies to Improve Newborn Care in Health Facilities in Low and Middle-Income Countries.* WHO; 2020. Accessed December 22, 2020. https://apps.who.int/iris/bitstream/handle/10665/336677/9789240015227-eng.pdf
10. Aluvaala J, Nyamai R, Were F, et al.; SIRCLE/Ministry of Health Hospital Survey Group. Assessment of neonatal care in clinical training facilities in Kenya. *Arch Dis Child.* 2015;100(1):42–47. CrossRef. Medline
11. Neogi SB, Khanna R, Chauhan M, et al. Inpatient care of small and sick newborns in healthcare facilities. *J Perinatol.* 2016;36(S3)(s3):S18–S23. CrossRef. Medline
12. Moxon SG, Lawn JE, Dickson KE, et al. Inpatient care of small and sick newborns: a multi-country analysis of health system bottlenecks and potential solutions. *BMC Pregnancy Childbirth.* 2015;15(S2)(Suppl 2):S7. CrossRef. Medline
13. Every Woman Every Child (EWEC). T*he Global Strategy for Women's, Children's, and Adolescents' Health (2016–2030): Survive, Thrive, Transform.* EWEC; 2015. Accessed December 22, 2020. http://www.everywomaneverychild.org/global-strategy/
14. World Health Organization (WHO). *Global Strategic Directions for Strengthening Nursing and Midwifery 2016–2020.* WHO; 2016. https://www.who.int/hrh/nursing_midwifery/global-strategy-midwifery-2016-2020/en/
15. Bell SA, Rominski S, Bam V, Donkor E, Lori J. Analysis of nursing education in Ghana: priorities for scaling-up the nursing workforce. *Nurs Health Sci.* 2013;15(2):244–249. CrossRef. Medline
16. World Bank. World Development Indicators. Data Bank. Accessed December 22, 2020. https://databank.worldbank.org/reports.aspx?source=2&series=SH.MED.NUMW.P3&country=
17. Sharma G, Mathai M, Dickson KE, et al. Quality care during labour and birth: a multi-country analysis of health system bottlenecks and potential solutions. *BMC Pregnancy Childbirth.* 2015;15(S2)(Suppl 2):S2. CrossRef. Medline
18. World Health Organization (WHO). *2018 Progress Report: Reaching Every Newborn National 2020 Milestones.* WHO; 2018. Accessed December 22, 2020. https://www.healthynewbornnetwork.org/hnn-content/uploads/Final-Country-Progress-Report-v9-low-res.pdf
19. Roberton T, Carter ED, Chou VB, et al. Early estimates of the indirect effects of the COVID-19 pandemic on maternal and child mortality in low-income and middle-income countries: a modelling study. *Lancet Glob Health.* 2020;8(7):e901–e908. CrossRef. Medline

20. Kruk ME, Pate M. The Lancet Global Health Commission on High Quality Health Systems 1 year on: progress on a global imperative. *Lancet Glob Health*. 2020;8(1):e30–e32. CrossRef. Medline
21. World Health Organization (WHO). *The World Health Report 2006: Working Together for Health*. WHO; 2006. Accessed December 22, 2020. https://www.who.int/whr/2006/en/
22. World Health Organization (WHO), UNICEF. *Ending Preventable Newborn Deaths and Stillbirths by 2030*. WHO, UNICEF; 2020. Accessed December 22, 2020. https://www.healthynewbornnetwork.org/hnn-content/uploads/Final-draft-Every-Newborn-Coverage-target-Milestones-to-2025.pdf
23. World Health Organization (WHO). *Standards for Improving the Quality of Care for Small and Sick Newborns in Health Facilities*. WHO; 2020. Accessed December 22, 2020. https://www.who.int/publications/i/item/9789240010765
24. Tunçalp Ö, Were WM, MacLennan C, et al. Quality of care for pregnant women and newborns—the WHO vision. *BJOG*. 2015;122(8):1045–1049. CrossRef. Medline
25. Levac D, Colquhoun H, O'Brien KK. Scoping studies: advancing the methodology. *Implement Sci*. 2010;5(1):69. CrossRef. Medline
26. Arksey H, O'Malley L. Scoping studies: towards a methodological framework. *Int J Soc Res Methodol*. 2005;8(1):19–32. CrossRef
27. The World Bank. World Bank country and lending groups. Accessed December 23, 2020. https://datahelpdesk.worldbank.org/knowledgebase/articles/906519-world-bank-country-and-lending-groups
28. Braun V, Clarke V. Using thematic analysis in psychology. *Qual Res Psychol*. 2006;3(2):77–101. CrossRef
29. Martinez AM, Khu DTK, Boo NY, Neou L, Saysanasongkham B, Partridge JC. Barriers to neonatal care in developing countries: parents' and providers' perceptions. *J Paediatr Child Health*. 2012;48(9):852–858. CrossRef. Medline
30. Ministry of Health Ghana. Ghana National Newborn Health Strategy and Action Plan 2014-2018. Accessed December 23, 2020. CrossRef
31. Neogi SB, Malhotra S, Zodpey S, Mohan P. Challenges in scaling up of special care newborn units-lessons from India. *Indian Pediatr*. 2011;48(12):931–935. CrossRef. Medline
32. Getachew A, Ricca J, Cantor D, et al. Quality of Care for Prevention and Management of Common Maternal and Newborn Complications: A Study of Ethiopia's Hospitals. Jhpiego; 2011. Accessed December 23, 2020. https://www.healthynewbornnetwork.org/hnn-content/uploads/Ethiopia_Qualtiy-of-Care-Assement-report_MCHIP_final-1.pdf
33. National Research Council. *Reducing Maternal and Neonatal Mortality in Indonesia: Saving Lives, Saving the Future*. National Academies Press; 2013. CrossRef
34. Mbonye AK, Sentongo M, Mukasa GK, et al.; Uganda Decade of Change and Future Implications Analysis Group. Newborn survival in Uganda: a decade of change and future implications. *Health Policy Plan*. 2012;27(suppl_3):iii104–iii117. CrossRef. Medline
35. Adegoke AA, Atiyaye FB, Abubakar AS, Auta A, Aboda A. Job satisfaction and retention of midwives in rural Nigeria. *Midwifery*. 2015;31(10):946–956. CrossRef. Medline
36. Kildea S, Larsson M, Govind S. A review of midwifery in Mongolia utilising the 'Strengthening Midwifery Toolkit'. *Women Birth*. 2012;25(4):166–173. CrossRef. Medline
37. Bogren M, Ndela B, Toko C, Berg M. Midwifery education, regulation and association in the Democratic Republic of Congo (DRC)—current state and challenges. *Glob Health Action*. 2020;13(1):1717409. CrossRef. Medline
38. Elikplim Pomevor K, Adomah-Afari A. Health providers' perception of quality of care for neonates in health facilities in a municipality in Southern Ghana. *Int J Health Care Qual Assur*. 2016;29(8):907–920. CrossRef. Medline
39. Campbell-Yeo M, Deorari A, McMillan DD, et al. Educational barriers of nurses caring for sick and at-risk infants in India. *Int Nurs Rev*. 2014;61(3):398–405. CrossRef. Medline
40. Malhotra S, Zodpey SP, Vidyasagaran AL, et al. Assessment of essential newborn care services in secondary-level facilities from two districts of India. *J Health Popul Nutr*. 2014;32(1):130–141. CrossRef. Medline
41. Haile-Mariam A, Tesfaye N, Otterness C, Bailey PE. Assessing the health system's capacity to conduct neonatal resuscitation in Ethiopia. *Ethiop Med J*. 2012;50(1):43–55. Medline
42. Bream KDW, Gennaro S, Kafulafula U, Mbweza E, Hehir D. Barriers to and facilitators for newborn resuscitation in Malawi, *Africa. J Midwifery Womens Health*. 2005;50(4):329–334. CrossRef. Medline
43. Kozuki N, Oseni L, Mtimuni A, et al. Health facility service availability and readiness for intrapartum and immediate postpartum care in Malawi: a cross-sectional survey. *PLoS One*. 2017;12(3):e0172492. CrossRef. Medline
44. Jirapaet V, Jirapaet K, Sopajaree C. The nurses' experience of barriers to safe practice in the neonatal intensive care unit in Thailand. *J Obstet Gynecol Neonatal Nurs*. 2006;35(6):746–754. CrossRef. Medline
45. Cole-Ceesay R, Cherian M, Sonko A, et al. Strengthening the emergency healthcare system for mothers and children in The Gambia. *Reprod Health*. 2010;7(1):21. CrossRef. Medline
46. Michel-Schuldt M, Billy Dayon M, Toft Klar R, et al. Continuous professional development of Liberia's midwifery workforce—a coordinated multi-stakeholder approach. *Midwifery*. 2018;62:77–80. CrossRef. Medline
47. Nyamtema AS, Urassa DP, Massawe S, Massawe A, Lindmark G, Van Roosmalen J. Staffing needs for quality perinatal care in Tanzania. *Afr J Reprod Health*. 2008;12(3):113–124. Medline
48. Nzinga J, McKnight J, Jepkosgei J, English M. Exploring the space for task shifting to support nursing on neonatal wards in Kenyan public hospitals. *Hum Resour Health*. 2019;17(1):18. CrossRef. Medline
49. Ntigurirwa P, Mellor K, Langer D, et al. A health partnership to reduce neonatal mortality in four hospitals in Rwanda. *Global Health*. 2017;13(1):28. CrossRef. Medline
50. Fischer EA, Jayana K, Cunningham T, et al. Nurse mentors to advance quality improvement in primary health centers: lessons from a pilot program in Northern Karnataka, *India. Glob Health Sci Pract*. 2015;3(4):660–675. CrossRef. Medline
51. Amin AA, Vankar JR, Nimbalkar SM, Phatak AG. Perceived stress and professional quality of life in neonatal intensive care unit nurses in Gujarat, India. *Indian J Pediatr*. 2015;82(11):1001–1005. CrossRef. Medline
52. Ith P, Dawson A, Homer CSE, Klinken Whelan A. Practices of skilled birth attendants during labour, birth and the immediate postpartum period in Cambodia. *Midwifery*. 2013;29(4):300–307. CrossRef. Medline
53. Prytherch H, Kakoko DCV, Leshabari MT, Sauerborn R, Marx M. Maternal and newborn healthcare providers in rural Tanzania: in-depth interviews exploring influences on motivation, performance and job satisfaction. *Rural Remote Health*. 2012;12(3):2072. Medline
54. Allen CW, Jeffery H. Implementation and evaluation of a neonatal educational program in rural Nepal. *J Trop Pediatr*. 2006;52(3):218–222. CrossRef. Medline
55. Tosif S, Nasi T, Gray A, Sadr-Azodi N, Ogaoga D, Duke T. Assessment of the quality of neonatal care in the Solomon Islands. *J Paediatr Child Health*. 2018;54(2):165–171. CrossRef. Medline

56. Neogi SB, Malhotra S, Zodpey S, Mohan P. Assessment of special care newborn units in India. *J Health Popul Nutr*. 2011;29(5):500–509. CrossRef. Medline

57. Dewez JE, Chellani H, Nangia S, et al. Healthcare workers' views on the use of continuous positive airway pressure (CPAP) in neonates: a qualitative study in Andhra Pradesh, *India. BMC Pediatr*. 2018; 18(1):347. CrossRef. Medline

58. Morgan MC, Dyer J, Abril A, et al. Barriers and facilitators to the provision of optimal obstetric and neonatal emergency care and to the implementation of simulation-enhanced mentorship in primary care facilities in Bihar, India: a qualitative study. *BMC Pregnancy Childbirth*. 2018;18(1):420. CrossRef. Medline

59. Horiuchi S, Rattana S, Saysanasongkham B, et al. Study protocol of a cluster randomized controlled trial to evaluate effectiveness of a system for maintaining high-quality early essential newborn care in Lao PDR. *BMC Health Serv Res*. 2018;18(1):489. CrossRef. Medline

60. Sychareun V, Phommachanh S, Soysouvanh S, et al. Provider perspectives on constraints in providing maternal, neonatal and child health services in the Lao People's democratic republic: a qualitative study. *BMC Pregnancy Childbirth*. 2013;13(1):243. CrossRef. Medline

61. Brunson J. Confronting maternal mortality, controlling birth in Nepal: the gendered politics of receiving biomedical care at birth. *Soc Sci Med*. 2010;71(10):1719–1727. CrossRef. Medline

62. Moxon SG, Ruysen H, Kerber KJ, et al. Count every newborn; a measurement improvement roadmap for coverage data. *BMC Pregnancy Childbirth*. 2015;15(S2)(Suppl 2):S8. CrossRef. Medline

63. World Health Organization (WHO). Transforming and Scaling Up Health Professional Education and Training. Policy Brief on Regulation of Health Professions Education. WHO; 2013, Accessed December 23, 2020. https://apps.who.int/iris/bitstream/handle/10665/93635/9789241506502_eng.pdf?sequence=1

64. Kalyan G, Vatsa M. Neonatal nursing: an unmet challenge in India. *Indian J Pediatr*. 2014;81(11):1205–1211. CrossRef. Medline

65. Hudspeth J, Curry CL, Sacks Z, Surena C. Continuing professional development in low-resource settings: Haiti as example. *Ann Glob Health*. 2015;81(2):255–259. CrossRef. Medline

66. Bolan NE, Sthreshley L, Ngoy B, et al. mLearning in the Democratic Republic of the Congo: a mixed-methods feasibility and pilot cluster randomized trial using the safe delivery app. *Glob Health Sci Pract*. 2018;6(4):693–710. CrossRef. Medline

67. Rowe AK, de Savigny D, Lanata CF, Victora CG. How can we achieve and maintain high-quality performance of health workers in low-resource settings? *Lancet*. 2005;366(9490):1026–1035. CrossRef. Medline

68. Nilsson C, Sørensen BL, Sørensen JL. Comparing hands-on and video training for postpartum hemorrhage management. *Acta Obstet Gynecol Scand*. 2014;93(5):517–520. CrossRef. Medline

69. World Health Organization (WHO). *Global Strategy on Human Resources for Health: Workforce 2030*. WHO; 2016. Accessed December 23, 2020. https://www.who.int/hrh/resources/globstrathrh-2030/en/

70. Bolan NE, Newman SD, Nemeth LS. Technology-based newborn health learning initiatives for facility-based nurses and midwives in low-and middle-income countries: a scoping review. *Int J Childbirth*. 2018;8(4):252–268. CrossRef

71. World Health Organization (WHO). Every Newborn: An Action Plan to End Preventable Deaths. WHO; 2014. Accessed December 23, 2020. https://www.who.int/maternal_child_adolescent/documents/every-newborn-action-plan/en/

72. Gupta N, Maliqi B, França A, et al. Human resources for maternal, newborn and child health: from measurement and planning to performance for improved health outcomes. *Hum Resour Health*. 2011;9(1):16. CrossRef. Medline

73. Smith SL, Hall MA. Advanced neonatal nurse practitioners in the workforce: a review of the evidence to date. *Arch Dis Child Fetal Neonatal Ed*. 2011;96(2):F151–F155. CrossRef. Medline

74. Rogowski JA, Staiger D, Patrick T, Horbar J, Kenny M, Lake ET. Nurse staffing and NICU infection rates. *JAMA Pediatr*. 2013;167(5):444–450. CrossRef. Medline

75. Küng E, Waldhör T, Rittenschober-Böhm J, Berger A, Wisgrill L. Increased nurse workload is associated with bloodstream infections in very low birth weight infants. *Sci Rep*. 2019;9(1):6331. CrossRef. Medline

76. Watson SI, Arulampalam W, Petrou S, et al.; Neonatal Data Analysis Unit (NDAU) and the Neonatal Economic, Staffing, and Clinical Outcomes Project (NESCOP) Group. The effects of a one-to-one nurse-to-patient ratio on the mortality rate in neonatal intensive care: a retrospective, longitudinal, population-based study. *Arch Dis Child Fetal Neonatal Ed*. 2016;101(3):F195–F200. CrossRef. Medline

77. Hamilton KE, Redshaw ME, Tarnow-Mordi W. Nurse staffing in relation to risk-adjusted mortality in neonatal care. *Arch Dis Child Fetal Neonatal Ed*. 2007;92(2):F99–F103. CrossRef. Medline

78. BLISS. The chance of a lifetime? BLISS Baby Report 2010. *BLISS*. 2010;(8):8–10. Accessed January 21, 2021. https://issuu.com/bliss-charity/docs/baby_report_2010_web

79. Buchan J, Shaffer FA, Catton H. *Policy Brief: Nurse Retention*. International Centre on Nursing Migration; 2018. Accessed December 23, 2020. https://www.icn.ch/sites/default/files/inline-files/2018_ICNM%20Nurse%20retention.pdf

80. Filby A, McConville F, Portela A. What prevents quality midwifery care? A systematic mapping of barriers in low and middle income countries from the provider perspective. *PLoS One*. 2016;11(5):e0153391. CrossRef. Medline

81. Manasyan A, Saleem S, Koso-Thomas M, et al.; EmONC Trial Group. Assessment of obstetric and neonatal health services in developing country health facilities. *Am J Perinatol*. 2013;30(09):787–794. CrossRef. Medline

82. Franco LM, Bennett S, Kanfer R. Health sector reform and public sector health worker motivation: a conceptual framework. *Soc Sci Med*. 2002;54(8):1255–1266. CrossRef. Medline

83. Dieleman M, Toonen J, Touré H, Martineau T. The match between motivation and performance management of health sector workers in Mali. *Hum Resour Health*. 2006;4(1):2. CrossRef. Medline

84. Thi Hoai Thu N, Wilson A, McDonald F. Motivation or demotivation of health workers providing maternal health services in rural areas in Vietnam: findings from a mixed-methods study. *Hum Resour Health*. 2015;13(1):91. CrossRef. Medline

85. Fonn S, Ray S, Blaauw D. Innovation to improve health care provision and health systems in sub-Saharan Africa—promoting agency in mid-level workers and district managers. *Glob Public Health*. 2011; 6(6):657–668. CrossRef. Medline

86. Inoue KC, Gomes da Silva Versa GL, Misue Matsuda L. Stress level among intensive care nurses in the municipality of Paraná (Brazil). *Invest Educ Enferm*. 2014;32(1):69–77. CrossRef. Medline

87. Campbell J, Cometto G, Rasanathan K, et al. Improving the resilience and workforce of health systems for women's, children's, and adolescents' health. *BMJ*. 2015;351:h4148. CrossRef. Medline

88. World Health Organization (WHO). *Midwives' Voices Midwives' Realities*. WHO; 2016. Accessed December 23, 2020. https://www.who.int/maternal_child_adolescent/documents/midwives-voices-realities/en/

89. Gallagher A. Moral distress and moral courage in everyday nursing practice. *Online J Issues Nurs*. 2011;16(2):8. CrossRef. Medline

90. Campbell J. The route to effective coverage is through the health worker: there are no shortcuts. *Lancet*. 2013;381(9868):725. CrossRef. Medline

Peer Reviewed

Received: July 3, 2020; **Accepted:** December 9, 2020; **First published online:** xxx

Cite este artículo: Bolan N, Cowgill KD, Walker K, et al. Desafíos de los recursos humanos en el área de salud para garantizar atención neonatal de calidad en países con ingresos bajos y medianos: una revisión exploratoria. *Glob Health Sci Pract.* 2021;9(1). https://doi.org/10.9745/GHSP-D-20-00362.

© Bolan et al. Este es un articulo de acceso libre, distribuido bajo los términos de la licencia de atribución de Creative Commons, la cual permite, en cualquier soporte, el uso, la distribución, y la reproducción no restringido, siempre y cuando se cite de manera adecuada el autor original. Para ver una copia de dicha licencia, consulte http://creativecommons.org/licenses/by/4.0/. https://doi.org/10.9745/GHSP-D-20-00362
